# Tissue Oxygenation Changes After Transfusion and Outcomes in Preterm Infants: A Secondary Near-Infrared Spectroscopy Study of the Transfusion of Prematures Randomized Clinical Trial (TOP NIRS)

**DOI:** 10.1001/jamanetworkopen.2023.34889

**Published:** 2023-09-21

**Authors:** Valerie Y. Chock, Haresh Kirpalani, Edward F. Bell, Sylvia Tan, Susan R. Hintz, M. Bethany Ball, Emily Smith, Abhik Das, Yvonne C. Loggins, Beena G. Sood, Lina F. Chalak, Myra H. Wyckoff, Stephen D. Kicklighter, Kathleen A. Kennedy, Ravi M. Patel, Waldemar A. Carlo, Karen J. Johnson, Kristi L. Watterberg, Pablo J. Sánchez, Abbot R. Laptook, Ruth B. Seabrook, C. Michael Cotten, Toni Mancini, Gregory M. Sokol, Robin K. Ohls, Anna Maria Hibbs, Brenda B. Poindexter, Anne Marie Reynolds, Sara B. DeMauro, Sanjay Chawla, Mariana Baserga, Michele C. Walsh, Rosemary D. Higgins, Krisa P. Van Meurs

**Affiliations:** 1 Division of Neonatal and Developmental Medicine, Department of Pediatrics, Stanford University School of Medicine and Lucile Packard Children’s Hospital, Palo Alto, California; 2 Department of Pediatrics, University of Pennsylvania, Philadelphia; 3 Department of Pediatrics, University of Iowa, Iowa City; 4 Social, Statistical and Environmental Sciences Unit, RTI International, Research Triangle Park, North Carolina; 5 Social, Statistical and Environmental Sciences Unit, RTI International, Rockville, Maryland; 6 Department of Pediatrics, Children’s Healthcare of Atlanta, Emory University School of Medicine, Atlanta, Georgia; 7 Department of Pediatrics, Wayne State University, Detroit, Michigan; 8 Department of Pediatrics, University of Texas Southwestern Medical Center, Dallas; 9 Division of Neonatology, Department of Pediatrics, WakeMed Health and Hospitals, Raleigh, North Carolina; 10 Department of Pediatrics, McGovern Medical School at The University of Texas Health Science Center at Houston, Houston; 11 Division of Neonatology, University of Alabama at Birmingham, Birmingham; 12 University of New Mexico Health Sciences Center, Albuquerque; 13 Department of Pediatrics, Nationwide Children’s Hospital, The Ohio State University College of Medicine, Columbus; 14 Department of Pediatrics, Women & Infants Hospital, Brown University, Providence, Rhode Island; 15 Department of Pediatrics, Duke University, Durham, North Carolina; 16 Department of Pediatrics, Indiana University School of Medicine, Indianapolis; 17 Division of Neonatology, Department of Pediatrics, University of Utah School of Medicine, Salt Lake City; 18 Department of Pediatrics, Rainbow Babies & Children’s Hospital, Case Western Reserve University, Cleveland, Ohio; 19 Department of Pediatrics, Cincinnati Children’s Hospital Medical Center, University of Cincinnati College of Medicine, Cincinnati, Ohio; 20 Department of Pediatrics, University of Buffalo Women’s and Children’s Hospital of Buffalo, Buffalo, New York; 21 Children’s Hospital of Philadelphia, Philadelphia, Pennsylvania; 22 Eunice Kennedy Shriver National Institute of Child Health and Human Development, National Institutes of Health, Bethesda, Maryland; 23 Research and Sponsored Programs, Florida Gulf Coast University, Fort Myers

## Abstract

**Importance:**

Preterm infants with varying degrees of anemia have different tissue oxygen saturation responses to red blood cell (RBC) transfusion, and low cerebral saturation may be associated with adverse outcomes.

**Objective:**

To determine whether RBC transfusion in preterm infants is associated with increases in cerebral and mesenteric tissue saturation (Csat and Msat, respectively) or decreases in cerebral and mesenteric fractional tissue oxygen extraction (cFTOE and mFTOE, respectively) and whether associations vary based on degree of anemia, and to investigate the association of Csat with death or neurodevelopmental impairment (NDI) at 22 to 26 months corrected age.

**Design, Setting, and Participants:**

This was a prospective observational secondary study conducted among a subset of infants between August 2015 and April 2017 in the Transfusion of Prematures (TOP) multicenter randomized clinical trial at 16 neonatal intensive care units of the Eunice Kennedy Shriver National Institute of Child Health and Human Development Neonatal Research Network. Preterm neonates with gestational age 22 to 28 weeks and birth weight 1000 g or less were randomized to higher or lower hemoglobin thresholds for transfusion. Data were analyzed between October 2020 and May 2022.

**Interventions:**

Near-infrared spectroscopy monitoring of Csat and Msat.

**Main Outcomes and Measures:**

Primary outcomes were changes in Csat, Msat, cFTOE, and mFTOE after transfusion between hemoglobin threshold groups, adjusting for age at transfusion, gestational age, birth weight stratum, and center. Secondary outcome at 22 to 26 months was death or NDI defined as cognitive delay (Bayley Scales of Infant and Toddler Development-III score <85), cerebral palsy with Gross Motor Function Classification System level II or greater, or severe vision or hearing impairment.

**Results:**

A total of 179 infants (45 [44.6%] male) with mean (SD) gestational age 25.9 (1.5) weeks were enrolled, and valid data were captured from 101 infants during 237 transfusion events. Transfusion was associated with a significant increase in mean Csat of 4.8% (95% CI, 2.7%-6.9%) in the lower–hemoglobin threshold group compared to 2.7% (95% CI, 1.2%-4.2%) in the higher–hemoglobin threshold group, while mean Msat increased 6.7% (95% CI, 2.4%-11.0%) vs 5.6% (95% CI, 2.7%-8.5%). Mean cFTOE and mFTOE decreased in both groups to a similar extent. There was no significant change in peripheral oxygen saturation (SpO_2_) in either group (0.2% vs −0.2%). NDI or death occurred in 36 infants (37%). Number of transfusions with mean pretransfusion Csat less than 50% was associated with NDI or death (odds ratio, 2.41; 95% CI, 1.08-5.41; *P* = .03).

**Conclusions and Relevance:**

In this secondary study of the TOP randomized clinical trial, Csat and Msat were increased after transfusion despite no change in SpO_2_. Lower pretransfusion Csat may be associated with adverse outcomes, supporting further investigation of targeted tissue saturation monitoring in preterm infants with anemia.

**Trial Registration:**

ClinicalTrials.gov Identifier: NCT01702805

## Key Points

**Question:**

After red blood cell (RBC) transfusion, does tissue oxygen saturation vary depending on degree of anemia, and is cerebral saturation associated with neurodevelopmental outcomes?

**Findings:**

In this secondary analysis of the Transfusion of Prematures (TOP) randomized clinical trial, mean cerebral saturation (Csat) and mesenteric saturation (Msat) were significantly increased after RBC transfusion in 2 hemoglobin threshold groups, with no statistical difference in the magnitude of increase between groups. Mean pretransfusion Csat less than 50% occurred more frequently in infants who died or developed neurodevelopmental impairment at 22 to 26 months corrected age.

**Meaning:**

The findings indicate that increases in Csat and Msat are associated with RBC transfusion and that Csat may be a useful target for improving survival without neurodevelopmental impairment in infants with various degrees of anemia.

## Teaser

This secondary analysis of the TOP NIRS randomized clinical trial evaluates associations of red blood cell transfusion and low cerebral saturation with outcomes in preterm infants.

## Introduction

Anemia of prematurity is frequent in infants with extremely low birth weight. These infants are among those who most frequently undergo transfusions in neonatal intensive care units^1^ due to several postulated causes: immaturity of the hematopoietic system, iatrogenic losses from blood sampling, increased oxygen consumption, and critical illness requiring enhanced oxygen delivery. An optimal transfusion threshold is needed to balance risks of anemia with risks of red blood cell (RBC) transfusions. Two large randomized clinical trials,^2,3^ including the Transfusion of Prematures (TOP) trial,^2^ found that a higher hemoglobin (Hgb) threshold for transfusion did not improve survival without neurodevelopmental impairment (NDI) in infants with extremely low birth weight. However, Hgb (or hematocrit) may not be the best measure to establish transfusion needs. End organ tissue saturation provides an alternative, individualized indication of need for RBC transfusion. Tissue saturation can be continuously and noninvasively assessed using near-infrared spectroscopy (NIRS) to measure the balance between oxygen delivery and consumption. NIRS may be a useful bedside monitoring tool in infants with extremely low birth weight and anemia.

Following RBC transfusions, preterm infants exhibit minimal change in peripheral oxygen saturation (SpO_2_) but may experience an increase in regional tissue oxygen saturation.^4^ Cerebral oxygen saturation (Csat) and mesenteric oxygen saturation (Msat) have been shown to increase following transfusion, whereas cerebral and mesenteric fractional tissue oxygen extraction (cFTOE and mFTOE) have been shown to decrease.^4,5,6,7,8^ However, transfusion-associated changes may differ based on degree of anemia, as Csat has been reported not to increase using a liberal transfusion threshold.^9^ The extent to which transfusion threshold affects changes in tissue oxygen saturation remains unclear.

Tissue oxygen saturation and response to transfusion may also have long-term implications. A higher burden of cerebral hypoxia has been associated with severe intracranial hemorrhage and abnormal electroencephalography findings.^10^ However, achieving improved neurodevelopmental outcomes by targeting optimal Csat levels in preterm infants remains controversial.^11,12,13^ As a patient-specific indicator of brain perfusion, cerebral NIRS measures may be associated with neurodevelopmental outcomes and may better guide transfusion thresholds than Hgb values.

In a secondary prospective observational study (TOP NIRS) of a subset of infants enrolled in the Eunice Kennedy Shriver National Institute of Child Health and Human Development Neonatal Research Network TOP trial,^2^ we hypothesized that NIRS measures of Csat and Msat would increase after a transfusion and corresponding cFTOE and mFTOE would decrease, with greater changes for those with a lower transfusion threshold. As a hypothesis-generating objective, we further explored the association of cerebral NIRS measures and other clinical factors with the outcome of death or NDI.

## Methods

### Study Population

The trial protocol is in Supplement 1. The TOP trial randomized preterm infants of birth weight 1000 g or less and gestational age between 22 weeks 0 days and 28 weeks 6 days within 48 hours of birth to receive RBC transfusions at higher or lower Hgb thresholds.^2^ Thresholds were aligned with current practice^14^ and determined by postnatal age (highest in week 1 and lower in week 2 and beyond week 3) and according to degree of respiratory support (higher if on mechanical ventilation, continuous positive airway pressure, fraction of inspired oxygen >0.35, or nasal cannula flow ≥1 L/min) (eTable 1 in Supplement 2).

Infants were eligible for the observational secondary study of NIRS monitoring between August 1, 2015, and April 12, 2017, if they were concurrently enrolled in the TOP trial. Parental informed consent was sought unless skin integrity was deemed inadequate for NIRS sensor placement for the duration of the infant’s enrollment in the trial or if any TOP exclusion criteria were present (cyanotic congenital heart disease, nonviability as deemed by attending neonatologist, in utero fetal transfusion, twin-to-twin transfusion syndrome, isoimmune hemolytic disease, congenital condition other than premature birth that adversely affects life expectancy or neurodevelopment, parents opposed to the transfusion of blood, parents with hemoglobinopathy or congenital anemia, prior blood transfusion beyond the first 6 hours of life, or high probability that the family would not be able to return for follow-up at 22-26 months). Institutional review board approval was obtained at each site, and multisite training was conducted prior to study enrollment. The study followed the Strengthening the Reporting of Observational Studies in Epidemiology (STROBE) reporting guideline.

### NIRS Monitoring

Neonatal sensors were applied to the right or left forehead and to the lower left abdominal quadrant for the first week of life and then reapplied for 48 hours during subsequent RBC transfusions to monitor Csat and Msat with NIRS (INVOS 5100C [Medtronic]). A Mepitel (Molnycke) skin dressing was used for skin protection under the sensors and has previously been shown not to interfere with validity of NIRS measurements.^15^ Synchronized NIRS and pulse oximetry (Nellcor [Medtronic]) data were collected with a Vital Sync device (Medtronic), and monitor screens were obscured to mask clinicians to NIRS measures. Sensors were assessed routinely to evaluate surrounding skin integrity and replaced every 4 days or as needed. Sensors were removed after 7 days of life or 48 hours after completion of transfusion. All data were downloaded to electronic media and securely transferred to the data coordinating center at RTI International.

### Data Processing

Csat, Msat, and SpO_2_ measures were acquired every 30 seconds. Pretransfusion measures were averaged in the 1 hour prior to start of transfusion, and posttransfusion measures were averaged in the 1 hour after completion of transfusion. Both periods required at least 10 minutes of consecutive data. If missing, pretransfusion NIRS measures were established up to 15 minutes after start of a transfusion and posttransfusion measures up to 4 hours after transfusion completion, given minimal variability in early and posttransfusion NIRS measures.^5,16^ In addition to these definitions of valid pretransfusion and posttransfusion NIRS measures, oxygen extraction values were calculated as cFTOE = (SpO_2_ − Csat) / SpO_2_ and mFTOE = (SpO_2_ − Msat) / SpO_2_. Outlying values, determined by a greater than 50% change in successive measures and by negative cFTOE or mFTOE measurements, were not included in analysis.

### Outcomes

The primary outcomes were changes in NIRS measures (Csat, Msat, cFTOE, and mFTOE) from pretransfusion to posttransfusion. The secondary outcome was a composite of death or NDI at 22 to 26 months of age corrected for prematurity, with NDI defined as cognitive delay (Bayley Scales of Infant and Toddler Development-III score <85), cerebral palsy with Gross Motor Function Classification System level II or greater, or severe vision or hearing impairment.

### Statistical Analysis

Analyses occurred between October 2020 and May 2022. The difference in each outcome between Hgb threshold groups was examined using mixed-effects modeling performed on transfusion-level data using a random intercepts model while adjusting for age at transfusion, gestational age, birth weight stratum,^2^ and the clustering of measurements within infant. Data for each model were limited to NIRS-monitored transfusions over the first 28 days after birth, corresponding to the TOP trial period with greatest number of transfusions and Hgb instability.^2^ Changes in NIRS measures over time and by treatment group were illustrated by restricted cubic spline plots.

Classification and regression tree (CART) analysis was used to explore infant-level prognostic factors for death or NDI over the entire hospitalization period. Variables offered to the CART procedure are listed in eTable 2 in Supplement 2 and include transfusion-related measures until 36 weeks’ postmenstrual age (NIRS measures, Hgb, and transfusion characteristics) as well as clinical measures (gestational age, sex, days of ventilation, hospital comorbidities).^17,18^ The final classification tree was limited to the top 3 predictors found to be most associated with death or NDI.

To validate results of CART, a stepwise logistic regression was performed, offering similar infant-level variables (eTable 2 in Supplement 2) to the procedure. The α criterion for a variable to enter the model was .15, with a criterion of .10 required for a variable to stay in the model. The final model was adjusted for gestational age, sex, and the clustering of infants within center as a random effect, using generalized linear mixed modeling. The area under the receiver operating characteristic curves was compared between the CART and logistic regression procedures. Further post hoc exploration used descriptive statistics and Wilcoxon nonparametric tests. All analyses were performed using SAS version 9.4 (SAS Institute)—except the restricted cubic spline analysis, which used Stata version 17 (StataCorp)—with a 2-tailed *P* value less than .05 indicating statistical significance.

## Results

Among 16 participating centers, 179 infants (45 [44.6%] male) with mean (SD) gestational age 25.9 (1.5) weeks were enrolled, and 140 had RBC transfusions (eFigure in Supplement 2). When restricted to transfusions with both valid pretransfusion and posttransfusion NIRS data, there remained 101 infants with a total of 237 transfusion events captured. For individuals with nonvalid cerebral NIRS data, 73 transfusion events had less than 10 minutes of continuous pretransfusion NIRS data, and 26 additional transfusion events had less than 10 minutes of continuous posttransfusion NIRS data. There were no significant differences in demographic or perinatal variables between Hgb threshold groups (Table 1). The mean (SE) pretransfusion Hgb was 11.0 (0.1) g/dL in the higher threshold group compared to 9.0 (0.2) g/dL in the lower threshold group. The mean number of NIRS-monitored transfusions per infant was 2 (range, 1-12) during the study period (birth until 36 weeks’ postmenstrual age); this was not significantly different between threshold groups: mean (SD) 2.5 (2.4) in the higher-threshold group and 2.0 (1.9) in the lower-threshold group.

**Table 1.** Perinatal and Transfusion Characteristics (N = 101)

Variable	No. (%)		
	All participants (n = 101)	High-threshold group (n = 65)	Low-threshold group (n = 36)
Perinatal characteristic			
Maternal racial or ethnic group^a^			
Asian	3 (3)	1 (2)	2 (6)
Non-Hispanic Black	26 (26)	21 (32)	5 (14)
Hispanic	40 (40)	24 (37)	16 (44)
Non-Hispanic White	24 (24)	14 (22)	10 (28)
Other^b^	8 (8)	5 (8)	3 (8)
Antenatal steroids	92 (91)	60 (92)	32 (89)
Cesarean delivery	76 (75)	47 (72)	29 (81)
Inborn^c^	93 (92)	59 (91)	34 (94)
Gestational age, mean (SD), wk	26 (1.5)	25.9 (1.5)	25.9 (1.4)
Small for gestational age	14 (14)	9 (14)	5 (14)
Birth weight, mean (SD), g	766 (149)	768 (159)	761 (131)
Head circumference at birth, mean (SD), cm^d^	22.9 (1.8)	22.8 (2.0)	23.0 (1.5)
Sex			
Female	56 (55)	35 (54)	21 (58)
Male	45 (45)	30 (46)	15 (43)
Delayed cord clamping	28 (28)	18 (28)	10 (29)
Umbilical cord milking	6 (6)	5 (8)	1 (3)
Delivery room resuscitation			
Intubation	62 (61)	41 (63)	21 (58)
Chest compressions	5 (5)	3 (5)	2 (6)
Epinephrine	4 (4)	2 (3)	2 (6)
5-min Apgar score ≤5	21 (21)	14 (22)	7 (19)
SNAPPE score, mean (SD)^e^	49.0 (20.4)	49.6 (20.4)	48.0 (20.7)
RBC transfusion characteristics			
Pretransfusion hemoglobin, mean (SE), g/dL^f^	10.4 (0.1)	11.0 (0.1)	9.0 (0.2)
Transfused in first week	53 (52)	31 (48)	22 (61)
RBC transfusions per individual, mean (SD)	2.3 (2.2)	2.5 (2.4)	2.0 (1.9)
Transfusion-specific properties, total count^g^	232	160	72
Enteral feeding during transfusion	80 (34)	57 (36)	23 (32)
Hypocarbia (pCO_2_ < 40 mm Hg) at transfusion^h^	11 (6)	8 (6)	3 (5)
Concurrent medications at transfusion			
Pressor support^i^	15 (6)	11 (7)	4 (6)
Indomethacin	6 (3)	4 (3)	2 (3)
Steroids	6 (3)	2 (1)	4 (6)
Respiratory support (primary mode) during transfusion			
High-frequency ventilation	21 (9)	17 (11)	4 (6)
Conventional ventilation	37 (16)	25 (16)	12 (17)
Nasal SIMV/SiPAP/NIPPV	6 (3)	2 (1)	4 (6)
CPAP/high-flow nasal cannula	16 (7)	13 (8)	3 (4)
Nasal cannula/hood	1 (0)	1 (1)	0
No respiratory support	151 (65)	102 (64)	49 (68)

Abbreviations: CPAP, continuous positive airway pressure; NIPPV, nasal intermittent positive pressure ventilation; pCO_2_, partial pressure of carbon dioxide; RBC, red blood cell; SIMV, synchronized intermittent mandatory ventilation; SiPAP synchronized inspiratory positive airway pressure; SNAPPE, score for neonatal acute physiology—perinatal extension.

^a^ Race and ethnicity data were collected via self-report and included to characterize the patient population for generalizability of findings.

^b^ Other racial or ethnic group included American Indian or Alaska Native and more than 1 race reported. These groups were consolidated owing to small numbers.

^c^ Defined as born at a center participating in the Eunice Kennedy Shriver National Institute of Child Health and Human Development Neonatal Research Network.

^d^ One infant missing head circumference.

^e^ SNAPPE score not calculated for 6 infants.

^f^ Adjusted for clustering of hemoglobin values within infant and shown as mean (SE) with *P* < .001 between high- and low-threshold groups.

^g^ During transfusion event with near-infrared spectroscopy monitoring.

^h^ Hypocarbia detected during blood-gas assessment during a transfusion.

^i^ Includes dopamine, dobutamine, epinephrine, or milrinone use at time of transfusion.

**Figure 1.**
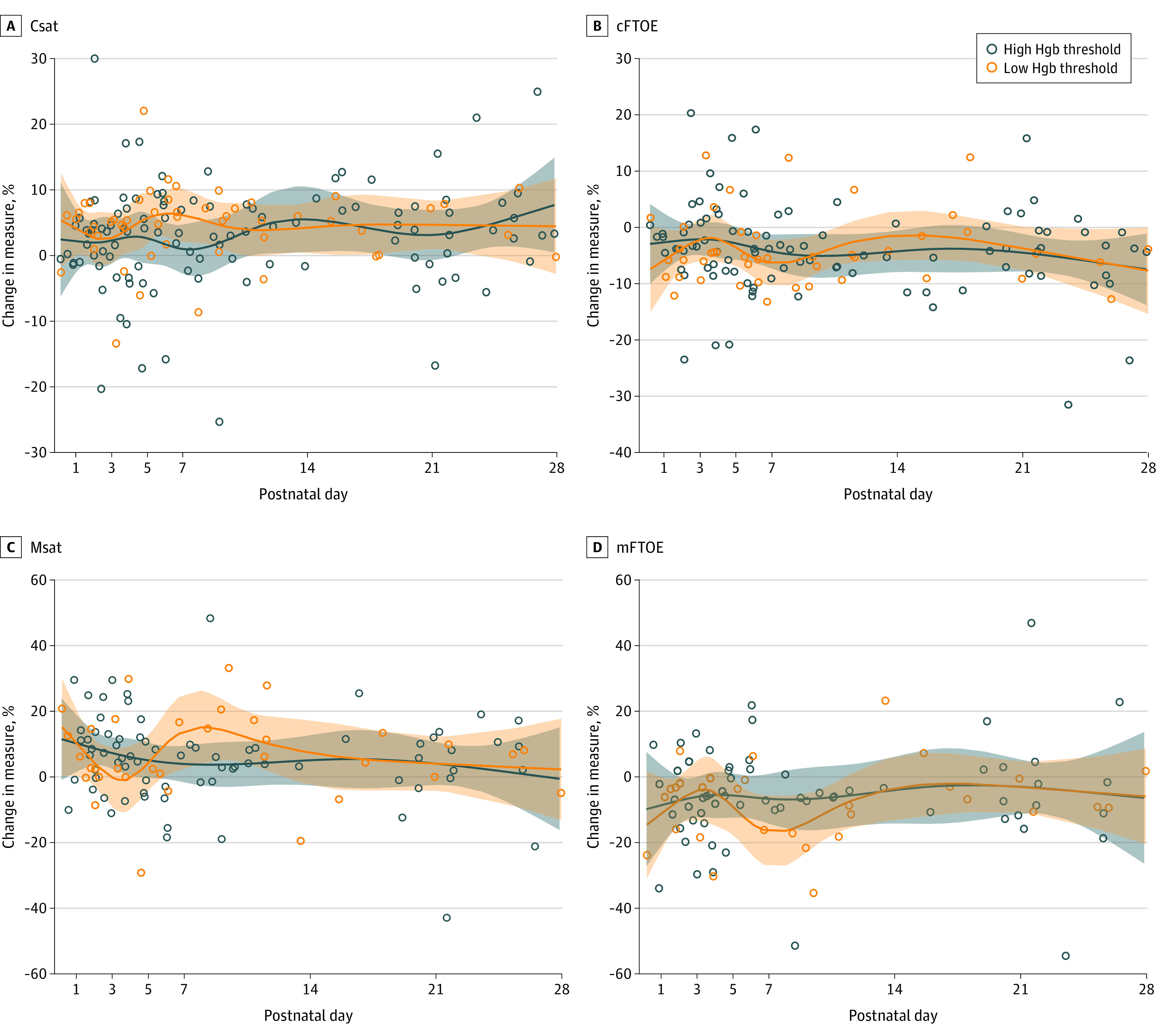
Change in Near-Infrared Spectroscopy (NIRS) Measures with Transfusion by Hemoglobin (Hgb) Threshold Group In the first 28 days after birth, cerebral saturation (Csat) and mesenteric saturation (Msat) increased after packed red blood cell transfusion, while cerebral fractional tissue oxygen extraction (cFTOE) and mesenteric fractional tissue oxygen extraction (mFTOE) decreased. However, changes in NIRS measures were not significantly different between the high– and low–Hgb threshold groups for Csat, cFTOE, Msat, or mFTOE.

**Figure 2.**
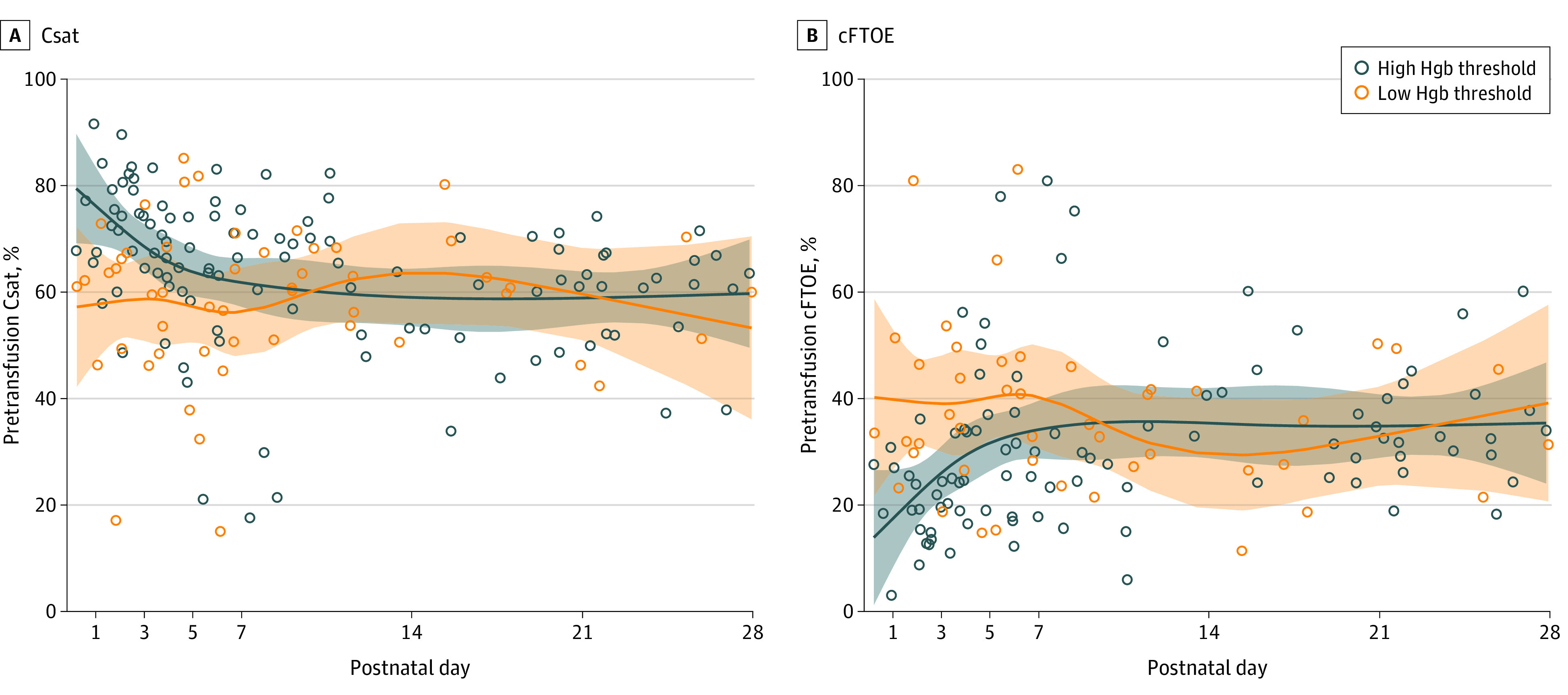
Pretransfusion Near-Infrared Spectroscopy (NIRS) Measures by Hemoglobin (Hgb) Threshold Group A, Pretransfusion cerebral saturation (Csat) in the first 28 days after birth in the high– compared to low–Hgb threshold groups. B, Pretransfusion cerebral fractional tissue oxygen extraction (cFTOE) in the first 28 days after birth in the high– compared to low–Hgb threshold groups.

Over the first 28 days, 80 infants had NIRS-monitored transfusions and were included in the primary analysis. Transfusion was significantly associated with an increase in both Csat and Msat, but with no significant difference between Hgb threshold groups (Figure 1). Mean Csat change in the lower–Hgb threshold group was 4.8% (95% CI, 2.7%-6.9%) compared to 2.7% (95% CI, 1.2%-4.2%) in the higher–Hgb threshold group, while mean change in Msat was 6.7% (95% CI, 2.4%-11.0%) vs 5.6% (95% CI, 2.7%-8.5%). There was no significant change in SpO_2_ within either group (0.2% vs −0.2%). Similarly, mean cFTOE decreased (4.2%; 95% CI, 1.8%-6.5%) in the lower–Hgb threshold group vs 3.6% (95% CI, 1.9%-5.3%) in the higher–Hgb threshold group), and mean mFTOE decreased (8.1% [95% CI, 2.8%-13.4%] vs 5.4% [95% CI, 1.8%-9.1%]) after transfusion but with no difference between Hgb threshold groups (Figure 1). Changes in NIRS measures based on chronologic age at transfusion were also not significantly different over the first 28 days. Figure 2 demonstrates that mean pretransfusion Csat was lower (60% [95% CI, 55%-64%] vs 63% [95% CI, 59%-67%)]; *P* = .11) and mean cFTOE higher (37% [95% CI, 32%-42%] vs 32% [95% CI, 28%-36%]; *P* = .08) in the lower–Hgb threshold group compared to the higher–Hgb threshold group. The trajectories of measures suggest decreased differences over the first 28 days, but these were not significant. Similar differences between Hgb threshold groups were also seen for pretransfusion Msat (34% [95% CI, 26%-41%] vs 43% [95% CI, 37%-48%]; *P* = .07) and mFTOE (64% [95% CI, 55%-72%] vs 52% [95% CI, 42%-58%]; *P* = .03).

**Figure 3.**
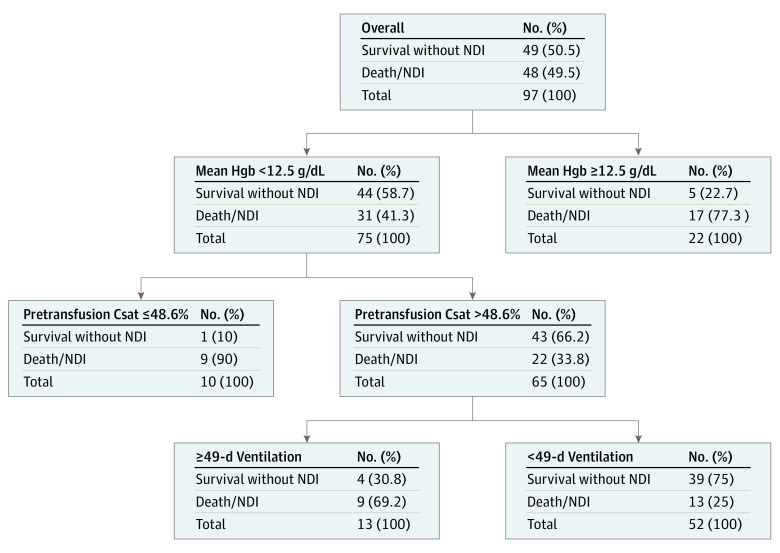
Factors Associated With Death or Neurodevelopmental Impairment (NDI) From Classification and Regression Tree Analysis Csat indicates pretransfusion cerebral saturation.

Data on the secondary composite outcome of NDI or death were available for 97 infants (96%; 4 infants were lost to follow-up). NDI or death occurred in 36 infants (37%); 29 (30%) survived with NDI and 7 (7%) died. Predictors of NDI or death ordered by importance in the CART analysis were infant mean pretransfusion Csat 48.6% or less, mean study Hgb 12.5 g/dL or greater, and 49 or more days of mechanical ventilation prior to hospital discharge. Residual sum of squares-based importance statistic was 2.34 for pretransfusion Csat and 2.10 for Hgb. Entropy was 0.78. Figure 3 depicts the pruned CART model with corresponding distribution of outcomes and *c* statistic of 0.78. The same variables offered to the CART procedure were entered into a stepwise logistic regression procedure for validation. Significant prognostic factors for NDI or death included the total number of transfusions with mean pretransfusion cerebral saturation less than 50% (odds ratio, 2.41; 95% CI, 1.08-5.41; *P* = .03), total days of mechanical ventilation, and mean study Hgb (Table 2), with a similar *c* statistic of 0.73.

**Table 2.** Stepwise Logistic Regression for Death or Neurodevelopmental Impairment

Stepwise variables added	Odds ratio (95% CI)	*P* value
No. of transfusions with mean pretransfusion Csat <50%	2.41 (1.08-5.41)	.03
Total days of ventilation	1.02 (1.00-1.04)	.03
Mean study Hgb	1.72 (0.98-3.03)	.06
Gestational age in wk^a^	1.1 (0.79-1.67)	.48
Male sex^a^	0.52 (0.19-1.40)	.19

Abbreviations: Csat, pretransfusion cerebral saturation; Hgb, hemoglobin.

^a^ Additional adjustment factors in the final model along with center as a random effect.

Post hoc exploration of predictors based on CART analysis was performed. Pretransfusion Csat less than 50% occurred in 15 infants. Six of these 15 were in the lower–Hgb threshold group for transfusion with mean (SD) Csat 38% (14%) while 9 were in the higher–Hgb threshold group with mean (SD) pretransfusion Csat 35% (13%). Mean study Hgb 12.5 g/dL or greater occurred in 22 infants, 17 of whom (77%) had the adverse outcome of NDI or death. These 22 infants did not have significantly different mean pretransfusion Csat compared to the 75 infants with Hgb less than 12.5 g/dL (mean [SD], 63% [19%] vs 61% [13%]). However, they were more likely to have received extra transfusions outside of study protocol (mean [SD], 9 [3] vs 2 [1]; *P* = .006) and had a greater number of Hgb tests in the first postnatal week compared to other infants (mean [SD], 7 [5] vs 4 [3]; *P* = .01).

## Discussion

This prospective secondary analysis of the TOP randomized clinical trial found that RBC transfusion was associated with an increase in regional oxygenation of both brain and mesenteric tissue despite no change in SpO_2_ in preterm infants with extremely low birth weight. At the time of transfusion, there were no differences in tissue oxygenation based on degree of anemia, as indicated by lower– vs higher–Hgb threshold group. In exploratory analyses, pretransfusion Csat less than 50% was found to be associated with NDI or death, supporting further investigation of targeted tissue saturation monitoring in infants with anemia.

Similar to several observational, single-center studies, we also found a significant increase in both Csat and Msat after transfusion and a decrease in fractional tissue oxygen extraction.^4,5,6,7,8^ Although mesenteric tissue is not as well autoregulated as the brain, Msat and Csat increased to a similar degree with transfusion. Without NIRS monitoring of cerebral and mesenteric tissue beds, the real-time effects of RBC transfusion may not be reflected in SpO_2_. Fredrickson et al^19^ also did not detect differences after transfusion in SpO_2_ or fraction of inspired oxygen between groups with liberal or restrictive transfusion thresholds.

Although we expected a greater increase in tissue saturation among infants with worse anemia, Csat and Msat increased to a similar extent in both the restrictive and liberal transfusion threshold groups, with corresponding decreases in cFTOE and mFTOE. Other investigators^9^ found that a liberal Hgb threshold policy for transfusion did not significantly increase Csat. However, in that study, the pretransfusion Csat levels were all within reassuring range (greater than 55%) compared to the population in our study, in which pretransfusion Csat was less than 50% even in some infants in the higher–Hgb threshold group (Figure 2). Infants in the lower–Hgb threshold group tended to have lower pretransfusion Csat compared to those in the higher–Hgb threshold group. However, this was not always the case, underscoring the potential role of other factors contributing to cerebral hypoxia, including systemic hypoxia, cardiac output, and hypocarbia. The trajectory of pretransfusion NIRS measures over time (Figure 2) is also consistent with literature describing decreased Csat and compensatory increased cFTOE in infants with worse anemia,^8,20,21,22^ but with diminished associations between NIRS measures and Hgb after repeated exposures to transfusions^21^ and with improved cardiac output.^23^ These findings support the idea that Hgb threshold may not be the best indicator of need for transfusion. As an adjunct to Hgb values, Csat or cFTOE could be considered for individualized determination of transfusion thresholds.

In pursuing our secondary, hypothesis-generating objective to explore the association of cerebral NIRS measures and other clinical factors with the adverse composite outcome of NDI or death,^24^ we used different analytical approaches to interrogate this complex question.^25^ As CART models accommodate a large number of predictor variables without assuming order of importance or preexisting cut points of variables, this statistical method was optimal for initial data exploration. Stepwise logistic regression provided independent validation of critical predictor variables. The complementary approaches of CART analysis and stepwise logistic regression both indicated a low pretransfusion Csat less than 48.6% (CART) and the total number of transfusions with a pretransfusion Csat less than 50% (logistic regression) as being associated with adverse outcome. Time spent below similar thresholds of Csat has been associated with adverse neurodevelopmental outcomes in other large NIRS monitoring studies of preterm infants,^26,27^ although the specific threshold of concern may vary based on whether an adult or neonatal sensor is used.^28^ Moreover, pretransfusion Csat was identified as a more important classifying variable than Hgb in the CART analysis; similarly, the number of transfusions with mean pretransfusion Csat less than 50% was more closely associated with NDI or death in the regression model than mean study Hgb. These findings, while exploratory, suggest that the NIRS measure of pretransfusion Csat may be a better indicator of death or NDI than Hgb threshold for transfusion and that severity of anemia should also be evaluated in the context of brain oxygenation.

The utility of Hgb as a measure of adverse outcome in the preterm population remains unclear. Notably, the Hgb threshold arm for transfusion was not found to be a significant variable for adverse outcome in either modeling process, echoing findings from the larger TOP trial.^2^ Although Hgb 12.5 mg/dL or greater was an associated variable from the CART analysis, infants with Hgb 12.5 mg/dL or greater received more transfusions outside of study protocol than other infants and may have intentionally been kept at a higher Hgb level due to severity of illness, inherently placing them at higher risk for adverse outcomes. However, the possibility that higher Hgb levels are detrimental in specific circumstances should also be considered.^29,30^

### Limitations

Interpretation of the findings requires attention to several study limitations. More advanced modeling to impute missing NIRS values at detection limits was not used.^31^ Other confounders may also influence NIRS measures over the course of a transfusion, including presence of a hemodynamically significant patent ductus arteriosus, blood pressure instability, carbon dioxide tension, significant hypoxia, sepsis, necrotizing enterocolitis, or intraventricular hemorrhage. While Table 1 shows no significant differences in respiratory support, pressor use, or significant hypocarbia between groups at the time of transfusion, more granular data were not available to explore additional associations. For example, in a prospective observational study, very low birth weight infants with an open patent ductus arteriosus had increased cerebral oxygenation but not splanchnic oxygenation 24 hours after transfusion.^32^ Another mechanistic consideration is that after a transfusion, a corresponding change in cardiac output or end-organ vascular resistance may impact NIRS measures beyond the effect of an increase in Hgb. Although sample size limits the significance of our findings, the population included in this secondary study reflected the larger TOP trial with similar NDI rates. Moreover, to our knowledge, this is the largest multicenter NIRS monitoring study in infants with extremely low birth weight exploring regional tissue oxygenation response to transfusion.

## Conclusion

In this study, tissue oxygenation of the brain and mesenteric region improved after transfusion, independent of Hgb threshold group. While Csat below 50% may be associated with adverse outcomes, future prospective investigation to compare current Hgb threshold-based transfusion practices to an individualized cerebral NIRS measure-based transfusion guideline is warranted. An improved precision medicine approach with cerebral oxygenation monitoring may facilitate a brain protective strategy for transfusion practices.

## Notes

Supplement 1.
Trial Protocol

Supplement 2. **eTable 1.** Hemoglobin Transfusion Thresholds **eTable 2.** CART Variables **eFigure.** Flow Diagram

Supplement 3.
Eunice Kennedy Shriver National Institute of Child Health and Human Development Neonatal Research Network Investigators

Supplement 4.
Data Sharing Statement
