# Supplementary material for: Tissue Oxygenation Changes After Transfusion and Outcomes in Preterm Infants: A Secondary Near-Infrared Spectroscopy Study of the Transfusion of Prematures Randomized Clinical Trial (TOP NIRS)
Source: JAMA Netw Open. 2023 Sep 21;6(9):e2334889. doi: 10.1001/jamanetworkopen.2023.34889 (PMC10514737; doi:10.1001/jamanetworkopen.2023.34889)
Supplement: Supplement 1. — Trial Protocol [file jamanetwopen-e2334889-s001.pdf]

Transfusion of Prematures (TOP) Trial:

Does a Liberal Red Blood Cell Transfusion Strategy Improve Neurologically-Intact Survival of Extremely-Low-Birth-Weight Infants as Compared to a Restrictive Strategy?

Haresh Kirpalani, BM, MSc [chair], Ed Bell, MD [v. chair], Carl, D'Angio, MD, Susan Hintz, MD, Kathleen Kennedy, MD, Robin Ohls, MD, Brenda Poindexter MD, MS, Kurt Schibler, MD, Barbara Schmidt, MD, Betty Vohr, MD, Jack Widness, MD, Abhik Das, PhD, Rose Higgins, MD, John Zupancic, MD, ScD, Robin Roberts, MSc Robin Whyte MD, MD, Aasma Chaudhary, BS, Karen Johnson, BSN

Version 5.0
Final: October 8, 2012
Revised: August 27, 2013
Revised: April 9, 2014
Revised: June 15, 2015
Revised: April 7, 2016

Table of Contents

Chapter **Page**

TABLE OF CONTENTS **II**

LIST OF TABLES AND FIGURES **IV**

1.0 SYNOPSIS **1**

2.0 BACKGROUND AND SIGNIFICANCE **2**

2.1 Increasing Survival of Newborn Preterms Less Than 1000 g BW 2
2.2 Poor Outcomes of Survivors Born at Less Than 1000 g BW 2
2.3 High Frequency of Blood Transfusions in Neonatal Units 2
2.4 Failure of Other Strategies to Prevent Allogeneic Red Blood Cell Transfusions 3
2.5 Contradictions of Data on Risks and Benefits of Transfusion from Observational Data – only weak evidence based recommendations 3

3.0 INNOVATION **6**

3.1 Research Design and Milieu 6
3.2 Economic Outcomes 6
3.3 Differing Blood Bank Practices 7
3.3.1 Red Blood Cell Storage Lesions and Age of Transfused RBCs 7
3.3.2 Storage Media for Red Blood Cells (RBCs) 7
3.3.3 Leukoreduction 8
3.3.4 Ensuring Cytomegalovirus-Negative Blood Products for Transfusion 8
3.3.5 Irradiation of Blood 8
3.3.6 How this Trial will Deal with These Possible Confounders 9

4.0 SPECIFIC AIMS **10**

4.1 Specific Aim 1 10
4.2 Specific Aim 2 10

5.0 APPROACH **12**

5.1 Preliminary Studies 12
5.1.1 Follow-up of Randomized Cohorts 13

6.0 STUDY DESIGN 15

6.1 Study Population, Inclusion and Exclusion Criteria, Withdrawals 15
6.2 Intervention Maneuver -- How We Developed the Algorithm Triggering Transfusions 15
6.3 Stratification, Random Allocation, and Enrollment Procedure 17
6.4 Prevention of Bias in an Unblinded Trial 17
6.5 Maintaining Adherence to Algorithm and Minimizing Deviation from Protocol 17
6.6 Choice of Primary Outcome 18
6.7 Main secondary Outcomes 19
6.8 Potential Confounders and Strategy to Limit their Impact 19
6.9 Statistical Design Considerations 20
6.10 Analysis Plan 20
6.11 Data and Information Management 21

7.0 METHODS 22

7.1 Randomization Method 22
7.2 Recruitment and Enrollment Plans 22

8.0 DATA SAFETY AND MONITORING 24

9.0 STUDY ORGANIZATION AND ADMINISTRATION 25

9.1 Study Sites 25
9.2 Regulatory Compliance and Monitoring 25
9.3 Recruitment Pace 25

10.0 ETHICS 26

10.1 Risk Category, Privacy 26
10.2 Consent and Oversight from NRN and RTI, and Interaction with Local IRBs 27
10.3 Involvement of Human Subjects 28

REFERENCES 29

APPENDIX A: DATA AND SAFETY MONITORING PLAN A-1

List of Tables and Figures

Table 1 Population Comparison between Trials 12
Table 2 Intervention Comparison between Trials 12
Table 3 Primary Outcomes 13
Table 4 Secondary Outcomes 13
Table 5 PINT-OS 18 to 24-Month Outcomes 13
Table 6 Transfusion Thresholds (Hemoglobin g/dl) 16
Table 7 Transfusion Thresholds (Hematocrit) 16
Table 8 Enrollment Timeline for the TOP Trial 22

Figure 1. PINT-OS Outcomes Using the *post-hoc* Outcome of MDI<85 (mean minus 1 SD) 14

1.0 Synopsis

Long-term outcomes of extremely low birth weight (ELBW) preterm infants, those weighing less than 1000 g at birth, are poor and pose a major health care burden. Virtually all of these infants are transfused, but at inconsistent hemoglobin (Hgb) thresholds. This results from conflicting data on when these infants should be transfused, preventing an evidence-based recommendation. However, the Prematures In Need of Transfusion (PINT) study[1] suggests that higher Hgb thresholds for transfusion may be beneficial to long-term neurocognitive outcomes at 18-22 months.[2] If a definitive adequately powered trial verifies this, the potential impact is high. An economic analysis of the PINT study outcomes showed that the increased costs of transfusion in the liberally transfused group would be more than offset by benefits of reduction in poor outcomes at 24 months.[3] On the other hand, the Iowa RCT showed that short-term potential neurological benefits of more liberal blood transfusion[4] were offset by poorer outcomes in brain structure and function at 7-10 year follow-up.[5,6] This study however had a significant attrition making it difficult to understand its implications.We propose in TOP to randomize infants ≤1000 g BW and < 29 weeks GA to receive red blood cell (RBC) transfusions according to one of two strategies of Hgb thresholds, either a high Hgb (liberal transfusion) or a low Hgb (restrictive transfusion) algorithm. It is currently unknown which transfusion strategy is superior. TOP is powered to demonstrate which strategy reduces the primary outcome of death or neurodisability in survivors at 22-26 months.

We therefore propose an open, parallel-group multicenter randomized controlled trial (RCT) analyzed by intention to treat. TOP randomizes extremely-low-birth-weight infants (birth weight less than or equal to 1000 g) and gestational age at least 22 weeks and <29 weeks) to either a liberal or a restricted red cell transfusion regimen according to hemoglobin thresholds. Following consent of eligible infants, central randomization will be performed within strata of birth weight (<750 g versus 750-1000 g) and study center, with variable block sizes. Compared to larger preterm infants, ELBW infants are at greater risk of death and neurodevelopmental sequelae. Two previous trials - the PINT trial [1] and its follow-up PINT-Outcome Study (PINT-OS) [2],, and the Iowa [4] trial and its follow up study [5] - raise important but contradictory implications for CNS injury and development. These can only be resolved in a new large trial. The objective of the TOP trial is to determine whether higher hemoglobin thresholds for transfusing ELBW infants resulting in higher hemoglobin levels lead to improvement in the primary outcome of survival and rates of neurodevelopmental impairment (NDI) at 22-26 months**.**

2.0
Background and Significance

2.1 Increasing Survival of Newborn Preterms Less Than 1000 g BW

Preterm birth at any gestational age is a serious and large burden for individual families, the health care system, and society. “In an average week in the United States: 10,056 babies are born preterm (less than 37 completed weeks of pregnancy); 1,604 babies are born very preterm (less than 32 completed weeks); 6,511 babies are born with a low birth weight (less than 2500 grams birth weight); 1,188 babies are born at very low birth weight” (less than 1500 g BW) .[7] The societal economic cost (medical, educational and lost productivity) is estimated for 2005 as in excess of $26 billion.[8] Most of the health burden stems from the most vulnerable infants in this group, those <1000 g BW, termed extremely-low-birth-weight (ELBW) infants.

2.2 Poor Outcomes of Survivors Born at Less Than 1000 g BW

As survival has improved in ELBW infants, focus increases on the degree of how ‘intact’ the survival is. Intact survival is an end-point that is meaningful to parents of surviving children, society overall, and health care providers. Recent ELBW outcomes are not reassuring. Among infants born at less than 25 weeks and <1000 grams in 2002-2004 in the NICHD Neonatal Research Network, 51% of survivors had BSID-II Mental Development Index (MDI) scores <70 at 18-22 months corrected age.[9] Survivors at school age have even more concerning outcomes. For example, ELBW survivors at 6 years of age were approximately 40% more likely than matched classmates born at term to have cognitive delay, defined as >2 SD below test mean scores.[10] Follow-up to adulthood adds concerns of a lack of age-appropriate sociability, which ranges from potentially beneficial effects of less delinquent behavior [11] to more worrying effects including infertility.[12] It is true that a small study of selected adults born with ELBW show high self-perceived health-related quality of life scores.[13] However, this may reflect an accommodation and adjustment to a lower functional ability.[14,15]

2.3 High Frequency of Blood Transfusions in Neonatal Units

ELBW infants all become anemic in early life, and approximately 90% receive one or more blood transfusions.[16] Preterm infants require transfusions for several reasons. Relative to term infants, preterm infants at birth have lower hemoglobin levels.[17] Repeated iatrogenic losses for laboratory sampling exacerbate the problem[18,19] and reflect prolonged intensive care. The anemia of prematurity is characterized by a reduced red cell life span, low levels of endogenous erythropoietin and a hyporegenerative bone marrow. This results in a universal and generally severe anemia in this population. The most powerful predictors of transfusion are low gestational age, low initial hemoglobin, large volume of iatrogenic loss, and low endogenous erythropoietin.[20] In the 1980’s reported rates of transfusion were 80 to 90% for infants <1.5 kg BW, and 100% for infants <1000 g BW.[21] However, transfusion practices are changing.[22,23] Comparing 1982 and 1993 transfusion data[21], found that the number of transfusions per infant dropped significantly (from 7.0 ± 7.4 to 2.3 ± 2.7). This decline was associated with a decrease in pre-transfusion hematocrit (33.6 +/- 2.8% in 1982, 29.8 +/- 5.1% in 1993). In the past decade, RBC transfusion rates remained high in all studies. From 2000 to 2005 in the University of Iowa’s NICU, the mean (±SD) of RBC transfusions given to ELBW infant’s prior discharge was 5.4 ± 0.9 (unpublished).

2.4 Failure of Other Strategies to Prevent Allogeneic Red Blood Cell Transfusions

Unfortunately, strategies to limit blood transfusions have had only limited success. Several methods have been proposed to "keep the blood in the baby".[24,25] Average phlebotomy losses are high, from 40 to 80 ml/kg.[26,27] Spinning down blood samples to re-infuse red cells, is logistically awkward and time consuming.[28] "Point-of-care" testing in an RCT[29] did not reduce transfusion. Initial enthusiasm for aggressive human recombinant erythropoietin to boost bone marrow RBC production has waned. A systematic review by Vamvakas and Strauss[30] found only modest effects of erythropoietin on transfusion need. In infants, a Cochrane review suggests that erythropoietin may elevate the risk of retinopathy.[31] A large, well designed trial found erythropoietin therapy did not significantly reduce transfusions in infants and conferred no benefits in survival, chronic lung disease, retinopathy of prematurity or intraventricular hemorrhage.[32] On follow-up to 22 months, these infants had no benefit in neurodevelopmental outcome[33], except in a small highly selected *post-hoc* subset.[34] In the TOP trial, most participating sites do not use erythropoietin except for children of parents with religious objections to blood transfusion. Increasing the red cell mass by delaying cord clamping may also boost blood volume, and seven trials in preterm infants are summarized in the Cochrane review.[35] With delay in cord clamping of up to 120 seconds, reductions were seen in transfusions (3 trials, 111 infants; relative risk (RR) 2.01, 95% CI 1.24, 3.27); and hypotension (2 trials, 58 infants; RR 2.58, 95% CI 1.17, 5.67); and intraventricular hemorrhage (five trials, 225 infants; RR 1.74, 95% CI 1.08, 2.81). Two later systematic reviews find a total of fifteen studies enrolling in total 734 preterms <37 weeks.[36,37] Unfortunately, few infants below 1000 g have been enrolled in RCTs of either delayed cord clamping or cord milking. A meta-analysis by this group found only a total of 63 infants below 1000 g. [38]

2.5 Contradictions of Data on Risks and Benefits of Transfusion from Observational Data – only weak evidence based recommendations

Blood product use has risen sharply over recent years, but sound indications for use are lacking.[39] Whether higher or lower transfusion thresholds are optimal in preterm infants remains controversial. Summarizing the data, a Cochrane review of infant studies shows no clear advantage of higher or lower hemoglobin transfusion thresholds for preterm infants and justifies clinical and scientific equipoise.[40] Historically, both higher and lower transfusion thresholds were proposed on physiological grounds. But data have been contradictory, resulting in variable practices. This is still the case as shown by an international survey conducted by the PIs – where neonatologists chose to transfuse a sample infant over a very large range of hemoglobin or hematocrit.[41] The controversy arises because of the dearth of high quality data, impeding the development of robust evidence-based guidelines. Many NICUs base their transfusion guidelines on those used in a 1995 trial of recombinant human erythropoietin to reduce transfusions.[42] Trials show that strict criteria can reduce transfusions.[43] However number of transfusions may not be the appropriate end point to study.

In the absence of firm evidence, guidelines for transfusion threshold have trended downward, including those of the Fetus and Newborn Committee of the Canadian Paediatric Society.[44] Current reports advocating lower transfusion thresholds for preterm infants are predominantly based on observational or retrospective study designs.[45] Authors of such reports often cite adult trials, such as the TRIC trial.[46] But extrapolating from these trials to the infant population carries obvious serious limitations. One biological challenge is that the preterm brain (in contrast to the adult brain) is developing and may have greater potential for damage from hypoxemia. Moreover, the adult data have had methodological limitations. In particular, the major cited finding of the TRICC trial was that younger and less sick patients were at higher risk of mortality. [46] However, this was a subgroup analysis not confirmed in a systematic review of

multiple studies.[47] Contradictory findings have prompted calls for a new large adult randomized trial.[48]

We review here the arguments for lower and higher transfusion thresholds. First, data arguing for lower thresholds are discussed. A retrospective study in Brazil found an association between blood transfusions and neonatal mortality.[49] The authors found an increased relative risk of death of 1.49 (1.17-1.78) in infants who received at least one RBC transfusion compared with non-transfused infants. However, methodological difficulties include confounding analyses in such a data set – sick infants are more likely to be transfused. Moreover, in the largest neonatal RCT of high versus low hemoglobin threshold, the RR favored higher thresholds, but this effect was not statistically significant.[1] These methodological issues are discussed in a commentary editorial on dos Santos.[50] Frequent transfusions can result in iron overload, which was found at autopsy to correlate with number of transfusions given to preterm infants.[51] Excess tissue iron acts as an oxidizing agent. This has implications for some of the major and serious complications affecting ELBW infants, namely ROP, BPD and NEC.[52,53,54,55,56] Several recent studies report increased risk of NEC following blood transfusions.[57,58,59,60,61,62,63] One such report found that blood from male donors was more likely to be associated with NEC.[64] Unfortunately, these are mainly retrospective observations, and all demonstrate, at best, association and not causality.

Finally, liberal transfusion policies must consider infection risks. Assessing the risk-benefit ratio of transfusion requires consideration of the unknown but real potential for transmission of infective agents that are undetected or even unknown.[65] With new standards of screening for the HIV virus, this agent is less prominent. However, other blood-borne agents previously not considered as potential vectors (including babesiosis and Creutzfeldt-Jakob disease variant viruses) have recently been implicated in transmission of infection.[66,67] Cytomegalovirus (CMV) is still a potential concern despite efforts to mitigate its transmission in donated blood products.[68] Despite leukoreduction of donor blood or the use of CMV-negative donors, rates of CMV break-through infection continue at 1-3% of transfused subjects.[69] At current low rates of transmission, we acknowledge that our proposal is unlikely to be large enough to address these concerns directly.

Those who favor higher transfusion thresholds, on the other hand, cite evidence that transfusing at higher hemoglobin thresholds reduces apnea of prematurity and fosters weight gain[70] , increases oxygenation in ventilated infants[71], and reduces compensatory increases in cardiac output.[72,73] However, apnea is reduced by simple volume infusion as well as RBC transfusion.[74] Moreover, the two RCTs that examined the impact of transfusion threshold on apnea differed in their findings: the Iowa trial found less frequent apnea with higher transfusion thresholds[4], whereas the PINT study found no difference.[1] The beneficial impact of higher hemoglobin thresholds on weight gain was not supported by PINT or other studies.[75] Improved echocardiographic findings of cardiac parameters with higher hemoglobins[71] may be simply surrogate physiological adaptations. Claims of increases in general body oxygenation are also challenged by measures of near infrared spectroscopy in peripheral forearm tissue.[76,77] There was too large an overlap between symptomatic and asymptomatic infants, which precluded distinguishing on the basis of peripheral oxygen extraction.[78] Nonetheless, the same technology for assessing brain oxygen saturation appears to confirm that infants with lower hemoglobins tend to have lower regional brain oxygen saturation.[79,80,81] This may be relevant to the observation that lower rates of IVH were associated with higher hemoglobins.[82,83] Even those who advocate lower hemoglobin ranges for transfusion often recommend higher hemoglobin thresholds in the first week of life in the hope that this will help to protect against brain hemorrhage.[45] However, once again, the data are contradictory. A large observational study of

417 newborns found that a simple grade I germinal matrix hemorrhage was more likely to evolve into a grade 3 or 4 IVH if there had been a preceding RBC transfusion (OR 2.92; 95% CI 2.19, 3.90).[84] If it is true that higher hemoglobins confer neuroprotection from IVH, this implies a physiological mechanism to explain the observation in PINT-OS of better developmental outcome at higher hemoglobin thresholds.[2]

To conclude: The contradictory findings of previous studies make evidence-based recommendations nearly impossible. The TOP trial would produce substantial evidence on which to base recommendations to guide transfusion practice for ELBW infants.

3.0
Innovation

Summary: Many neonatologists have shifted toward using lower hemoglobin thresholds for transfusion, based largely on retrospective data. For example Valieva et al stated: “In our study of all ELBW infants treated at UW NICU during 2006, we did not identify any clinical benefits of transfusing PRBCs with the guidelines in place at the time. As a result of this study, the transfusion guidelines at the UWMC NICU were made more restrictive“.[45] In contrast, the TOP study proposes a higher-level study design, namely a randomized controlled trial. In addition, the need to assess long-term outcomes of randomized trials is compelling in the newborn, where developmental outcomes are of paramount interest to both parents and society.[85] A randomized comparison will enable comparison of health care costs of the two strategies in weighing competing risks and benefits. Our group has analyzed the short and long-term costs of the two transfusion strategies used in the PINT study.[3] Finally, in designing the current study, we have designed a strategy that accounts for differing blood bank practices and differences in dispensed products across multiple study sites. An ideal solution would be to establish tight guidelines for blood bank practices, but this would be difficult to implement and would limit the generalizability of the results. Instead, we will first conduct a study to survey individual blood bank practices and assess the correlation of hematocrit with volume and weight of dispensed cells. This will allow an *a-priori* adjustment for potential variation in hematocrit by dispensed RBCs by site.

3.1 Research Design and Milieu

This prospective randomized unmasked Phase III trial defines a clinically significant long-term outcome that will be assessed by examiners blinded to group assignment. It is led by experienced clinical trialists and biostatisticians who are familiar with the relevant methodological issues. Three of the four key investigators for the two paired applications (HK, EB, DB) have had specific experience with RCTs comparing transfusion strategies. The two major trials pertinent to this study question were designed and performed independently by the two clinical PIs. They were published within 6 months of each other. In reality, TOP is the result of a new partnership created by merging the two rival groups who had conducted the Iowa and PINT trials. These two studies, taken together, resulted in new hypotheses about the impact of hemoglobin level (or hematocrit) on the developing brain. These form the basis for the proposed TOP trial, which will be built on the very experienced, productive framework of the NICHD Neonatal Research Network.

3.2 Economic Outcomes

We will conduct a formal, prospective economic evaluation with patient-level cost and efficacy data alongside the parent TOP randomized controlled trial, using standard accepted methodology[86], as previously demonstrated by our group in similar multi-center trials.[87,88,89,90,91] The analysis will take a comprehensive societal perspective and a similar 24-month timeline to the parent trial. Direct medical resource utilization during the initial birth hospitalization will be ascertained through collection of itemized billing records and UB-04 forms, a uniform billing statement recommended by the National Uniform Billing Committee and utilized for reporting of hospital expenditures by all third party payers including the Centers for Medicare and Medicaid Services (CMS). We will convert hospital-reported charges to costs by applying the appropriate Center for Medicare and Medicaid Services cost-center specific ratio of costs to charges.[92,93,94] Physician professional fees for the initial hospitalization will be based on CMS reimbursement

levels for each day of stay and non-bundled procedure, using information in the TOP case report forms to assign a level of illness acuity and a billing code according to the Resource-Based Relative Value Scale.[95,96] Family out-of-pocket expenses and work-related productivity losses, as well as post-discharge health-care utilization will be ascertained by questionnaire administered during the initial hospitalization and at follow-up visit.

Sample size will be based on power considerations for the parent clinical trial. All analyses will utilize an intention-to-treat approach. Data confirmed to be missing at random will be addressed as appropriate using multiple imputation.[97] We will first directly compare mean cost between the study groups using generalized linear modeling to account for the typically right skewed cost data distribution.[98,99] We will then calculate the incremental cost-effectiveness ratio, defined as the difference in mean cost per patient in the high and low transfusion threshold arms divided by the difference in the mean effect between the study arms. The outcome will thus be expressed as the cost per survivor without neurodevelopmental impairment. To assess statistical uncertainty in the joint distribution of costs and effects, we will use nonparametric bootstrapping and report results as cost-effectiveness acceptability curves.[100,101,102,103] Finally, we will assess uncertainty in parameter values by using sensitivity analysis, in which we will recalculate the cost-effectiveness after varying the input values for certain variables through a plausible range.

3.3 Differing Blood Bank Practices

After collection, donor blood must be safeguarded against red cell hemolysis and infection. However, donor blood is stored with differing anticoagulants and varying conditions and for varying periods of time in different centers. Some such factors could act as co-interventions. A recent multinational study "confirmed the great variability in the hemoglobin levels in the RBCs transfused to patients".[104] However this is a generalizable and pragmatic trial. We will record these differences, but not attempt to control for them. *A-priori* secondary analyses will take account of these factors.

3.3.1 Red Blood Cell Storage Lesions and Age of Transfused RBCs

In the U.S., the Food and Drug Administration allows RBCs to be refrigerated for up to 42 days .[105] Stored blood is favored over fresh blood because it lowers administrative and collection costs, minimizes waste, and assures an adequate and readily available supply of blood. Although longer storage might be detrimental[106,107], human data are conflicting as to the benefits of "newer" (fresher) blood. In adults undergoing coronary artery bypass surgery, receipt of blood stored <14 days, was associated with improvement in all measured outcomes compared with those receiving older blood, including in-hospital mortality.[108] However, this retrospective study had an imbalance of massively transfused patients between the groups: more than half of the patients who died had received more than 6 units and were disproportionately more likely to have received older blood.[106] The question of the safety and efficacy of fresh versus stored blood is still open. An ongoing adult randomized trial[109]and a trial in preterm infants (Clinicaltrials.gov NCT00326924: The Age of Red Blood Cells in Premature Infants Study (ARIPI) are currently examining optimal length of RBC storage, with regard to clinically relevant outcomes including mortality.

3.3.2 Storage Media for Red Blood Cells (RBCs)

RBCs are stored so as to remain sterile, metabolically active (usually mandating dextrose solution) and intact, with specified targets for cell survival such that acute hemolysis of transfused RBCs is less than 1% and 24-hour survival of the RBCs in the recipient is at least

75%.[110] Anticoagulation is achieved with citrate, and phosphate is used as a buffer. Thus, most solutions contain citrate, phosphate, and dextrose (CPD). Additions of mannitol and adenine are intended to stabalize the RBC membrane and maintain 2,3-diphosphoglycerate and ATP levels within the RBC. The use of adenine has boosted the storage life of RBCs from 21 days for CPD to 35 days for citrate-phosphate-dextrose-adenine (CPDA-1), and up to 42 days with even newer formulations (Adsol, Optisol, and Nutricell). Reports suggest that prolonged storage may result in metabolic abnormalities.[111] Washing RBCs may limit exposure of the heavily transfused neonate to these storage compounds. There is inadequate information on the safety of these compounds, and the efficacy of washing RBCs is also unknown. Based on the quantities of anticoagulant preservatives solutions, during large volume transfusions to neonates, metabolic abnormalities are anticipated and documented in a few small studies.[111]

3.3.3 Leukoreduction

RBC transfusion may down-regulate immune status, a phenomenon termed transfusion-related immunomodulation or TRIM.[112,113,30] White cells (WBCs) in donor blood may be the source of both cytokines and histamine mediators as they deteriorate in storage, which may result in detrimental effects. Randomized trials have compared white cell reduction by buffy coat depletion against more targeted specific WBC reduction. Benefits of aggressive leukoreduction may include reducing hospital-acquired infections.[114,115] Other trials suggest leukoreduction may decrease absolute total postsurgical mortality by 4.2%.[116] In 1999, the Canadian national blood service adopted universal leukoreduction. Following this, a large retrospective study examined mortality effects. Both unadjusted and adjusted absolute hospital mortality rates were approximately 1% lower with leukoreduction (adjusted OR, 0.87; 95% CI, 0.75, 0.99; P=0.04).[117] A study of leukoreduction in premature infants found no change in the primary outcome of infection, but on secondary analysis, the composite rates of BPD, IVH, ROP and NEC were significantly reduced (OR 0.31 (95% CI, 0.17, 0.56).[118] Nonetheless, methodological problems preclude firm recommendations. Meta-analysis of trials in oncology supports skepticism about accepting these findings.[119,112,120]

3.3.4 Ensuring Cytomegalovirus-Negative Blood Products for Transfusion

Newborns are vulnerable to transfusion acquired cytomegalovirus (CMV). CMV is frequently present in asymptomatic adults, with some 70% of the population being seropositive.[121,122] Seropositive donors present a risk of transmission, even when not actively viremic. Some consider that CMV-seronegative blood should be given to preterm infants at risk of frequent transfusion. [121] However, this complicates testing the blood supply and assuring an adequate stock. Since the virus is thought to be latent in neutrophils, alternatives include leukocyte reduction or storage methods to reduce viral survival such as freezing in glycerol. Finally, 3rd-generation leukocyte-depletion filters prevent primary CMV in neonates[123] and in immunologically deficient adult bone-marrow transplant patients. However, there is no consensus on how best to prevent transfusion-acquired CMV infection.

3.3.5 Irradiation of Blood

Irradiation of blood does not prevent CMV transmission from RBC transfusion, as irradiation affects primarily lymphocytes. Thus, irradiation of blood is a center-specific measure with unproven benefit in reducing risk of CMV transmission to transfused newborns.[124] The relevance for newborns is unclear since the risk of graft versus host disease is unknown in ELBW infants.

3.3.6 How this Trial will Deal with These Possible Confounders

We will randomize 1824 infants and examine the primary outcome of death or neurodevelopmental impairment at 22-26 months. Given our large, generalizable, pragmatic trial design, we cannot mandate local laboratory practices to adopt uniform blood banking practices. Instead we have adopted the following strategy:

i. We have appointed a ‘TOP Trial Blood Banking Committee’ led by Dr. Naomi Luban as Chair Members were selected for expertise in blood banking practices. They will conduct a survey of all the blood banks by site to ensure that practices fall within an acceptable range for standards of care. Before the study begins, we will conduct a survey on pertinent blood bank practices in the 18 participating sites of the NRN. This will be the subject of a separate report and will inform the TOP trial of the likely magnitude of variation among the sites.

ii. Patients will be stratified by center, and analyses will adjust for center.

4.0
Specific Aims

4.1 Specific Aim 1

To examine whether the clinically relevant composite primary outcome of death or significant neurodevelopmental impairment in survivors at 22-26 months of corrected age is less common among preterm infants who, by transfusion practice, are maintained at higher hemoglobins. Neurodevelopmental assessments will be performed by trained and annually recertified examiners, who are masked to treatment allocation, using standardized tools: the Bayley Scales of Infant Development-III (BSID-III) and the Gross Motor Function Classification Scale (GMFCS).[125,126] The primary outcome consists of any one of the following: (i) *Cognitive delay* where delay is defined as BSID-III cognitive score < 85 (>1 standard deviation below the mean). (ii) *Cerebral Palsy* if the child has a non-progressive motor impairment characterized by abnormal muscle tone and impaired range or control of movements. Moderate cerebral palsy (CP) is defined as level II or III on the GMFCS. Severe or non-ambulatory CP consists of GMFCS IV or V. (iii) *Severe vision impairment,* defined as corrected visual acuity in the better eye less than 20/200, determined by pediatric ophthalmologists. (iv) *Severe hearing impairment*, defined as bilateral hearing loss requiring amplification or cochlear implant. (v) *Death* before 22-26 month follow-up assessment. The primary outcome is deemed present if one or more of the individual components of the composite outcome are present, or absent if no component was present. If no component is present, one or more missing components render the primary outcome to be deemed missing.

4.2 Specific Aim 2

We will examine in which arm the following secondary outcomes are more common:

Short term, to NICU discharge:

(a) Survival to discharge without severe morbidity, defined as any of the following: bronchopulmonary dysplasia, retinopathy of prematurity (stage ≥3 or requiring treatment), or serious brain abnormality (grade 3 or 4 intraventricular hemorrhage, periventricular leukomalacia, or ventriculomegaly).
(b) Weight, length, and head circumference at 36 weeks postmenstrual age or at discharge from NICU, whichever occurs first.
(c) Serious abnormality on cranial ultrasound examination: grade 3 or 4 intraventricular hemorrhage, periventricular leukomalacia, or ventriculomegaly;
(d) Age at final tracheal extubation; age at final caffeine dose
(e) Number of transfusions, numbers of donor exposures by RBC donors or other blood product
(g) For survivors, length of hospital stay in the level 3 NICU referral site, or in the level 3 area of the referral site;
(h) Episodes of necrotizing enterocolitis of Bell stage 2 or higher, and time to full feeds;

Long term (survivors only), at 22-26 months corrected age:

(i) The incidence of ambulatory and non-ambulatory CP defined by GMFCS;
(j) Hydrocephalus shunt, microcephaly, or seizure disorder;

(k) The presence of respiratory disease necessitating readmission before 22-26 months follow-up.
(l) All individual components of the composite outcome of NDI or death, including cognitive outcomes at follow-up at 1 SD cut-off on the BSID III standardized scales
(m) BSID III cognitive, language and motor scores at 2 SD cut-offs (<70) at follow-up.
(n) Economic cost-benefit analysis to time of discharge and to 22-26 month follow-up.

Potential Impact: This trial is powered to address an important unresolved clinical problem. Current trials have shown the hypothesis to be reasonable, and needs a definitive trial to prove or disprove. This trial will change clinical practice, whether or not the higher arm improves NDI. If it does improve, important further research should target how neurological pathways are improved by transfusion.

5.0
Approach

This trial directly builds on and benefits from the two major prior trials – led by the PIs of the TOP study - and their follow-up studies.[1,2,4,5,6]

5.1 Preliminary Studies

Historical trials are of limited help in today's clinical environment of infants <1000 g.[127,128] In the ELBW population, four trials are relevant. One trial examined differing thresholds for transfusion but with the adjunctive therapy of erythropoietin, and no significant differences were found.[129] One trial is too small (n=36) to be really informative[130], and its inclusion in the Cochrane review on neonatal transfusions did not alter the conclusions.[2] The Iowa and PINT trials are directly comparable, and erythropoietin was used in neither study.[1,4] These trials asked: "In high-risk preterm infants, does a low or a high hemoglobin or hematocrit transfusion threshold strategy lead to a clinically detectable benefit?" Subjects in both trials were high risk, but the birth weight criterion for entry was lower in PINT trial (Table 1). PINT ran in Canada, U.S. and Australia.

The intervention algorithm for transfusions also varied, but both trials adopted the principle of higher target hemoglobins for infants requiring oxygen and or respiratory support (Table 2). The main difference was in the hemoglobin separation between the restrictive and liberal groups, which was 2.7 g/dl in the Iowa trial as opposed to 1.1 g/dl in PINT. The transfusion thresholds were arrived at in differing ways. The Iowa group had extrapolated above and below current clinical ranges, and the PINT group stayed within the confines of recommendations of the Fetus and Newborn Committee of Canada[131] – to which participating units adhered.

Table 1 Population Comparison between Trials

	Iowa Trial		PINT Trial	
	Restrictive	Liberal	Restrictive	Liberal
Participating centers	1		10	
No. of subjects	100		451	
Treatment allocation	Randomized		Randomized	
Stratification	Birth weight		Birth weight, center	
Mean BW (g)	954	958	771	769
Mean GA (wk)	28	28	26	26

Table 2 Intervention Comparison between Trials

	Iowa Trial		PINT Trial	
	Restrictive	Liberal	Restrictive	Liberal
Transfusion thresholds				
Hemoglobin, g/dl				
Highest	11.3	15.3	11.5	13.5
Lowest	7.3	10.0	7.5	8.5
Mean hemoglobin	8.3	11.0	10.1	11.2
Mean hemoglobin difference	2.7		1.1	

The primary outcomes selected were different (Table 3). Iowa used a reduction in the number of

transfusions, which was significantly different. PINT constructed a composite outcome of death or any one of: BPD, ROP stage 3 or higher, or brain Injury (PVL, IVH grade 4, or ventriculomegaly). The primary outcome in PINT was not significantly different between the restrictive and liberal transfusion groups. The PINT trial also showed a reduction in transfusions. In an unplanned secondary analysis, the Iowa trial found excess severe IVH and PVL in the restrictive group (Table 4).

Table 3 Primary Outcomes

	Iowa Trial		PINT Trial	
Primary outcome	Number of transfusions		Composite outcome of death or any of the following: BPD, ROP stage ≥3, or brain injury (PVL, IVH grade 4, or ventriculomegaly)	
Result	Restrictive	Liberal	Restrictive	Liberal
	3.3	5.2	74%	70%
	P=0.025		Not significant	

Table 4 Secondary Outcomes

	Iowa Trial		PINT Trial	
	Restrictive	Liberal	Restrictive	Liberal
IVH grade 3 or 4	10%	16%		
PVL	14%*	0	2.7%	2.3%
PVL or IVH grade 4	12%	0		
Death	4%	2%	21.5%	17.5%

* Of those with late head ultrasound examinations

5.1.1 Follow-up of Randomized Cohorts

The PINT study reported follow-up of survivors at 18-24 months age, in the outcome study - PINT-OS.[2] The *a-priori* primary outcome was a composite of death or survival with one or more neurodevelopmental impairments: cognitive delay (Bayley II MDI <70); cerebral palsy; blindness; or deafness requiring amplification. The follow-up rate was 93%. For the primary outcome, the OR for low vs high transfusion thresholds was 1.45 (95% CI 0.94, 2.21) (p=0.091). The rate of death or impairment was 45.2 % in the low group vs 38.5% in the high group. However, in a secondary *a-priori* outcome assessment, the component parts of the primary outcome cluster were examined separately (Table 5).

Table 5 PINT-OS 18 to 24-Month Outcomes

	Odds Ratio	95% CI
Death	1.18	0.72,1.93
Cerebral Palsy	1.32	0.53, 3.27
Cognitive delay, Bayley MDI <70 (2 SD below mean)	1.74	0.98, 3.11
Blindness	2.16	0.19, 24.1
Deafness	1.45	0.32,6.58

All point estimates favored the high threshold group, but none was statistically significant. However, the nearly significant difference in the risk of cognitive delay, strongly

suggested a true effect of statistical significance was possible. We therefore conducted a *post-hoc* analysis using Bayley MDI score <85 (1 SD below the mean) to determine prevalence of cognitive impairment. This result is summarized below (Figure 1). As can be seen, the composite outcome is significant using the MDI cutoff of 85. The data do not allow firm conclusions, but a benefit to higher hemoglobin transfusion thresholds is plausible in reduced death (OR 1.38, 95% CI 0.84, 2.27) and/or reduced neurocognitive deficit in survivors. We believe these benefits of higher transfusion thresholds are sufficiently important to justify a new adequately powered study.

Figure 1. PINT-OS Outcomes Using the *post-hoc* Outcome of MDI<85 (mean minus 1 SD)

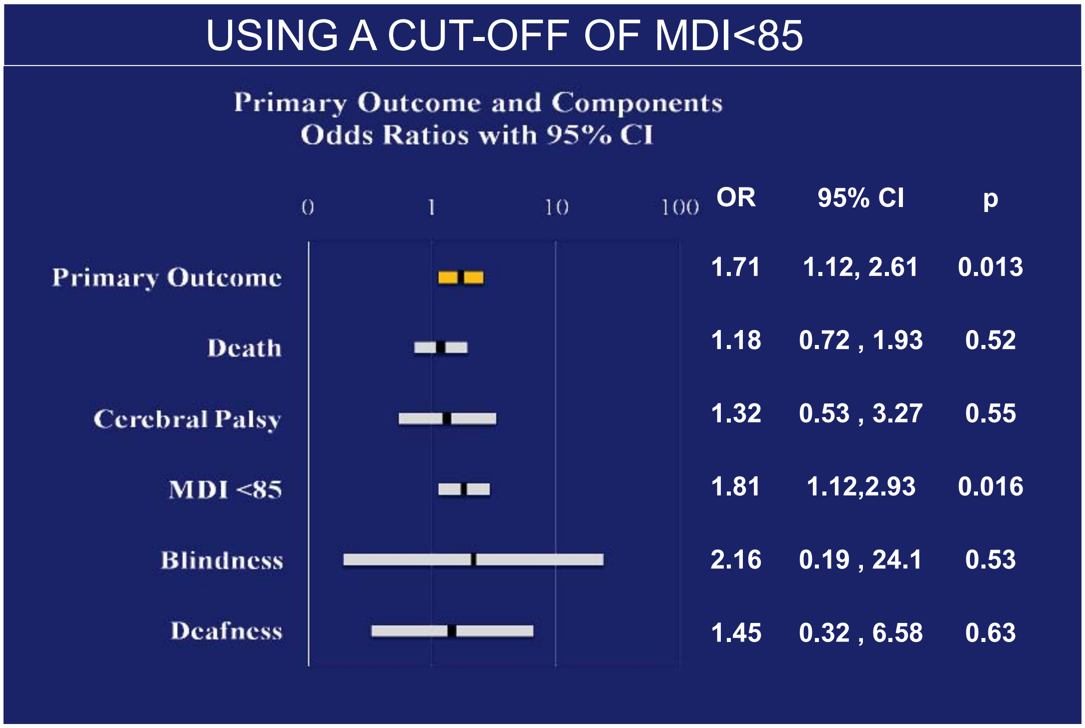

6.0
Study Design

6.1 Study Population, Inclusion and Exclusion Criteria, Withdrawals

Inclusion criteria: Infants – inborn or outborn – who are admitted to participating NICUs and who fulfill the following inclusion criteria are eligible for enrollment: (a) Birth weight ≤1000 g and gestational age at least 22 weeks but less than 29 weeks; and (b) Less than 48 hours of age.

Exclusion criteria: Infants meeting any of the following criteria will not be eligible: (a) Considered nonviable by the attending neonatologist. (b) Cyanotic congenital heart disease. (c) Parents opposed to the transfusion of blood. (d) Parents with hemoglobinopathy or congenital anemia. (e) *In-utero* fetal transfusion. (f) Twin-to-twin transfusion syndrome. (g) Isoimmune hemolytic disease. (h) Lack of parental consent. (i) Prior blood transfusion on clinical grounds beyond the first 6 hours of life. (j) High probability that the family is unable to return for the follow-up assessment at 22-26 months. (k) Infant has received erythropoietin prior to randomization, or is intended to receive erythropoietin through the neonatal course. (l) Congenital condition, other than premature birth, that adversely affects life expectancy or neurodevelopment.

Algorithm Deviations: Infants who by either late diagnosis or clinical deterioration are found to have conditions listed in the exclusions will be transfused according to the preference of their medical team rather than by study protocol. However, their outcomes will be measured and analyzed according to original allocation by intention-to-treat principles.

6.2 Intervention Maneuver -- How We Developed the Algorithm Triggering Transfusions

Previous trials using a titrated therapy were criticized as departing from standard practice.[132,133] Therefore, the ethically correct approach to define the Hgb thresholds is to remain within the range of current clinical standards of practice.[134,135] Threshold Hgbs used in practice vary from center to center, may depend on the neonatologist and are often unclear. We base our proposed transfusion thresholds on:

i. The range of hemoglobin thresholds used clinically to guide transfusion decisions in the participating NICUs of the NICHD Neonatal Research Network;
ii. A poll of the range of hemoglobin thresholds that would be acceptable to each neonatologist in an NRN site within the context of an RCT.

The low threshold values reflect more common practice, so this is considered the 'usual treatment' group. In this group, the transfusion thresholds are similar to those used for the restrictive group in both the PINT and Iowa trials. The highest threshold for the liberal transfusion group was the highest acceptable to neonatologists at the majority of NRN centers.

We identified two potential clinical modifiers of triggers to transfusion, postnatal age and respiratory support. The use of postnatal age to define transfusion thresholds is based on the physiologic fall in hemoglobin seen in infancy. Respiratory support is defined as mechanical ventilation, continuous positive airway pressure, F_iO_2 in excess of 0.35, or ~~oxygen~~ by nasal cannula ≥ 1 liter per min (please note: room air nasal cannula 1 lpm or greater is considered respiratory support). The final intervention algorithm reflects substantial dialogue with all NRN

site members. It will be applied to the enrolled population up to the postmenstrual age of 36 weeks or until the infant is discharged from the NICU, whichever occurs first.

The transfusion thresholds for the two groups are listed in Table 6 below, as hemoglobin in g/dl.

Table 6 Transfusion Thresholds (Hemoglobin g/dl)

	High threshold		Low threshold	
	Resp support	No support	Resp support	No support
Period:				
Week 1	13.0	12.0	11.0	10.0
Week 2	12.5	11.0	10.0	8.5
Weeks ≥3	11.0	10.0	8.5	7.0

The values given for Week 3 and later, will be used as transfusion thresholds until postmenstrual age 36 weeks or hospital discharge, whichever occurs first.

Since some NICUs prefer to use hematocrit, we provide the table by HCT also, using the conversion formula: Hematocrit (%) = 2.941 x Hgb (g/dl) (http://www.heartpumper.com/hematocrit.htm). – to provide the table below.

The transfusion thresholds for the two groups are listed in Table 7 below, as hematocrit.

Table 7 Transfusion Thresholds (Hematocrit)

	High threshold		Low threshold	
	Resp support	No support	Resp support	No support
Period:				
Week 1	38	35	32	29
Week 2	37	32	29	25
Weeks ≥3	32	29	25	21

Our proposed algorithm results in a difference in hemoglobin threshold of 2-3 g/dl in all age and respiratory categories. Moreover, these thresholds conform to the ranges within which all sites have confirmed equipoise.

Volumes of transfusions and hemoglobin measures: We will use a standard transfusion volume of 15 ml/kg, expecting to raise Hgb by about 4 g/dl. A fixed transfusion volume will yield higher compliance and a consistent difference in Hgb levels between the two arms. Centers are free to obtain blood samples for Hgb determination from venous, arterial, or prewarmed heel capillary blood according to vascular access and their usual practice. We recognize that values from large veins and arteries are systematically lower than capillary samples by about 8.5%.[17] However for a pragmatic trial, these small differences are not problematic and do not require adjustment. We will not mandate Hgb checks, but by polling we have ensured that most units obtain these at least weekly in the first month. We will record all Hgb values obtained.

Non-algorithm, or clinically driven transfusions: The algorithm will determine transfusion for routine or “top-up” transfusions, but additional transfusions may be given for infants with urgent need for transfusion because of bleeding or anticipated bleeding, e.g. during and after surgery. All blood transfusions will be recorded and categorized as “threshold reached” or “other indication” (*e.g.*, shock, severe sepsis with coagulopathy; or perioperative). All subsequent events will be analyzed by intention-to-treat principles, irrespective of additional transfusions, trial dropouts, or protocol violations.

Transfusions and blood banking: All RBCs transfused will be tested and screened according to the policies of the blood banks serving each hospital participating in the trial. These practices will be recorded throughout the trial, with notification to the DCC of any policy changes. The blood transfused will be ABO and Rh compatible with the infants’ type. Individual unit practices related to blood transfusion vary among the participating sites. Because randomization will be stratified by site, variation in local blood banking practices will not result in imbalances between treatment groups and will allow greater generalizability of the study results. The details of blood bank practice to be recorded for each site will include screening for CMV, storage buffer, maximum storage age, policies for RBC washing and leukoreduction, and methods of storing and concentrating RBCs. The age of donor blood will be recorded for each transfusion, but there is no restriction on age beyond individual site practice.

6.3 Stratification, Random Allocation, and Enrollment Procedure

Parents of eligible infants who conform to the inclusion and exclusion criteria will be approached by the clinical study team (either a research nurse coordinator or a physician investigator) within 48 hours of birth in order to offer study participation. A log will be maintained of all screened infants indicating which are eligible and which were enrolled. On receipt of consent, treatment assignment (high or low transfusion threshold group) will be made using a telephone system of randomization by the Data Coordinating Center (RTI International). Randomization will be performed within the appropriate birthweight and center stratum. Detailed instructions for treatment allocation and implementation and monitoring of the study protocol will be given to the research nurse and to on-call physicians in each participating center.

6.4 Prevention of Bias in an Unblinded Trial

All eligible infants of ≤1000 g birth weight and gestational age 22 0/7 through 28 6/7 weeks will be screened and logged, reducing selection bias. Randomization will be concealed, preventing allocation bias. It is impossible practically or ethically to blind either the caregivers or parents to the assigned hemoglobin threshold. Interim results will be presented to the Data Safety and Monitoring Committee (DSMC) with treatment assignments labeled only as group “A” or “B”. We will record all hemoglobin measures and transfusions given in order to assess protocol compliance. Site investigators will review compliance with clinicians at the onset of the trial and thereafter monthly as appropriate. Examiners at the 22-26 month examination will be unaware of treatment assignment, as will the radiologists interpreting the brain ultrasounds. Within NRN centers, local site interpretations were highly accurate and inter-rater reliability was excellent for the diagnosis of grade 3 or 4 IVH.[136]

6.5 Maintaining Adherence to Algorithm and Minimizing Deviation from Protocol

As noted above the algorithm will drive the majority of the transfusions. In exceptional circumstances, as defined below, the algorithm may be set aside until the infant is deemed stable to allow resumption of transfusion by study algorithm. Examples of such situations are infants with shock, severe sepsis with coagulopathy; or need for perioperative transfusion,

where the critical clinical indication for transfusion will override the threshold. In the PINT multicenter trial, the rate of deviation from protocol was acceptably low (15.9% in the low threshold arm and 6.7% in the high threshold arm). We assess adherence and separation of arms by recording mean Hgb at transfusion and at discharge. No Hgb determinations are mandated by study, but only as clinically ordered.

6.6 Choice of Primary Outcome

We will use a composite outcome of death or significant neurodevelopmental impairment in survivors at 22-26 months of corrected age. Children will be assessed at 22-26 months of corrected age by staff unaware of assignment. Centers will schedule visits as early as possible within this window. Outcome assessments will be conducted by physicians or nurses specializing in neonatal follow-up examination and certified psychologists. Neurodevelopmental Impairment will be defined in this study, as one or more of the following: cognitive delay, moderate or severe cerebral palsy, or severe visual or hearing impairment, as defined below.

Cognitive delay will be assessed by certified psychologists will assess infants using the Bayley Scales of Infant Development III (BSID-III). Cognitive delay is defined as BSID-III cognitive score < 85 (>1 standard deviation below the mean).

Cerebral Palsy is diagnosed if the child has a non-progressive motor impairment characterized by abnormal muscle tone and impaired range or control of movements. Medical history, physical examination and neurological examinations will be used to determine the presence of moderate or severe cerebral palsy (CP). Moderate CP is defined as level II or III on the Gross Motor Function Classification Scale (GMFCS).[125,126] Severe or non-ambulatory CP consists of GMFCS IV or V.

Severe vision impairment; is defined as corrected visual acuity in the better eye less than 20/200 determined by pediatric ophthalmologists.

Severe hearing impairment, is defined as bilateral hearing loss requiring amplification or the insertion of a cochlear implant as determined by a sound field hearing assessment or auditory brainstem responses.

The primary outcome will be deemed present if one or more of the individual components of the composite outcome are known to be present, or absent if no component was present. If no component was present, one or more missing components will cause the primary outcome to be deemed missing.

Cognitive delay is defined using the cutoff of 85 in Cognitive score because the Bayley III cognitive score below 70 identifies a smaller group than the Bayley II MDI below 70. This is because the Bayley III administered at 18-22 months corrected age among children born extremely preterm results in higher scores than the Bayley II.[137] One of two alternative explanations explains this difference. Either we collectively have previously underestimated cognitive development, or current testing overestimates cognitive outcomes. We believe a conservative approach to sample size will account for potential measurement error at 22-26 months. Although the more common composite primary outcome uses a cognitive score cutoff of 70 (*i.e.*, 2 SD below the mean), we have chosen a cutoff score of 85, to answer possible effects on neurodevelopment raised by PINT, and to allow a conservative approach regarding the sample size.

At the 22-26 month follow-up, each of the composite parts of the Primary Outcome will be examined also as a secondary outcome. All secondary outcomes at this time point are listed, but they will include: behavioral, emotional and social problems as evaluated by the Child Behavior Checklist 1.5 to 5. This is a 99 item instrument that has been widely used in follow up studies. It is being utilized by the Neonatal Research Network in the Follow Up Study for extremely low birth weight infants, as well as in the follow up components of clinical other trials.

6.7 Main secondary Outcomes

Secondary outcomes are measured at two time points:

(A) During initial hospitalization: (i) Ultrasound grade 3 or 4 IVH, cystic PVL, or ventriculomegaly; (ii) Bell stage 2 or 3 NEC; (iii) Apnea mandating either caffeine or respiratory support; (iv) Age at final tracheal extubation; (v) Number of donor exposures by RBC donors or other blood product; (vi) Number of transfusions; (vii) ROP stage 3 or greater or requiring treatment; (viii) Time to regain birth weight; (ix) Time to full feedings; (x) Death before discharge; (xi) BPD at 36 weeks postmenstrual age based on need for oxygen supplementation following an oxygen reduction test; (xii) Decreased height, weight, or head circumference at 36 weeks postmenstrual age; (xiii) length of hospital stay at Level 3 care; and (xiv) Costs of hospitalization.

(B) At 22-26 months corrected age, each of the composite outcomes individually: (i) Moderate or severe cerebral palsy; (ii) Severe vision impairment; (iii) Severe hearing impairment; (iv) BSID-III Cognitive score < 85; (v) BSID-III cognitive, language, or motor score <70; (vi) Gross Motor Function level ≥ II; (vii) CBCL Internalizing, Externalizing, and Total Problems aggregate T scores of >= 64 (clinical range) and 60-63 (borderline range) will be evaluated. (viii) Death before 22-26 month follow-up; (ix) Decreased height, weight, or head circumference at 22-26 month assessment; and (x) Cost-benefit analysis of primary outcome at 22-26 months.

Analyses of all primary and secondary outcomes will be adjusted for the design effects of stratification by center and birth weight group, and correlation between multiple births.

6.8 Potential Confounders and Strategy to Limit their Impact

(i) Severity of initial illness: Randomization should achieve balance at baseline. Adjustment for the SNAPPE score[138] on admission to NICU will be performed *post-hoc* if imbalances arise.

(ii) Blood bank practices**:** Center stratification will ensure balance across study.

(iii) Gender of blood donor: A retrospective cohort study of infants with birth weight <1500 g suggested that transfusion from male donors is associated with an increase in rate of NEC.[64] Thus, the proportion or total volume of transfused blood from male donors will be covariates in the model to predict NEC. This will be collected while preserving HIPPA anonymity, after the main trial is complete. This will be done by only using the blood unit ID number. This will be relayed to the issuing blood banks, who would provide only the gender of the donor to the investigators.

(iv) Sociological determinants**:** Home environmental influences and potential enhanced neurodevelopmental programs will be recorded as highest maternal educational levels, at enrollment. Adjustment for these will be conducted in secondary analyses.

6.9 Statistical Design Considerations

The primary objective of the TOP trial is to assess the difference in the primary outcome of survival without significant NDI (or, equivalently, death or NDI) at 22-26 months adjusted age in the two treatment arms. This outcome is similar to that used in the PINT trial, which found rates of death or neurosensory impairment at 18 months of 45.2% in the low trigger group and 38.5% in the high trigger group. Based on these findings, we believe that an absolute difference of 7% is the minimal clinically significant difference we would wish to detect in the rate of death or NDI. We considered several other factors in estimating the sample size for this study. First, we calculated the rate of death or NDI among infants born at NRN centers in 2005-2008 with GA 22-26 weeks and birth weight <1000 g who survived to 12 hours, which was 52%. However, we recognized that this population differed somewhat from that of the TOP trial, which proposes to enroll infants less than 29 weeks GA. In addition, the current NRN definition of NDI incorporates only the cognitive composite score of the Bayley III, while future definitions will likely also involve the Bayley III motor scales which have been collected in the Network only since January 2010. Thus, we recognized the limitations of estimating our sample size based only on current NRN data. At the same time, however, we were aware that the most conservative sample size estimate would result from an event rate of 50%, which current NRN data indicated was not implausible in our population. This led us to choose a conservative estimate based on assumed outcome rates of 53.5% and 46.5% (i.e., centered around an overall event rate of 50%) in the two treatment groups. With a two-tailed alpha of 0.05 and 80% power, the initial sample size estimate was 1,658 infants. The sample size was additionally inflated by 10% to account for loss to follow up at 22-26 months, yielding a final sample size estimate of 1,824.

Although the sample size calculations described above were based on the primary outcome, we will have 80% power with a two-tailed alpha of 0.05 to detect the following differences in important secondary outcomes (rates in the low threshold group are based on findings of the PINT trial): any transfusion 89.0% in the low threshold group (LOW) vs. 92.9% in the high group (HI); NEC 8.5% in LOW vs. 5.0% in HI; brain injury (PVL, echodense lesions or ventriculomegaly) on latest ultrasound (US) or neuroimaging closest to 36 weeks 18.5% in LOW vs. 24.6% in HI; death before discharge 21.5% in LOW vs. 16.1% in HI; combined death, severe ROP, BPD, or US brain injury 74.0% in LOW vs. 67.7% in HI.

6.10 Analysis Plan

***Main Study*:** All analyses will be conducted on an intention-to-treat basis, with the caveat that infants surviving to discharge, but lost to follow-up at 22-26 months, will be excluded from the primary analysis and considered ignorably missing. We will, however, assess the impact of outcomes lost to follow-up on our results by performing sensitivity analyses that assume all positive or all negative outcomes for those with missing data. We will also conduct bivariate analyses to compare the in-hospital characteristics of children lost to follow-up versus those with assessment of the primary outcome. Note that, based on previous NRN trials in similar populations, we expect the follow-up rate among survivors to be above 90%.

All analyses will be adjusted for design effects: stratification by center and birth weight group. The primary outcome of death or NDI at 22-26 months adjusted age will be analyzed using robust Poisson regression. Adjusted relative risks and 95% confidence intervals will be reported. Secondary analyses of the primary outcome may adjust for additional baseline covariates such as SNAPPE scores at NICU admission and socioeconomic status..

The consistency of the treatment effect over birth weight strata and gender, and treatment heterogeneity across the different centers, will be investigated.

Secondary outcomes including comorbidities such as NEC, ROP, BPD, and NDI among survivors at follow up, as well as other binary outcomes, will be analyzed using robust Poisson regression to produce adjusted relative risk estimates for the treatment effect, as described above. Models may also be adjusted for covariates known to be associated with the secondary outcomes in this population. For example, both gender and socioeconomic status have been implicated as crucial confounders for cognitive outcomes among survivors at follow up (Nopoulos P 2011); thus, it may be appropriate to include them as covariates in models for such outcomes. Number of transfusions will be analyzed using Poisson regression. Other secondary outcomes, such as time to regain birth weight and time to full feeds, will be analyzed using Cox proportional hazards survival regression. Although the basic Cox model assumes proportional hazards between groups, if this assumption is found to be violated in our data, time-varying covariates can be added to the models to allow for changes in the proportion over time. Weekly measures of growth (weight, length, head circumference) will be analyzed using longitudinal models to test whether growth trajectories differ between treatment groups. All models incorporating the treatment effect will be adjusted for the stratification variables (center and birth weight group). All secondary outcomes analyses will be considered exploratory because the trial is not primarily powered to detect these associations.

Economic Evaluation**:** We will conduct a formal, prospective economic evaluation with patient-level cost and efficacy data, using standard methods used in similar multicenter trials (56-60). Medical expenditure data from hospitals and families will be collected. The analysis will first directly compare mean costs between the two treatment groups using generalized linear modeling to account for the typically right-skewed cost data distribution. We will then calculate the incremental cost-effectiveness ratio, defined as the difference in mean cost per patient in the two groups, divided by the difference in the mean effect between the study arms. The outcome will thus be expressed as the cost per survivor free of significant NDI. To assess statistical uncertainty in the joint distribution of costs and effects, we will use nonparametric bootstrapping and report results as cost-effectiveness accepTOPlity curves. Finally, we will assess uncertainty in parameter values by using sensitivity analysis, in which we will recalculate the cost-effectiveness after varying the input values for certain variables through a plausible range. Further details are provided in the fuller protocol.

6.11 Data and Information Management

The data management system (DMS) recommended for the TOP study is a general purpose distributed data entry and management system developed to enable clinics to enter data from medical record extractions or other sources. This table-driven system is designed in a way that allows quick implementation of new studies into the existing system. Versions of this system, continually updated, have been successfully used for data collection for all NRN studies since 1998. An expanded version of this section is contained in the 'Data Safety and Monitoring Plan'(**Appendix A)**.

7.0
Methods

7.1 Randomization Method

Eligible infants whose parents consent to enrollment will be randomized to the high or low threshold group. Permuted block randomization with randomly chosen block sizes of two and four will be performed. The allocation ratio will be 1:1 and infants will be stratified by study center and birth weight (<750 g versus 750-999 g). Multiple births, which are common in this premature population (comprising 26% of infants <29 weeks gestational age in the 2009 NRN Generic Data Base), will be randomized independently. The study allocation (low or high threshold) will be randomly assigned to each patient number (to be assigned to enrolled patients sequentially within each center), in advance, using a computer-based random number generator. Study personnel at each center will telephone a secure automated toll-free line at RTI to obtain the randomized assignment for each enrolled patient. Back-up personnel at RTI will be in place in case of failure of the automated system, to ensure 24/7/365 randomization. Details of interaction of RTI with study sites, and clinical case record forms (CRFs) are provided in the Manual of Procedures (MOP).

7.2 Recruitment and Enrollment Plans

The NRN, with its 18 large academic medical centers, several with large satellite sites of their own, is uniquely positioned to successfully complete this rather large and ambitous neonatal trial. The trial poses an important and unresolved question in neonatal medicine, and both the PINT and Iowa trials indicate collective equipoise and high consent rates (>70%). Moreover, NRN centers have demonstrated the ability to recruit and randomize patients at all hours in previous studies where time was an important factor, and have an impressive track record in following infants through 18-22 months of age.. The NRN has achieved > 90% outcome assessment at 18-22 months follow-up in each of its previous trials. The most recent examples are the Phototherapy and Hypothermia trials, where >91% subjects had primary outcomes at 18-22 months and 6-7 years of age, respectively (47,50). In Table 8, below we provide different scenarios on the recruitment timeline for the TOP trial, based on most recent numbers from the NRN Generic Database. It demonstrates that this trial can indeed be successfully completed by the NRN centers within the requested funding period.

Table 8 Enrollment Timeline for the TOP Trial

Predicted GDB Enrollment Per Month	Percent		Recruited Per Month (per center)	Estimated Total Time to Enroll
	Consent Rate	Unable to Recruit for Other Reasons		
152	70	10	91 (5.7)	20 months
152	60	10	76 (4.7)	24 months
152	50	10	60 (3.8)	30 months

Similar to other NRN trials, recruitment will be monitored closely by the DCC through regular enrollment reports, and low enrollment at a center will prompt the CCC, DCC, NIH, and

other members of the trial subcommittee to hold conference calls with such centers to identify problems and develop solutions.

Although the acute enrollment is envisaged to be for 30 months, the follow-up phase will be longer. Our window for the follow-up is centered on 24 months, and extends from 22 to 26 months.

8.0
Data Safety and Monitoring

Data Safety Monitoring Committee: The NRN has a standing, independent Data Safety Monitoring Committee (DSMC) which monitors all Network trials. Because the proposed study will be conducted within the NRN, and because the procedures of this DSMC are consistent with NHLBI policies for Monitoring Boards for Data and Safety, the NICHD DSMC will monitor patient safety for this protocol. The committee includes experts in neonatology, maternal and fetal medicine, neurodevelopment, epidemiology, biostatistics, bioethics, high-risk infant follow-up, and clinical trials. A neonatal transfusion specialist will be added to the DSMC for the purposes of this study. An expanded version of this section is contained in the 'Data Safety and Monitoring Plan' (**Appendix A)**.

9.0
Study Organization and Administration

9.1 Study Sites

The study organization is guided by the procedures of the Neonatal Research Network. The Network is a collaboration among eighteen clinical centers appointed after competitive review, the DCC, and the NICHD. The Network has a Steering Committee consisting of the eighteen principal investigators (PIs) of the clinical centers, the NICHD Program Officer Dr. Rosemary Higgins, and an independent chairman. The Committee, which meets quarterly, sets policy and makes decisions.

9.2 Regulatory Compliance and Monitoring

The TOP DCC will operate under guidelines to ensure data accuracy, integrity, confidentiality, and security. DCC activities are governed formally by RTI Standard Operating Procedures or SOPs or less formally through technical operating procedures and guidance documents, which also help ensure regulatory compliance at the clinical centers.

We will continuously monitor data quality using point-of-entry quality checks in the DMS and other cross-form or longitudinal checks indicated by the investigators. We will identify errors, missing forms/items, and post center-specific reports on the private portion of the NRN website so that clinical staff can follow up. We have used this approach successfully for the NRN and other studies, producing automated routine reports on enrollment, retention, missing visits/items, and other problems, that enable us to evaluate general center performance and quickly initiate corrective actions as needed.

We will conduct targeted site visits using data audits on accrual, drop-outs, unresolved queries, and protocol violations to inform cost-efficient site monitoring decisions (54). Established checklists and reporting templates will be used to ensure that centers have the necessary resources; review research records and regulatory documents and observe study procedures, such as screening and recruitment. Following the site visit, the DCC will provide a written report to the center and NIH, and follow up on unresolved issues.

9.3 Recruitment Pace

Similar to other NRN trials, recruitment will be monitored closely by the DCC through regular enrollment reports, and low enrollment at a center will prompt the CCC, DCC, NIH, and other members of the trial subcommittee to hold conference calls with such centers to identify problems and develop solutions.

10.0
Ethics

10. Ethics

10.1 Risk Category, Privacy

We avoid breaching current practice boundaries of thresholds of hemoglobin that would trigger a transfusion by assuring broad buy-in of NRN clinicians and a transfusion algorithm that was developed by consensus. This approach avoids the dilemmas sometimes faced by other trials.[134]

This open trial, allows clinicians to bypass the study transfusion algorithm in circumstances where an infant's condition warrants acute transfusion, but then revert to the algorithm thereafter. The potential risks to subjects participating in this study or their parents are categorized in this study as 'minimal risk'. The trial maneuvers – other than randomization – are all within the range of current clinical practice and well within the likely variation of normal clinical practice.

We do not mandate any additional blood sampling nor specific route of sampling of blood, thus not departing from the standard practice of the NICU. The informed consent document and patient information materials will be thoroughly reviewed with the parents prior to their completing the written informed consent. Families will be given ample opportunity to ask questions regarding alternative options.

The basic rights of study participants will be respected and maintained by the investigators and by all who are involved in the collection or processing of individually identified data. Our data collection and processing procedures are designed to protect individual rights and to comply with all applicable laws and ethical principles. All staff who conduct or support research involving human subjects are required to undergo training on the protection of human subjects in research.

Minimizing risks from procedures used to collect data is the responsibility of the clinical centers and the DCC, although procedures to minimize risk will be described in the study protocols and include medical management common for this study population. Confidentiality procedures for subject data will be established by the trial investigators before data are transferred to RTI. All subject-identifying data will be kept solely at the clinical centers. The data collection forms will include unique study ID numbers only and basic demographic data as participant identifiers. Thus, the files maintained at RTI will contain limited identifying information and protect subject confidentiality. Safeguards are in place to greatly decrease the chances that characteristics of a case can be linked to the individual participating in the study.

The DCC will receive subject data from the clinical centers identified by a study ID only and will never have contact with the subjects. All procedures related to DCC activities, including data transmission and data security procedures, are reviewed and approved by the RTI IRB prior to receipt of any study data at RTI.

10.2 Consent and Oversight from NRN and RTI, and Interaction with Local IRBs

All NRN clinical centers and RTI have IRB committees that convene on a fixed schedule every month to review protocols and associated informed consent forms and data collection procedures for all research to ensure that they are in compliance with all applicable human subject regulations. IRB approval must be granted prior to beginning any study, and study progress and procedures must be reviewed by the IRB at least annually.

Potential subjects or their legal guardians must be fully informed about the details of any research study in which they are considering participation and what their involvement will entail. Specific consent forms are developed for each protocol and reviewed and approved by the IRBs at RTI and at each clinical center. RTI reviews forms used at the sites to ensure that essential elements of consent are presented and comply with federal law

All NRN clinical centers and the DCC at RTI have Federal wide Assurance (FWA) by the Office for Human Research Protections (OHRP) of the Department of Health and Human Services (HHS). This FWA is an agreement between each center, RTI and the U.S. government that all research with human subjects will be conducted according to appropriate federal regulations and allows us to undertake its own Institutional Review Board (IRB) review and monitoring of research with human subjects. Each NRN institution participating in the TOP trial holds an FWA, which ensures that the institution's human research activities, overseen by their regulatory authorities, comply with the requirements set forth in 45 CFR 46, as well as the terms of Assurance. The DCC at RTI is responsible for obtaining appropriate clearances at our institution with respect to HIPAA and Human Subjects Research regulations and verifying similar clearances at the clinical centers.

TOP trial study procedures are subject to the approval of the IRBs at the participating clinical centers and the DCC. For this trial, the protocol, informed consent (IC), and other study documents used at each center must be reviewed and approved by the respective IRBs before the study is initiated. The IRBs monitor the research process to ensure that the procedures for protecting human subject rights are followed. Every protocol is reviewed by each IRB at least annually. Each IRB reviews AE reports and approves all proposed changes to a research protocol before any changes are implemented. If necessary, the IRBs will mandate changes needed to protect the rights and welfare of human subjects or suggest solutions for problems that arise during a project.

After IRB approval, infants eligible for the TOP trial will be identified by the clinical center PI or study staff from among the infants who are cared for in the NRN Neonatal Intensive Care Units. The PI or their staff will explain to the parents (1) consent forms that have been specifically to address the nature, duration, and purpose of the study; (2) means by which it is to be conducted; (3) possible benefit or lack of benefits; (4) potential risks, hazards, and discomforts; and (5) possible alternative procedures. Specific decisions regarding the operational details of how consent will be sought (including the timing and the level of details presented regarding various aspects of the study) are made at the level of each clinical center under the guidelines of their local IRBs and practice policies and traditions.

NICHD and NHLBI will defer to local IRB ruling for the conduct of the TOP trial at all the clinical centers. Should a local IRB determine safety issues and require actions, including any related to suggested changes to the consent form, the local center research staff (investigator/coordinator) will notify the NRN DCC (RTI), the NHLBI and NICHD Program Scientists via phone or email within 24 hours of discovery. NHLBI and NICHD will direct the

DCC in further action. Note that any changes to the protocol itself (directed either by any IRB or the NRN DSMC) will require discussion and approval by the TOP trial subcommittee, NHLBI and NICHD. The DCC will maintain all communications between the clinical center(s), NHLBI and NICHD through resolution of all these IRB issues.

Conflicts of Interest

We will ensure that no member appointed to the DSMC has a conflict of interest, and they will be required to sign to that effect. This is in the DSMC charter. No one on the study team including the proposed steering committee of the trial, has any financial interests related to either blood products, or alternatives to standard blood based infusion fluids. Nor do they act as consultants to any commercial blood product agencies.

10.3 Involvement of Human Subjects

The primary goal of this study is to conduct a randomized clinical trial of varying transfusion thresholds in extremely low birth weight (≤1000 g) or ELBW newborns to determine the effects of relatively high or low transfusion triggers on long-term survival and neurodevelopmental outcomes in this population. The study will involve this population, with baseline and sociodemographic data, as well as informed consent obtained from parents or legal guardians.

In addition to our internal safeguards, we have a Data Safety and Monitoring Committee (DSMC) and a Data Safety and Monitoring Plan (**Appendix A)**, which will be implemented together with NICHD, the institute that oversees the Neonatal Research Network and will support this trial.

References

1. Kirpalani H, Whyte RK, Andersen C, *et al*. The Premature Infants in Need of Transfusion (PINT) study: a randomized, controlled trial of a restrictive (low) versus liberal (high) transfusion threshold for extremely low birth weight infants. *J Pediatr.* 2006;149:301-7. PMID: 16939737.

2. Whyte RK, Kirpalani H, Asztalos EV, *et al*. Neurodevelopmental outcome of extremely low birth weight infants randomly assigned to restrictive or liberal hemoglobin thresholds for blood transfusion. *Pediatrics* 2009;123: 207-13. PMID: 19117884

3. Kamholz KL, Dukhovny D, Kirpalani H, *et al*. Economic evaluation alongside the Premature Infants in Need of Transfusion randomised controlled trial. *Arch Dis Child Fetal Neonatal Ed*. 2012;97(2):F93-8. PMID: 21733926

4. Bell EF, Strauss RG, Widness JA, *et al*. Randomized trial of liberal versus restrictive guidelines for red blood cell transfusion in preterm infants. *Pediatrics* 2005; 115: 1685–91. PMCID: 2866196

5. Nopoulos PC, Conrad AL, Bell EF, Strauss RG, Widness JA, Magnotta VA, et al. Long-term Outcome of Brain Structure in Premature Infants: Effects of Liberal vs Restricted Red Blood Cell Transfusions. *Arch Pediatr Adolesc Med.* 2011;165(5):443-50. PMID: 21199970

6. McCoy TE, Conrad AL, Richman LC, Lindgren SD, Nopoulos PC, Bell EF. Neurocognitive profiles of preterm infants randomly assigned to lower or higher hematocrit thresholds for transfusion. *Child Neuropsychol.* 2011;17(4):347-67. PMID: 21360360

7. National Center for Health Statistics; 2009 http://marchofdimes.com/peristats/-April2009

8. Behrman RE, Butler AS. Preterm Birth: Causes, Consequences, and Prevention. Washington, DC: National Academy Press; 2006.

9. Hintz SR, Kendrick DE, Wilson-Costello DE, Das A, Bell EF, Vohr BR, et al. Early-childhood neurodevelopmental outcomes are not improving for infants born at <25 weeks' gestational age. Pediatrics. 2011;127(1):62-70.

10. Marlow N, Wolke D, Bracewell MA, Samara M. Neurologic and developmental disability at six years of age after extremely preterm birth. N Engl J Med. 2005;352(1):9-19.

11. Hack M, Taylor HG, Drotar D, Schluchter M, Cartar L, Andreias L, et al. Chronic conditions, functional limitations, and special health care needs of school-aged children born with extremely low-birth-weight in the 1990s. JAMA. 2005;294(3):318-25.

12. Swamy GK, Ostbye T, Skjaerven R. Association of preterm birth with long-term survival, reproduction, and next-generation preterm birth. JAMA. 2008;299(12):1429-36.

13. Saigal S, Stoskopf B, Pinelli J, Streiner D, Hoult L, Paneth N, et al. Self-perceived health-related quality of life of former extremely low birth weight infants at young adulthood. Pediatrics. 2006;118(3):1140-8.

14. Gray R, Petrou S, Hockley C, Gardner F. Self-reported health status and health-related quality of life of teenagers who were born before 29 weeks' gestational age. Pediatrics. 2007;120(1):e86-93.

15. Zwicker JG, Harris SR. Quality of life of formerly preterm and very low birth weight infants from preschool age to adulthood: a systematic review. Pediatrics. 2008;121(2):e366-76.

16. Maier RF, Sonntag J, Walka MM, Liu G, Metze BC, Obladen M. Changing practices of red blood cell transfusions in infants with birth weights less than 1000 g. J Pediatr. 2000;136(2):220-4.
17. Blanchette VS, Zipursky A. Assessment of anemia in newborn infants. Clin Perinatol. 1984;11(2):489-510.
18. Widness JA. Pathophysiology of Anemia During the Neonatal Period, Including Anemia of Prematurity. Neoreviews. 2008;9(11):e520. PMCID: 2867612.
19. Nexo E, Christensen NC, Olesen H. Volume of blood removed for analytical purposes during hospitalization of low-birthweight infants. Clin Chem. 1981;27(5):759-61.
20. Stockman JA, 3rd. Anemia of prematurity. Current concepts in the issue of when to transfuse. Pediatr Clin North Am. 1986;33(1):111-28.
21. Widness JA, Seward VJ, Kromer IJ, Burmeister LF, Bell EF, Strauss RG. Changing patterns of red blood cell transfusion in very low birth weight infants. J Pediatr. 1996;129(5):680-7.
22. Sacher RA, Strauss RG, Luban NL, Feil M, Anstall HB, Barnes A, Jr., et al. Blood component therapy during the neonatal period: a national survey of red cell transfusion practice, 1985. Transfusion. 1990;30(3):271-6.
23. Strauss RG. Red blood cell transfusion practices in the neonate. Clin Perinatol. 1995;22(3):641-55.
24. Weiss M, Fischer J, Boeckmann M, Rohrer B, Baenziger O. Evaluation of a simple method for minimizing iatrogenic blood loss from discard volumes in critically ill newborns and children. Intensive Care Med. 2001;27(6):1064-72.
25. Lin JC, Strauss RG, Kulhavy JC, Johnson KJ, Zimmerman MB, Cress GA, et al. Phlebotomy overdraw in the neonatal intensive care nursery. Pediatrics. 2000;106(2):E19.
26. Donato H, Vain N, Rendo P, Vivas N, Prudent L, Larguia M, et al. Effect of early versus late administration of human recombinant erythropoietin on transfusion requirements in premature infants: results of a randomized, placebo-controlled, multicenter trial. Pediatrics. 2000;105(5):1066-72.
27. Maier RF, Obladen M, Muller-Hansen I, Kattner E, Merz U, Arlettaz R, et al. Early treatment with erythropoietin beta ameliorates anemia and reduces transfusion requirements in infants with birth weights below 1000 g. J Pediatr. 2002;141(1):8-15.
28. Balin A, Koren G, Hasu M, Zipursky A. Evaluation of a new method for the prevention of neonatal anemia. Pediatr Res. 1989;25(3):274-5.
29. Widness JA, Madan A, Grindeanu LA, Zimmerman MB, Wong DK, Stevenson DK. Reduction in red blood cell transfusions among preterm infants: results of a randomized trial with an in-line blood gas and chemistry monitor. Pediatrics. 2005;115(5):1299-306.
30. Vamvakas EC, Strauss RG. Meta-analysis of controlled clinical trials studying the efficacy of rHuEPO in reducing blood transfusions in the anemia of prematurity. Transfusion. 2001;41(3):406-15.
31. Ohlsson A, Aher SM. Early erythropoietin for preventing red blood cell transfusion in preterm and/or low birth weight infants. Cochrane Database Syst Rev. 2006;3:CD004863.
32. Ohls RK, Ehrenkranz RA, Wright LL, Lemons JA, Korones SB, Stoll BJ, et al. Effects of early erythropoietin therapy on the transfusion requirements of preterm infants below 1250

grams birth weight: a multicenter, randomized, controlled trial. Pediatrics. 2001;108(4):934-42.

33. Ohls RK, Ehrenkranz RA, Das A, Dusick AM, Yolton K, Romano E, et al. Neurodevelopmental outcome and growth at 18 to 22 months' corrected age in extremely low birth weight infants treated with early erythropoietin and iron. Pediatrics. 2004;114(5):1287-91

34. Bierer R, Peceny MC, Hartenberger CH, Ohls RK. Erythropoietin concentrations and neurodevelopmental outcome in preterm infants. Pediatrics. 2006;118(3):e635-40.

35. Rabe H, Reynolds G, Diaz-Rossello J. Early versus delayed umbilical cord clamping in preterm infants. Cochrane Database Syst Rev. 2004(4):CD003248.

36. Rabe H, Reynolds G, Diaz-Rossello J. A systematic review and meta-analysis of a brief delay in clamping the umbilical cord of preterm infants. Neonatology. 2008;93(2):138-44.

37. Reynolds GJ. Beyond sweetness and warmth: transition of the preterm infant. Arch *Dis Child Fetal Neonatal Ed*. 2008;93(1):F2-3.

38. Ghavam S, Batra G, *et al*. Placental Transfusion Strategies in Preterms <1000 g BW: Meta-Analysis of Short and Long Term Outcomes. (Presented to the Society for Pediatric Research, Denver, Colorado, April 30 -May 3, 2011).

39. Sullivan MT, Cotten R, Read EJ, Wallace EL. Blood collection and transfusion in the United States in 2001. *Transfusion.* 2007;47(3):385-94.

40. Whyte R, Kirpalani H. Low versus high haemoglobin concentration threshold for blood transfusion for preventing morbidity and mortality in very low birth weight infants. *Cochrane Database Syst Rev*. 2011 Nov 9;11:CD000512.

41. Guillen U, Cummings J, Bell EF, *et al*. International survey of transfusion practices for extremely premature infants. (Presented to the Society for Pediatric Research, Denver, Colorado, April 30 -May 3, 2011).

42. Shannon KM, Keith JF, 3rd, Mentzer WC, Ehrenkranz RA, Brown MS, Widness JA, et al. Recombinant human erythropoietin stimulates erythropoiesis and reduces erythrocyte transfusions in very low birth weight preterm infants. *Pediatrics.* 1995;95(1):1-8.

43. Miyashiro AM, Santos N, Guinsburg R, Kopelman BI, Peres Cde A, Taga MF, et al. Strict red blood cell transfusion guideline reduces the need for transfusions in very-low-birthweight infants in the first 4 weeks of life: a multicentre trial. *Vox Sang.* 2005;88(2):107-13.

44. Guidelines for transfusion of erythrocytes to neonates and premature infants. Fetus and Newborn Committee, Canadian Paediatric Society. CMAJ. 1992;147(12):1781-92. PMCID: 1336654.

45. Valieva OA, Strandjord TP, Mayock DE, Juul SE. Effects of transfusions in extremely low birth weight infants: a retrospective study. *J Pediatr*. 2009;155(3):331-37 e1. PMCID: 3038786.

46. Hebert PC, Wells G, Blajchman MA, Marshall J, Martin C, Pagliarello G, et al. A multicenter, randomized, controlled clinical trial of transfusion requirements in critical care. Transfusion Requirements in Critical Care Investigators, Canadian Critical Care Trials Group. *N Engl J Med.* 1999;340(6):409-17.

47. Hill SR, Carless PA, Henry DA, Carson JL, Hebert PC, McClelland DB, et al. Transfusion thresholds and other strategies for guiding allogeneic red blood cell transfusion. *Cochrane Database Syst Rev.* 2002(2):CD002042.

48. Walsh TS. Red cell transfusion triggers in critically ill patients: time for some new TRICCs? *Crit Care*. 2010;14(3):170. PMCID: 2911743.

49. dos Santos AM, Guinsburg R, de Almeida MF, *et al*. Red blood cell transfusions are independently associated with intra-hospital mortality in very low birth weight preterm infants. *J Pediatr*. 2011 Sep;159(3):371-376.e1-3.

50. Kirpalani H, Whyte R. Truths, associations, and hypotheses. *J Pediatr*. 2011 Sep;159(3):359-61.

51. Ng PC, Lam CW, Lee CH, To KF, Fok TF, Chan IH, et al. Hepatic iron storage in very low birthweight infants after multiple blood transfusions. Arch Dis Child Fetal Neonatal Ed. 2001;84(2):F101-5. PMCID: 1721227.

52. Cooke RW, Clark D, Hickey-Dwyer M, Weindling AM. The apparent role of blood transfusions in the development of retinopathy of prematurity. Eur J Pediatr. 1993;152(10):833-6.

53. Hesse L, Eberl W, Schlaud M, Poets CF. Blood transfusion. Iron load and retinopathy of prematurity. Eur J Pediatr. 1997;156(6):465-70.

54. Dani C, Reali MF, Bertini G, Martelli E, Pezzati M, Rubaltelli FF. The role of blood transfusions and iron intake on retinopathy of prematurity. Early Hum Dev. 2001;62(1):57-63.

55. Cooke RW, Drury JA, Yoxall CW, James C. Blood transfusion and chronic lung disease in preterm infants. Eur J Pediatr. 1997;156(1):47-50.

56. Kuban KC. White-matter disease of prematurity, periventricular leukomalacia, and ischemic lesions. Dev Med Child Neurol. 1998;40(8):571-3.

57. Christensen RD. Association between red blood cell transfusions and necrotizing enterocolitis. J Pediatr. 2011;158(3):349-50.

58. Blau J, Calo JM, Dozor D, Sutton M, Alpan G, La Gamma EF. Transfusion-related acute gut injury: necrotizing enterocolitis in very low birth weight neonates after packed red blood cell transfusion. J Pediatr. 2011;158(3):403-9.

59. El-Dib M, Narang S, Lee E, Massaro AN, Aly H. Red blood cell transfusion, feeding and necrotizing enterocolitis in preterm infants. J Perinatol. 2011;31(3):183-7.

60. Singh R, Visintainer PF, Frantz ID, 3rd, Shah BL, Meyer KM, Favila SA, et al. Association of necrotizing enterocolitis with anemia and packed red blood cell transfusions in preterm infants. J Perinatol. 2011;31(3):176-82.

61. Mally P, Golombek SG, Mishra R, Nigam S, Mohandas K, Depalhma H, et al. Association of necrotizing enterocolitis with elective packed red blood cell transfusions in stable, growing, premature neonates. Am J Perinatol. 2006;23(8):451-8.

62. Reber KM, Nankervis CA, Nowicki PT. Newborn intestinal circulation. Physiology and pathophysiology. Clin Perinatol. 2002;29(1):23-39.

63. Josephson CD, Wesolowski A, Bao G, Sola-Visner MC, Dudell G, Castillejo MI, et al. Do red cell transfusions increase the risk of necrotizing enterocolitis in premature infants? J Pediatr. 2010;157(6):972-8 e1-3.

64. Paul DA, Mackley A, Novitsky A, Zhao Y, Brooks A, Locke RG. Increased odds of necrotizing enterocolitis after transfusion of red blood cells in premature infants. Pediatrics. 2011;127(4):635-41.

65. Luban NL. Management of anemia in the newborn. Early Hum Dev. 2008;84(8):493-8.

66. Ngo V, Civen R. Babesiosis acquired through blood transfusion, California, USA. Emerg Infect Dis. 2009;15(5):785-7. PMCID: 2687036.

67. Llewelyn CA, Hewitt PE, Knight RS, Amar K, Cousens S, Mackenzie J, et al. Possible transmission of variant Creutzfeldt-Jakob disease by blood transfusion. Lancet. 2004;363(9407):417-21.

68. Fergusson D, Hebert PC, Lee SK, Walker CR, Barrington KJ, Joseph L, et al. Clinical outcomes following institution of universal leukoreduction of blood transfusions for premature infants. JAMA. 2003;289(15):1950-6.

69. Ziemann M, Krueger S, Maier AB, Unmack A, Goerg S, Hennig H. High prevalence of cytomegalovirus DNA in plasma samples of blood donors in connection with seroconversion. Transfusion. 2007;47(11):1972-83.

70. Stockman JA, 3rd. The anemia of prematurity and the decision when to transfuse. Adv Pediatr. 1983;30:191-219.

71. Roberton NR. Does CPAP work when it really matters. Acta Paediatr. 1993;82(2):206-7.

72. Alkalay AL, Galvis S, Ferry DA, Simmons CF, Krueger RC, Jr. Hemodynamic changes in anemic premature infants: are we allowing the hematocrits to fall too low? Pediatrics. 2003;112(4):838-45.

73. Fredrickson LK, Bell EF, Cress GA, Johnson KJ, Zimmerman MB, Mahoney LT, et al. Acute physiological effects of packed red blood cell transfusion in preterm infants with different degrees of anaemia. Arch Dis Child Fetal Neonatal Ed. Epub 2010 Nov 20.

74. Bifano EM, Smith F, Borer J. Relationship between determinants of oxygen delivery and respiratory abnormalities in preterm infants with anemia. J Pediatr. 1992;120(2 Pt 1):292-6.

75. Keller A, Hermanussen M, Vogtmann C, Kiess W, Keller E. Effect of erythrocyte transfusion on longitudinal bone growth of premature infants assessed by mini-knemometry. Eur J Pediatr. 1999;158(10):871-2.

76. Wardle SP, Weindling AM. Peripheral fractional oxygen extraction and other measures of tissue oxygenation to guide blood transfusions in preterm infants. Semin Perinatol. 2001;25(2):60-4.

77. Wardle SP, Yoxall CW, Crawley E, Weindling AM. Peripheral oxygenation and anemia in preterm babies. Pediatr Res. 1998;44(1):125-31.

78. Wardle SP, Garr R, Yoxall CW, Weindling AM. A pilot randomised controlled trial of peripheral fractional oxygen extraction to guide blood transfusions in preterm infants. Arch Dis Child Fetal Neonatal Ed. 2002;86(1):F22-7. PMCID: 1721368.

79. Baenziger O, Keel M, Bucher HU, Wolf M. Oxygen extraction index measured by near infrared spectroscopy--a parameter for monitoring tissue oxygenation? Adv Exp Med Biol. 2009;645:161-6.

80. Dani C, Pratesi S, Fontanelli G, Barp J, Bertini G. Blood transfusions increase cerebral, splanchnic, and renal oxygenation in anemic preterm infants. Transfusion. 2010;50(6):1220-6.

81. van Hoften JC, Verhagen EA, Keating P, ter Horst HJ, Bos AF. Cerebral tissue oxygen saturation and extraction in preterm infants before and after blood transfusion. Arch Dis Child Fetal Neonatal Ed. 2010;95(5):F352-8.

82. Linder N, Haskin O, Levit O, Klinger G, Prince T, Naor N, et al. Risk factors for intraventricular hemorrhage in very low birth weight premature infants: a retrospective case-control study. Pediatrics. 2003;111(5 Pt 1):e590-5.

83. Mercer JS, Vohr BR, McGrath MM, Padbury JF, Wallach M, Oh W. Delayed cord clamping in very preterm infants reduces the incidence of intraventricular hemorrhage and late-onset sepsis: a randomized, controlled trial. Pediatrics. 2006;117(4):1235-42.

84. Baer VL, Lambert DK, Henry E, Snow GL, Christensen RD. Red blood cell transfusion of preterm neonates with a Grade 1 intraventricular hemorrhage is associated with extension to a Grade 3 or 4 hemorrhage. Transfusion. 2011.

85. Zhang B, Schmidt B. Do we measure the right end points? A systematic review of primary outcomes in recent neonatal randomized clinical trials. J Pediatr. 2001;138(1):76-80.

86. Glick HA, Doshi JA, Sonnad SS, et al. Economic Evaluation in Clinical Trials. New York, NY: Oxford University Press 2007. Procedures. Edmonton, AB: Airgas Puritan Medical, 2007.

87. McBride JA, Parad R, davis J, Allred L, Zupani J. Cost analysis on the use of recombinant human superoxide dismutase (rhSOD) at birth in preterm infants to improve pulmonary outcome. Pediatric Academic Societies (www.par-meeting.org). 2005;57:1232.

88. Zupancic JA, Richardson DK, O'Brien BJ, Cronin CG, Schmidt B, Roberts R, et al. Retrospective economic evaluation of a controlled trial of indomethacin prophylaxis for patent ductus arteriosus in premature infants. Early Hum Dev. 2006;82(2):97-103.

89. McBride JA, Parad RB, Davis JM, Zheng Z, Zupancic JA. Economic evaluation of recombinant human copper zinc superoxide dismutase administered at birth to premature infants. J Perinatol. 2009;29(5):364-71.

90. Zupancic JA, Hibbs AM, Palermo L, Truog WE, Cnaan A, Black DM, et al. Economic evaluation of inhaled nitric oxide in preterm infants undergoing mechanical ventilation. Pediatrics. 2009;124(5):1325-32.

91. Dukhovny D, Lorch SA, Schmidt B, Doyle LW, Kok JH, Roberts RS, et al. Economic evaluation of caffeine for apnea of prematurity. Pediatrics. 2011;127(1):e146-55.

92. Hospital Cost Report FY 2011. Center for Medicare and Medicaid Services. Available at: http://www.cms.hhs.gov/Research-Statistics-Data-and-Systems/Files-for-Order/CostReports/Cost-Reports-by-Fiscal-Year-Items/CMS1250866.html [Accessed April 12, 2012]

93. Finkler SA. The distinction between cost and charges. Ann Intern Med. 1982;96(1):102-9.

94. Taira DA, Seto TB, Siegrist R, Cosgrove R, Berezin R, Cohen DJ. Comparison of analytic approaches for the economic evaluation of new technologies alongside multicenter clinical trials. Am Heart J. 2003;145(3):452-8.

95. RBRVS: What is it and how does it affect pediatrics?: American Academy of Pediatrics; 2007 [cited 2006 December 20, 2006]; Available from: http://practice.aap.org/content.aspx?aID=1652 .

96. Hsiao WC, Braun P, Dunn D, Becker ER, DeNicola M, Ketcham TR. Results and policy implications of the resource-based relative-value study. N Engl J Med. 1988;319(13):881-8.

97. Briggs A, Clark T, Wolstenholme J, Clarke P. Missing... presumed at random: cost-analysis of incomplete data. Health Econ. 2003;12(5):377-92.

98. Manning WG, Basu A, Mullahy J. Generalized modeling approaches to risk adjustment of skewed outcomes data. J Health Econ. 2005;24(3):465-88.

99. Manning WG, Mullahy J. Estimating log models: to transform or not to transform? J Health Econ. 2001;20(4):461-94.

100. Efron B, Tibshirani RJ. An introduction to the boostrap. New York: Chapman and Hall/CRC; 1993.

101. Fenwick E, Claxton K, Sculpher M. Representing uncertainty: the role of cost-effectiveness accepTOPlity curves. Health Econ. 2001;10(8):779-87.

102. Fenwick E, O'Brien BJ, Briggs A. Cost-effectiveness accepTOPlity curves--facts, fallacies and frequently asked questions. Health Econ. 2004;13(5):405-15.

103. Polsky D, Glick HA, Willke R, Schulman K. Confidence intervals for cost-effectiveness ratios: a comparison of four methods. Health Econ. 1997;6(3):243-52.

104. Reikvam H, van de Watering L, Prowse C, Devine D, Heddle NM, Hervig T; BEST collaborative. Evaluation of noninvasive methods for the estimation of haemoglobin content in red blood cell concentrates. Transfus Med. 2011 Jun;21(3):145-9.

105. Adamson JW. New blood, old blood, or no blood? N Engl J Med. 2008;358(12):1295-6.

106. Zimrin AB, Hess JR. Current issues relating to the transfusion of stored red blood cells. Vox Sang. 2009;96(2):93-103.

107. Marik PE, Sibbald WJ. Effect of stored-blood transfusion on oxygen delivery in patients with sepsis. JAMA. 1993;269(23):3024-9.

108. Koch CG, Li L, Sessler DI, Figueroa P, Hoeltge GA, Mihaljevic T, et al. Duration of red-cell storage and complications after cardiac surgery. N Engl J Med. 2008;358(12):1229-39.

109. Tinmouth A, Fergusson D, Yee IC, Hebert PC. Clinical consequences of red cell storage in the critically ill. Transfusion. 2006;46(11):2014-27.

110. Roback J, Combs MR, MT(ASCP)SBB, Grossman B and Hillyer C. Technical Manual, 16th ed. Bethesda: American Association of Blood Banks (AABB); 2008.

111. Fasano R, Luban NL. Blood component therapy. Pediatr Clin North Am. 2008;55(2):421-45, ix.

112. Vamvakas E, Moore SB. Perioperative blood transfusion and colorectal cancer recurrence: a qualitative statistical overview and meta-analysis. Transfusion. 1993;33(9):754-65.

113. Blajchman MA. Allogeneic blood transfusions, immunomodulation, and postoperative bacterial infection: do we have the answers yet? Transfusion. 1997;37(2):121-5.

114. Houbiers JG, Brand A, van de Watering LM, Hermans J, Verwey PJ, Bijnen AB, et al. Randomised controlled trial comparing transfusion of leucocyte-depleted or buffy-coat-depleted blood in surgery for colorectal cancer. Lancet. 1994;344(8922):573-8.

115. Jensen LS, Kissmeyer-Nielsen P, Wolff B, Qvist N. Randomised comparison of leucocyte-depleted versus buffy-coat-poor blood transfusion and complications after colorectal surgery. Lancet. 1996;348(9031):841-5.

116. van de Watering LM, Hermans J, Houbiers JG, van den Broek PJ, Bouter H, Boer F, et al. Beneficial effects of leukocyte depletion of transfused blood on postoperative complications in patients undergoing cardiac surgery: a randomized clinical trial. Circulation. 1998;97(6):562-8.

117. Hebert PC, Wells G, Blajchman MA, Marshall J, Martin C, Pagliarello G, et al. A multicenter, randomized, controlled clinical trial of transfusion requirements in critical care. Transfusion Requirements in Critical Care Investigators, Canadian Critical Care Trials Group. N Engl J Med. 1999;340(6):409-17.

118. Fergusson D, Hebert PC, Lee SK, Walker CR, Barrington KJ, Joseph L, et al. Clinical outcomes following institution of universal leukoreduction of blood transfusions for premature infants. JAMA. 2003;289(15):1950-6.

119. McAlister FA, Clark HD, Wells PS, Laupacis A. Perioperative allogeneic blood transfusion does not cause adverse sequelae in patients with cancer: a meta-analysis of unconfounded studies. Br J Surg. 1998;85(2):171-8.

120. Thurer RL, Luban NL, AuBuchon JP, Silver H, McCarthy LJ, Dzik S, et al. Universal WBC reduction. Transfusion. 2000;40(6):751-2.

121. Luban NL. Transfusion safety: Where are we today? Ann N Y Acad Sci. 2005;1054:325-41.

122. Glynn SA. Blood supply safety: an NHLBI perspective. Transfusion. 2008;48(8):1541-4.

123. Gilbert GL, Hayes K, Hudson IL, James J. Prevention of transfusion-acquired cytomegalovirus infection in infants by blood filtration to remove leucocytes. Neonatal Cytomegalovirus Infection Study Group. Lancet. 1989;1(8649):1228-31.

124. Strauss RG. Data-driven blood banking practices for neonatal RBC transfusions. Transfusion. 2000;40(12):1528-40.

125. Rosenbaum PL, Walter SD, Hanna SE, Palisano RJ, Russell DJ, Raina P, et al. Prognosis for gross motor function in cerebral palsy: creation of motor development curves. JAMA. 2002;288(11):1357-63.

126. Palisano RJ, Cameron D, Rosenbaum PL, Walter SD, Russell D. STOPlity of the gross motor function classification system. Dev Med Child Neurol. 2006;48(6):424-8.

127. Ransome OJ, Moosa EA, Mothebe FM, Spector I. Are regular 'top-up' transfusions necessary in otherwise well, growing premature infants? S Afr Med J. 1989;75(4):165-6.

128. Blank JP, Sheagren TG, Vajaria J, Mangurten HH, Benawra RS, Puppala BL. The role of RBC transfusion in the premature infant. Am J Dis Child. 1984;138(9):831-3.

129. Bifano EM, Bode MM, D'Eugenio DB. Prospective randomized trial of high vs low hematocrit I ELBW infants: one year growth and neurodevelopmental outcome. Pediatr Red. 2002:51:325A.

130. Chen HL, Tseng HI, Lu CC, Yang SN, Fan HC, Yang RC. Effect of blood transfusions on the outcome of very low body weight preterm infants under two different transfusion criteria. Pediatr Neonatol. 2009;50(3):110-6.

131. Guidelines for transfusion of erythrocytes to neonates and premature infants. Fetus and Newborn Committee, Canadian Paediatric Society. CMAJ. 1992;147(12):1781-92. PMCID: 1336654.

132. Ventilation with lower tidal volumes as compared with traditional tidal volumes for acute lung injury and the acute respiratory distress syndrome. The Acute Respiratory Distress Syndrome Network. N Engl J Med. 2000;342(18):1301-8.

133. Mann H. Controversial choice of a control intervention in a trial of ventilator therapy in ARDS: standard of care arguments in a randomised controlled trial. J Med Ethics. 2005;31(9):548-53. PMCID: 1734226.

134. Deans KJ, Minneci PC, Suffredini AF, Danner RL, Hoffman WD, Ciu X, et al. Randomization in clinical trials of titrated therapies: unintended consequences of using fixed treatment protocols. Crit Care Med. 2007;35(6):1509-16.

135. Minneci PC, Eichacker PQ, Danner RL, Banks SM, Natanson C, Deans KJ. The importance of usual care control groups for safety monitoring and validity during critical care research. Intensive Care Med. 2008;34(5):942-7.

136. Hintz SR, Slovis T, Bulas D, Van Meurs KP, Perritt R, Stevenson DK, et al. Interobserver reliability and accuracy of cranial ultrasound scanning interpretation in premature infants. J Pediatr. 2007;150(6):592-6, 6 e1-5. PMCID: 2757063.

137. Vohr BR, Stephens BE, Higgins RD, Bann CM, et al. Are outcomes of extremely preterm infants improving? Impact of Bayley assessment on outcomes. J Pediatr. 2012 Aug;161(2):222-8.e3.

138. Zupancic JA, Richardson DK, Horbar JD, Carpenter JH, Lee SK, Escobar GJ. Revalidation of the Score for Neonatal Acute Physiology in the Vermont Oxford Network. Pediatrics. 2007;119(1):e156-63.

Appendix A: DATA AND SAFETY MONITORING PLAN

1. Composition of the Data and Safety Monitoring Board (DSMB)
TOP is a study which is investigator-initiated and sponsored by the Eunice Kennedy Shriver NICHD Neonatal Research Network (NRN). Accordingly the standing NRN Data Safety Monitoring Committee (hereafter referred to as 'DSMC') provides the basis of study safety oversight.

In order to ensure full co-oversight by the NHLBI, two representatives of the Blood division of NHLBI are appointed to the Board, which enables a joint oversight to be exercised. Precedence for this model comes from the NRN SUPPORT trial (co-funded by the NHLBI) where additional specialists joined the NRN DSMC for monitoring that trial. The joint oversight is provided by the two representatives of the Blood division on the DSMC. These are firstly Dr. Traci Mondoro MD (Project Officer for the Blood division of NHLBI), and Dr. Catherine S. Manno, a pediatric transfusion expert.

All members of the DSMC are completely independent of the study and the PIs and will be required to sign documentation to this effect. The NRN DSMC conforms to the recommended standards of the NIH (http://www.fda.gov/RegulatoryInformation/Guidances/ucm127069.htm).

We detail some specifics of DSMC functioning:

a) The executive secretary is Dr. Marion Willinger, who is based at the Pregnancy and Perinatology Branch, Center for Developmental Biology and Perinatal Medicine NICHD, NIH.
   Dr. Willinger is not involved at any level with the study other than in her function as Executive Secretary of the DSMC.

b) The DSMC is chaired by Dr. Christine Gleason (Specialty: Neonatology, Cerebral-vascular Physiology; Department of Pediatrics; University of Washington). Both she and all other members are independent of the study. Collectively the NRN DSMC includes experts in neonatology, maternal and fetal medicine, neurodevelopment, epidemiology, biostatistics, bioethics, high-risk infant follow-up, and clinical trials. Both the individuals and their expertise are shown in Appendix.

c) The Charter under which the DSMC operates for the NRN was drawn up in accordance with the NIH guidelines, and details the roles of the DSMC. It can be found at the password protected private gateway of the NRN as follows:

   https://neonatal.rti.org/private/pdf/Administration/PolicyNProcedures/DSMC_Charter.pdf

   The charter has been reviewed and approved by the DSMC.

   The DSMC has at least one annual in-person meeting and numerous other teleconferences, as necessary, organized by RTI, the Data Coordinating Center (DCC) for the NRN.

In brief the Committee will:

(i) Review the research protocol, review model informed consent documents, and plans for data and safety monitoring, including all proposed revisions;

(ii) Review methodology used to help maintain the confidentiality of the study data and the results of monitoring by reviewing procedures put in place by investigators to ensure confidentiality;

(iii) Monitor study design, procedures and events that will maximize the safety of the study participants and minimize the risks;
(iv) Evaluate the progress of the study, including periodic assessments of data quality and timeliness, participant recruitment, accrual and retention, participant risk versus benefit, performance of the study site(s), and other factors that may affect study outcome;
(v) Consider factors external to the study when relevant information becomes available, such as scientific or therapeutic developments that may have an impact on the safety of the participants or the ethics of the studies;
(vi) Review serious adverse event documentation and safety reports and make recommendations regarding protection of the safety of the study participants;

(See: http://www.nichd.nih.gov/funding/policies/upload/NICHD_Sample_IDMC_Charter.pdf)

d) The DSMC will be asked to critically assess the study protocol and study documents, in order to approve the study protocol, the data and safety monitoring plan, informed consent template, reporting templates for data to be presented to the board. The DSMC is fully empowered to require any other specific changes to the trial (pertaining to patient safety and trial feasibility issues) prior to any local IRB submissions and prior to any start to enrollment.

e) The DSMC deliberations will be recorded as minutes to summarize topics discussed, recommendations made; and will be signed by the board chair. All participating NRN sites and their site PIs will receive a sanitized summary of board recommendations, to be forwarded to the IRB of each participating site.

f) Any potential and actual Conflicts of Interest (COI) for board members will be reviewed and managed appropriately

2) Details of data acquisition and maintenance and protection of confidentiality

a) Database and Study Records

The data management system (DMS) recommended for the TOP study is a general purpose distributed data entry and management system developed to enable clinics to enter data from medical record extractions or other sources. This system allows quick implementation of new studies and has been successfully used for data collection for all NRN studies since 1998.

As the NRN DCC, RTI has tailored our DMS for more than 40 primary and secondary NRN studies with as many as 15 different studies active at one time. It has been used to enter, edit, and manage the data for more than 650 forms and 30,000 data items, and includes point of entry quality checks, such as range, skip pattern, and consistency checks. It provides a user-friendly data entry interface that closely resembles the paper study forms to ensure more accurate data entry.

Features of the NRN DMS

- Provides role-based security. Each user has an individual username and password with a specified data entry role. For example, only users with a certified user role can key study data.
- Allows the user to enter data in any order. Users can select any form in the system to key and can select any question to answer.
- Provides a comprehensive search capability. Users can search for a patient by entering various identifying information, such as a range of birth dates, gestational ages, birth weights.
- Generates reports. The DMS generates several different types of reports: an Adjusted Age Calculator, Field Range Report, Incomplete Records List for each protocol, and the Keyed Forms Inventory for each protocol.
- Generates data backups. Although study data are stored on two mirrored drives, the DMS provides an additional mechanism that allows the users to back up their study data files onto password protected CDs.
- Enables medical record storage. The DMS provides a mechanism for users to store and transmit various medical data files such as oximeter data files, aEEG records, to the DCC.
- Performs batch edits. In addition to range, consistency, and skip patterns checks, cross-form edits are applied to the data. Edit reports are generated and electronically submitted to the sites for resolution.
- Maintains audit trail. The DMS maintains an audit trail on any data changes made to previously completed data records.
- Builds analysis files. Using the codebook that automatically generates the data entry screens and database table structures, database tables are converted to SAS files for use by DCC statisticians.
- Provides a data transmission system. Study data are transmitted to RTI. Updates to the DMS that include new studies or version changes can also be transmitted to the research center computer via the transmission system.

Research center staff key study data into the DMS on their NRN research computers and transmit these data to RTI at least weekly. Provided by RTI, these computers are already in place at all the clinical centers. At RTI, data from the different clinical centers are combined into one dataset and are then converted to SAS data files for use by RTI study statisticians for data analysis. Patient confidentiality and privacy is maintained within the system by assigning patients a study identification number. Neither patient names nor addresses are transmitted to RTI. No other personally identifiable information (PII) or protected heath information (PHI) other than dates (i.e., dates of birth, discharge, intervention and hospital outcomes) are collected or transmitted to RTI. Once a combined dataset has been created, the data will go through additional higher level data edits and resulting queries to research center staff are posted on the secure portal of the NRN website.

The combined dataset is also utilized by the NRN capitation system to calculate monthly payments to the research centers to reimburse them for their study costs. The Steering Committee and RTI define the rules or data items that trigger payments. Additional data items will be added to the capitation system for this study.

The TOP study will be easily added as a new study module to the existing NRN DMS. Once programmed, it will go through a rigorous review that includes testing of each data item, table, form, report, and all DMS features before it is distributed to the NRN research centers. The TOP study module will be transmitted to the research centers’ computer and automatically added to the NRN DMS on the center’s NRN computer. Users can begin keying data into the study immediately after a successful transmission.

An additional module will be added to the NRN telephone randomization system for the TOP trial. This system allows clinical center staff to randomize patients at any time of the day, quickly and efficiently. Center coordinators dial a toll-free number, enter a password on the phone's keypad, provide any stratifying information (e.g., days of life, gestational age), and receive a treatment assignment through the system.

2b) Maintaining confidentiality of subject data

All named study PIs at the NRN sites, and their research coordinators are CITI certified in conduct of clinical trials, as a requirement of their participation in any NRN study. The basic rights of study participants will be respected and maintained by the investigators and by all who are involved in the collection or processing of individually identified data. NRN data collection and processing procedures are designed to protect individual rights and to comply with all applicable laws and ethical principles including confidentiality. Among the rights that must be protected are:

- the right to informed consent, which requires that prospective participants in a research project and, if needed, their family members, be provided adequate information about the potential risks, benefits, and requirements of participation so that each can make an informed decision about participation
- the right to decline, which requires that prospective participants be fully informed that their participation is completely voluntary, that they may withdraw at any time, that access to adequate health care will be provided whether or not they participate in the research, and that they may refuse to answer any question
- the right to privacy, which requires guarantees of confidentiality of information and other specific protection as specified in the Privacy Act of 1974.

2c) Data Quality Control

2c i) Internal Audit

We will continuously monitor data quality using point-of-entry quality checks in the DMS and other cross-form or longitudinal checks indicated by the investigators. We will identify errors, missing forms/items, and post center-specific reports on the private portion of the NRN website so that clinical staff can follow up. We have used this approach successfully for the NRN and other studies, producing automated routine reports on enrollment, retention, missing visits/items, and other problems, that enable us to evaluate general center performance and quickly initiate corrective actions as needed.

2c ii) Quality Assurance

The TOP DCC at RTI will operate under guidelines to ensure data accuracy, integrity, confidentiality, and security. DCC activities are governed formally by RTI Standard Operating Procedures or SOPs (see examples below) or less formally through technical operating procedures and guidance documents, which also help ensure regulatory compliance at the clinical centers.

SOPs Guiding QA Activities for TOP

DCC Role	Associated SOPs
Statisticians	• Developing Statistical Analysis Plan<br>• Creating, testing, and documenting all statistical products<br>• Writing reports

Computer Programmers	• Designing, testing, and documenting all software developed<br>• Developing and maintaining QC systems within DMS<br>• Software development lifecycle
Data Managers	• Developing Data Management Plan<br>– Checks and edits to ensure quality data entry<br>– Audit trails<br>– Data lock
Project Management	• Protocol development<br>• Human subjects protection<br>• Documenting study protocols<br>• Clinical site monitoring and training<br>• AE/SAE monitoring<br>• Essential documents management

2c iii) Participating Sites

All NRN clinical centers and RTI have IRB committees that convene on a fixed schedule every month to review protocols and associated informed consent forms and data collection procedures for all research to ensure that they are in compliance with all applicable human subject regulations. IRB approval must be granted prior to beginning any study, and study progress and procedures must be reviewed by the IRB at least annually. All staff who conduct or support research involving human subjects are required to undergo training on the protection of human subjects in research.

We will conduct targeted site visits using data audits on accrual, drop-outs, unresolved queries, and protocol violations to inform cost-efficient site monitoring decisions. Established checklists and reporting templates will be used to ensure that centers have the necessary resources; review research records and regulatory documents and observe study procedures, such as screening and recruitment. Following the site visit, the DCC will provide a written report to the center and NIH, and follow up on unresolved issues.

3) Collection and reporting of Adverse Events

a. We will use standard definitions as follows.

3a i) Unanticipated Problems

i. <u>Unanticipated Problem</u> – any incident, experience, or outcome that meets *all* of the following criteria:
   1. Is unexpected in terms of nature, severity, or frequency in relation to (a) the research risks that are described in the IRB-approved research protocol and informed consent document; Investigator's Brochure or other study documents, and (b) the characteristics of the subject population being studied; **and**
   2. Is related or possibly related to participation in the research; **and**
   3. Places subjects or others at a greater risk of harm (including physical, psychological, economic, or social harm) than was previously know or recognized.

3a ii) Adverse Event (AE)

"Defined as any reaction, side effect, or untoward event that occurs during the course of the clinical trial associated with the intervention, whether or not the event is considered related to the treatment or clinically significant. A new illness, symptom, sign or clinically significant laboratory abnormality or worsening of a pre-existing condition or abnormality is considered an Adverse Event".

AE will be monitored during the study to ensure timely detection of events that may affect the safety or continued participation of research subjects. In the TOP Trial this extremely high-risk and fragile population will each experience expected and unexpected adverse events. Adverse events and their relationship to study, severity, time of experience, expectation, actions taken to resolve the event and final outcome will be recorded as documented in the medical record, or if reported by the NICU team even before documentation. These event rates will be part of reporting the final results of the study, and for the DSMC safety monitoring.

Secondary outcomes of interest in this trial that can be compared across the two treatment groups include death; Bell Stage 2-3 necrotizing enterocolitis; periventricular leukomalacia or echodense lesions or ventriculomegaly; apnea mandating either caffeine or respiratory support; bronchopulmonary dysplasia; retinopathy of prematurity requiring intervention; and each component of the primary outcome (available only after the 18- to 22-month follow-up visit). Rates of these events, historically observed among similar extremely low gestation/birth weight infants, will be provided to the DSMC for comparison. The set of common, serious neonatal morbidities as defined in the NRN Generic Data Base (GDB) will also be collected. These include data on in-hospital growth, the incidence and severity of intraventricular hemorrhage, seizures, patent ductus arteriosus (PDA) and its treatment, nosocomial sepsis (and organisms), hearing impairment, and pneumothorax.

3a iii) Serious Adverse Events (SAE)

Definitions in this study are listed below. Based on the premature infant population studied, the SAE categories of events that result in congenital anomaly/birth defect or require intervention to prevent permanent impairment or damage (Devices) do not apply when defining events.

An adverse event or suspected adverse reaction is considered serious if in the review of the investigator or the sponsor, it results in any of the following:

Death of Subject	An event that results in the death of a subject.
Life-Threatening	An event that, in the opinion of the investigator, would have resulted in immediate fatality if medical intervention had not been taken. This does not include an event that would have been fatal if it had occurred in a more severe form.

Prolongation of Hospitalization	An event that prolongs the subject's hospital stay.
Results in persistent or significant disability/incapacity	An adverse event that may result in a substantial disruption of the ability to conduct normal life functions, i.e., the adverse event resulted in a significant, persistent or permanent change, impairment, damage or disruption in the infant's body function/structure, physical activities and/or quality of life.
Important Medical Event Requiring Medical or Surgical Intervention to Prevent Serious Outcome	An important medical event that may not be immediately life-threatening or result in death or hospitalization, but based on medical judgment may jeopardize the subject and may require medical or surgical intervention to prevent any of the outcomes listed above (i.e., death of subject, life-threatening, prolongation of hospitalization). An example of such events would be an allergic bronchospasm requiring intensive treatment.

Expedited Serious Adverse Events (SAE)

The sponsor NICHD will determine if individual serious adverse events a) meet the criteria for expedited reporting in accordance with 21CFR 312.32 IND safety reporting and b) are promptly reported to the DSMBCand NHLBI.

ii) Unexpected Adverse Event

"Any adverse event, the specificity or severity of which is not listed in the study protocol, product inserts, or informed consent document"

iii) <u>Attribution</u> – the determination of whether an adverse event is related to a medical treatment or procedure:

This will be performed by the site PI and study team, according to the basis of the below classification:

1. Definitely
2. Probably
3. Possibly
4. Unlikely
5. Unrelated

This will then be shared with all the bodies listed below under 3 (b)

3.b) Adverse Event Management and Reporting

If an adverse event meets any of the criteria listed aboveof Serious (3a i), Unexpected (3 a ii) and at least possibley attributable (3a iii) to the study therapy or intervention, it will be reported by study team at the recruiting site to the NICHD, the Data Coordinating Center (DCC)

RTI, and the local IRB (per site specific IRB procedures) as a serious adverse event via the Medwatch Safety Reporitng Form 3500A.

These eventsis must be performed within 24 hours of the site being made aware of the SAE. NICHD will report the event to the NHLBI. A Medwatch form 3500A will be completed. Adverse Event /Serious Adverse Event forms will be completed by site research staff (investigators and/or coordinators) for each reportable (as defined by protocol) adverse event that infants may experience from the time of randomization through the predefined intervention and/or events monitoring period. Events will be captured documented by clinical site staff using protocl caase report forms (CRF), reviewed by the site investigators for accuracy and completeness then entered into the specified Data Management System (DMS) form(s) as instructed in the manual of procedures that will be developed for this trial. Specific study defined events of interest, death and any other event that could be classified as serious, unexpected and potentially attributable to study are to be reported using FDA form 3500A (MedWatch) by fax or email to the RTI DCC Coordinator and NHLBI and NICHD Program Scientists within one business day of discovery. The Program Scientist at NICHD and/or the DCC will determine whether expedited DSMC reviews are necessary. The DSMC can recommend further action and the the the DCC is responsible for notification to the local IRB, NICHD and NHLBI. NHLBI and NICHD may request the DCC notify required parties through established communication mechanisms via technical memos.

Reporting to the Food and Drug Administration will not be required for this trial as it involves no new drug or device intervention mandating an IND/IDE.

iv) Amendments to the study protocol: process of review and implementation

a. It is not expected that we will encounter any major modifications to the protocol, as the most significant portions of this have been already pre-tested within the PINT trial. However it is always possible that these may be needed. In that event, the Principal Investigators of the trial (HK, EB, AD) will be jointly responsible for the coordination and development of all protocol amendments in collaboration with the study subcommittees, NICHD and NHLBI as necessary. Dissemination of this information to the participating centers will be managed by the NRN DCC.
b. Any changes to the protocol or consent will be made in the form of an amendment and must be approved prior to implementation and this process will take the following shape:

The initial assessment of the need to have a study design-protocol change will be first assessed by the PIs (HK, ED, AD). If a substantive need is felt, these three will convene a sub-committee meeting. It will then be sent to the Steering Committee of the NRN. Following substantive discussion there, if the collective decision is that there needs amendment, this will be drafted and sent to the NRN DSMC for approval. Once the DSMC has approved the changes, they will be sent to each PI of the each site, who will be instructed to ask their local IRB to vet and pass the amendment. Only after each IRB has agreed to an amendment will the changes written there, be implemented to the study as it pertains to human subjects.

v) Benefits and Risks of the Study to enrolled subjects

We avoid stepping beyond current practice boundaries of thresholds of hemoglobin that would trigger a transfusion by assuring broad buy-in of NRN clinicians and a transfusion algorithm that was developed by consensus. This approach avoids the dilemmas sometimes faced by other trials (Deans KJ 2007). This open trial, allows clinicians to bypass the study transfusion algorithm in circumstances where an infant's condition

warrants acute transfusion, but then revert to the algorithm thereafter. The potential risks to subjects participating in this study or their parents are categorized in this study as 'minimal risk'. The trial maneuvers – other than randomization – are all within the range of current clinical practice and well within the likely variation of normal clinical practice. We do not mandate any additional blood sampling nor specific route of sampling of blood, thus not departing from the standard practice of the NICU. The informed consent document and patient information materials will be thoroughly reviewed with the parents prior to their completing the written informed consent. Families will be given ample opportunity to ask questions regarding alternative options.

6. Data analysis plan

***Main Study*:**

Design The primary objective of the TOP trial is to assess the difference in the primary outcome of survival without significant NDI (or, equivalently, death or NDI) at 22-26 months adjusted age in the two treatment arms. This outcome is similar to that used in the PINT trial, which found rates of death or neurosensory impairment at 18 months of 45.2% in the low trigger group and 38.5% in the high trigger group. Based on these findings, we believe that an absolute difference of 7% is the minimal clinically significant difference we would wish to detect in the rate of death or NDI. We considered several other factors in estimating the sample size for this study. First, we calculated the rate of death or NDI among infants born at NRN centers in 2005-2008 with GA 22-26 weeks and birth weight <1000 g who survived to 12 hours, which was 52%. However, we recognized that this population differed somewhat from that of the TOP trial, which proposes to enroll infants less than 29 weeks GA. In addition, the current NRN definition of NDI incorporates only the cognitive composite score of the Bayley III, while future definitions will likely also involve the Bayley III motor scales, which have been collected in the Network only since January 2010. Thus, we recognized the limitations of estimating our sample size based only on current NRN data. At the same time, however, we were aware that the most conservative sample size estimate would result from an event rate of 50%, which current NRN data indicated was not implausible in our population. This led us to choose a conservative estimate based on assumed outcome rates of 53.5% and 46.5% (i.e., centered around an overall event rate of 50%) in the two treatment groups. With a two-tailed alpha of 0.05 and 80% power, the initial sample size estimate was 1,658 infants. The sample size was additionally inflated by 10% to account for loss to follow up at 2-26 months, yielding a final sample size estimate of 1,824.

Although the sample size calculations described above were based on the primary outcome, we will have 80% power with a two-tailed alpha of 0.05 to detect the following differences in important secondary outcomes (rates in the low threshold group are based on findings of the PINT trial): any transfusion 89.0% in the low threshold group (LOW) vs. 92.9% in the high group (HI); NEC 8.5% in LOW vs. 5.0% in HI; brain injury (PVL, echodense lesions or ventriculomegaly) on latest ultrasound (US) to 36 weeks 18.5% in LOW vs. 24.6% in HI; death before discharge 21.5% in LOW vs. 16.1% in HI; combined death, severe ROP, BPD, or US brain injury 74.0% in LOW vs. 67.7% in HI.

Analysis All analyses will be conducted on an intention-to-treat basis, with the caveat that infants surviving to discharge, but lost to follow-up at 22-26 months, will be excluded from the primary analysis and considered irrevocably missing. We will, however, assess the impact of outcomes lost to follow-up on our results by performing sensitivity analyses that assume all positive or all negative outcomes for those with missing data. We will also conduct bivariate analyses to compare the in-hospital characteristics of children lost to follow-up versus those with

assessment of the primary outcome. Note that, based on previous NRN trials in similar populations, we expect the follow-up rate among survivors to be above 90%.

All analyses will be adjusted for design effects: stratification by center and birth weight group. The primary outcome of death or NDI at 22-26 months adjusted age will be analyzed using robust Poisson regression. Adjusted relative risks and 95% confidence intervals will be reported. Secondary analyses of the primary outcome may adjust for additional baseline covariates such as SNAPPE scores at NICU admission and socioeconomic status. The consistency of the treatment effect over birth weight strata and gender, and treatment heterogeneity across the different centers, will be investigated.

Secondary outcomes including comorbidities such as NEC, ROP, BPD, and NDI among survivors at follow up, as well as other binary outcomes, will be analyzed using robust Poisson regression, as described above. In addition to the stratification variables, models may also be adjusted for covariates known to be associated with the secondary outcomes in this population. For example, both gender and socioeconomic status have been implicated as crucial confounders for cognitive outcomes among survivors at follow up (53); thus, it may be appropriate to include them as covariates in models for such outcomes. Number of transfusions will be analyzed using Poisson regression. Other secondary outcomes, such as time to regain birth weight and time to full feeds, will be analyzed using Cox proportional hazards survival regression. Although the basic Cox model assumes proportional hazards between groups, if this assumption is found to be violated in our data, time-varying covariates can be added to the models to allow for changes in the proportion over time. Weekly measures of growth (weight, length, head circumference) will be analyzed using longitudinal models to test whether growth trajectories differ between treatment groups. All models incorporating the treatment effect will be adjusted for the stratification variables. All secondary outcomes analyses will be considered exploratory because the trial is not primarily powered to detect these associations.

6b) Interim Analysis and 6c) Stopping Rules

Interim analyses for safety will be conducted at four time points during the study, after

250, 500, 1000 and 1500 babies enrolled have reached status, as defined in the NRN (death, discharge, transfer, or 120 days of age, whichever occurs earlier). Rates of death and other prespecified adverse events (AEs), including NEC and adverse head US findings, will be compared between the two treatment groups. Analyses of AEs that were not prespecified may be added during the course of the study as deemed appropriate by the DSMC or the Steering Committee, in accordance with any new findings reported in the literature that may be of relevance to this trial.

The statistical significance of interim safety analyses will be based on O'Brien Fleming bounds calculated with a Lan-DeMets spending function to preserve an overall Type-I error rate of 0.05 for a composite safety outcome of death or NEC or adverse head US findings, and tests that exceed the boundary will be considered as evidence of a statistically significant difference between treatment groups. In the event that a test approaches significance using these stopping rules, the DCC will report to the DSMC Chair who can then take appropriate action. To ensure that Drs. Das and Brambilla remain blinded to evolving results, they will not be directly involved in these analyses. The results of the interim analyses will thus remain confidential, with only the trial statistician having access to unblinded data.

We recommend that the DSMC meet formally to review safety and other aspects of accruing data (such as enrollment, data completeness and protocol compliance) three times during the study, at roughly 25% , 50% and 75% of subject accrual). Formal DSMC reports will be prepared by the DCC for these meetings. These reports will include the following information:

- Brief summary of the trial design, including primary and secondary hypotheses and outcomes, study population, inclusion and exclusion criteria, recruitment, screening, randomization and study intervention procedures, and statistical considerations for trial design and analysis
- Interim monitoring plan
- Enrollment, including screening, consent, randomization and study exit
- Completeness of data and edit queries. Baseline study population characteristics, overall and by treatment group
- Treatment compliance
- Primary efficacy outcomes by treatment group, if at least 25% available
- Secondary efficacy outcomes by treatment group, if at least 25% available
- Safety outcomes, including death, by treatment group
- Protocol deviations and violations, overall and by treatment group.

These DSMC reports will be blinded, with treatments labeled as group A and group B. As per the NRN DSMC Charter, the DSMC may, however, request to be unblinded to perform their duties. If the DSMC recommends modification or cessation of the study protocol due to safety concerns, NIH will make the final determination.

Because the enrollment period for this study is less than 3 years and the NDI component of the primary outcome will not be obtained until 22-26 months adjusted age, we do not plan to conduct formal interim analyses for efficacy. However, when the DSMC meets for formal safety analyses, members will be presented with, and may choose to make recommendations on the basis of, data regarding recruitment and retention, protocol adherence (including compliance with the intervention algorithm), and other factors that relate to the scientific validity of the trial. We also anticipate that towards the end of trial recruitment, some primary outcome data may be available, and such data will be included in the DSMC report for every 500 babies with primary outcome accrual if it is available on at least 25% of the total target sample size of 1824. O'Brien Fleming stopping bounds for efficacy, calculated with a Lan-DeMets spending function to preserve an overall Type-I error rate of 0.05 for the primary outcome will be used.
