# Supplementary material for: Tissue Oxygenation Changes After Transfusion and Outcomes in Preterm Infants: A Secondary Near-Infrared Spectroscopy Study of the Transfusion of Prematures Randomized Clinical Trial (TOP NIRS)
Source: JAMA Netw Open. 2023 Sep 21;6(9):e2334889. doi: 10.1001/jamanetworkopen.2023.34889 (PMC10514737; doi:10.1001/jamanetworkopen.2023.34889)
Supplement: Supplement 2. — eTable 1. Hemoglobin Transfusion Thresholds eTable 2. CART Variables eFigure. Flow Diagram [file jamanetwopen-e2334889-s002.pdf]

Supplemental Online Content

Chock VY, Kirpalani H, Bell EF, et al; Eunice Kennedy Shriver National Institute of Child Health and Human Development Neonatal Research Network. Tissue oxygenation changes after transfusion and outcomes in preterm infants: a secondary near-infrared spectroscopy study of the Transfusion of Prematures randomized clinical trial (TOP NIRS). *JAMA Netw Open*. 2023;6(9):e2334889. doi:10.1001/jamanetworkopen.2023.34889

eTable 1. Hemoglobin Transfusion Thresholds

eTable 2. CART Variables

eFigure. Flow Diagram

This supplemental material has been provided by the authors to give readers additional information about their work.

© 2023 Chock VY et al. *JAMA Network Open.*

eTable 1. Hemoglobin Transfusion Thresholds

	High Hgb threshold (g/dl)		Low Hgb threshold (g/dl)	
**Postnatal Age**	**Respiratory support***	**No respiratory support**	**Respiratory support***	**No respiratory support**
Week 1	13.0	12.0	11.0	10.0
Week 2	12.5	11.0	10.0	8.5
Week ≥3	11.0	10.0	8.5	7.0

*Respiratory support defined as mechanical ventilation, continuous positive airway pressure, fraction of inspired oxygen >0.35, or nasal cannula flow ≥ 1 liter per minute

© 2023 Chock VY et al. *JAMA Network Open.*

eTable 2. Variables used for CART analysis to predict death or NDI*

Category	Variable included for model development
Demographics	
	Gestation (in completed weeks)
	Sex
	Birth weight (g)
	Singleton
	Antenatal steroids given
	Private insurance
	Maternal education level[a]
Clinical factors	
	Days of ventilation within first 28 days
	Total days on ventilation
	Days of parenteral alimentation
	Necrotizing enterocolitis[b]
	Abnormal head ultrasound[c]
	Any sepsis (early or late onset)
	Ventriculomegaly[d]
NIRS measures	
	Pre-transfusion mean Csat[e]
	Pre-transfusion mean cFTOE[e]
	Delta Csat[e]
	Delta cFTOE[e]
	Csat <50% in first 72 hours
	% time Csat <50% in first 72 hours
	Total transfusions with mean pre-transfusion Csat <50%
	Total transfusions with mean post-transfusion Csat <50%
Hemoglobin or transfusion factors	
	Hemoglobin transfusion threshold (high vs. low)
	Total transfusions
	Total transfusions triggered per protocol
	Total transfusions with NIRS measures available
	Hemoglobin at randomization[e]
	Hemoglobin triggering transfusion per protocol[e]
	Mean hemoglobin over entire study period[e]

*The importance of each predictor was set as the attributable change in the residual sum of squares (RSS) over all node splits of the tree. The entropy method was used to select the variables for the tree, the cost-complexity method was used to prune it, and each leaf was restricted to a minimum number of 10 observations. NIRS = near-infrared spectroscopy; Csat = cerebral saturation; cFTOE = cerebral fractional tissue oxygen extraction; [a]Three-level categorical variable; [b]Modified Bell stage II or higher; [c]Severe intraventricular hemorrhage (grade 3 or 4), cerebellar hemorrhage, intraparenchymal echodensity, periventricular leukomalacia, or ventriculomegaly on cranial ultrasound obtained prior to discharge; [d]Seen if concurrent intraventricular hemorrhage grade 2 or higher; [e]Variables summarized at the infant level over the entire study period and also separately for the following time periods: first postnatal week, after the first postnatal week, up to 28 days, after 28 days, from 8-28 days

© 2023 Chock VY et al. *JAMA Network Open.*

eFigure. Flow Diagram of Enrollment

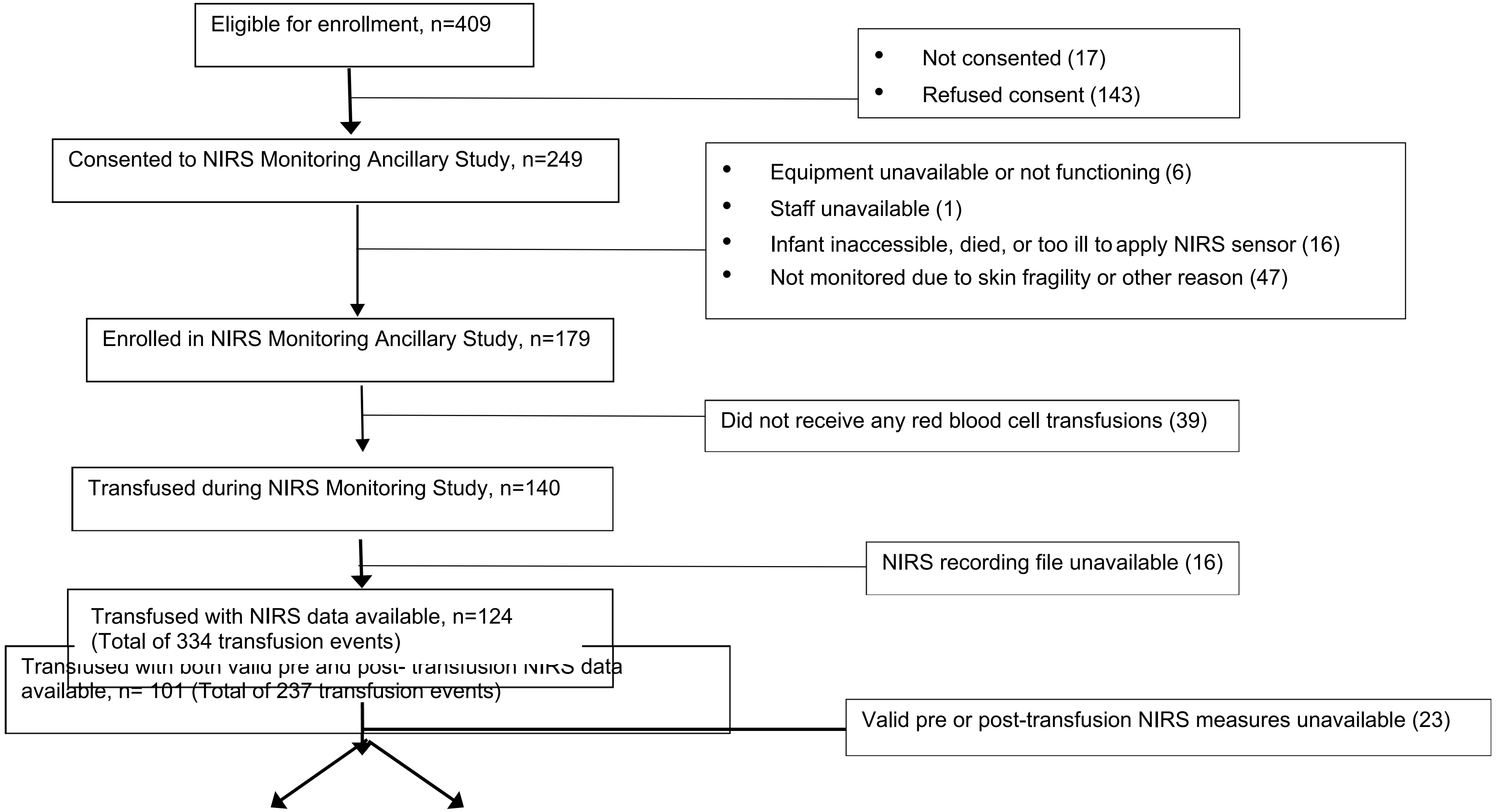

© 2023 Chock VY et al. *JAMA Network Open.*

Transfused over the first 28 days
(primary analysis sample, n=80 with 164
transfusion events)

Data available for death or NDI
(secondary analysis sample, n=97)

© 2023 Chock VY et al. *JAMA Network Open.*
