# Supplementary material for: Tissue Oxygenation Changes After Transfusion and Outcomes in Preterm Infants: A Secondary Near-Infrared Spectroscopy Study of the Transfusion of Prematures Randomized Clinical Trial (TOP NIRS)
Source: JAMA Netw Open. 2023 Sep 21;6(9):e2334889. doi: 10.1001/jamanetworkopen.2023.34889 (PMC10514737; doi:10.1001/jamanetworkopen.2023.34889)
Supplement: Supplement 3. — Eunice Kennedy Shriver National Institute of Child Health and Human Development Neonatal Research Network Investigators [file jamanetwopen-e2334889-s003.pdf]

Supplemental Online Content: Nonauthor Collaborators
*First name, last name, and suffix (if applicable) are required and will appear in PubMed.

*Group Name(s): Eunice Kennedy Shriver National Institute of Child Health and Human Development Neonatal Research Network							
***First Name and Middle Initial(s)**	***Last Name**	***Suffix (eg, Jr, III)**	Academic Degrees	Institution	Location (city, state/province, country)	Role or Contribution, eg, chair, principal investigator	Group (if more than 1 Group listed in the byline) and/or Subgroup (eg, Steering Committee)
Sharon L.	Wright		MT (ASCP)	McGovern Medical School at The University of Texas Health Science Center at Houston, Children's Memorial Hermann Hospital, and Memorial Hermann Southwest Hospital	Houston, TX	Non-Author Contributor	
Robin S.	Roberts		Btech Mtech	McMaster University	Hamilton, Ontario, Canada	Non-Author Contributor	
Traci H.	Mondoro		PhD	National Heart, Lung, and Blood Institute	Bethesda, MD	Non-Author Contributor	
Catherine	Levy		MHA BSN RN	National Heart, Lung, and Blood Institute	Bethesda, MD	Non-Author Contributor	
Leif D.	Nelin		MD	Nationwide Children’s Hospital, Abigail Wexner Research Institute at Nationwide Children’s Hospital, Center for Perinatal Research, The Ohio State University Wexner Medical Center, and Riverside Methodist Hospital	Columbus, OH	Non-Author Contributor	
Nathalie L.	Maitre		MD PhD	Nationwide Children’s Hospital, Abigail Wexner Research Institute at Nationwide Children’s Hospital, Center for Perinatal Research, The Ohio State University Wexner Medical Center, and Riverside Methodist Hospital	Columbus, OH	Non-Author Contributor	

Supplemental Online Content: Nonauthor Collaborators
*First name, last name, and suffix (if applicable) are required and will appear in PubMed.

***First Name and Middle Initial(s)**	***Last Name**	***Suffix (eg, Jr, III)**	Academic Degrees	Institution	Location (city, state/province, country)	Role or Contribution, eg, chair, principal investigator	Group (if more than 1 Group listed in the byline) and/or Subgroup (eg, Steering Committee)
Sudarshan R.	Jadcherla		MD	Nationwide Children’s Hospital, Abigail Wexner Research Institute at Nationwide Children’s Hospital, Center for Perinatal Research, The Ohio State University Wexner Medical Center, and Riverside Methodist Hospital	Columbus, OH	Non-Author Contributor	
Patricia	Luzader		RN	Nationwide Children’s Hospital, Abigail Wexner Research Institute at Nationwide Children’s Hospital, Center for Perinatal Research, The Ohio State University Wexner Medical Center, and Riverside Methodist Hospital	Columbus, OH	Non-Author Contributor	
Christine A.	Fortney		PhD RN	Nationwide Children’s Hospital, Abigail Wexner Research Institute at Nationwide Children’s Hospital, Center for Perinatal Research, The Ohio State University Wexner Medical Center, and Riverside Methodist Hospital	Columbus, OH	Non-Author Contributor	
Julie	Gutentag		RN BSN	Nationwide Children’s Hospital, Abigail Wexner Research Institute at Nationwide Children’s Hospital, Center for Perinatal Research, The Ohio State University Wexner Medical Center, and Riverside Methodist Hospital	Columbus, OH	Non-Author Contributor	

Supplemental Online Content: Nonauthor Collaborators
*First name, last name, and suffix (if applicable) are required and will appear in PubMed.

***First Name and Middle Initial(s)**	***Last Name**	***Suffix (eg, Jr, III)**	Academic Degrees	Institution	Location (city, state/province, country)	Role or Contribution, eg, chair, principal investigator	Group (if more than 1 Group listed in the byline) and/or Subgroup (eg, Steering Committee)
Christopher J.	Timan		MD	Nationwide Children's Hospital, Abigail Wexner Research Institute at Nationwide Children's Hospital, Center for Perinatal Research, The Ohio State University Wexner Medical Center, and Riverside Methodist Hospital	Columbus, OH	Non-Author Contributor	
Kristi	Small		BS	Nationwide Children's Hospital, Abigail Wexner Research Institute at Nationwide Children's Hospital, Center for Perinatal Research, The Ohio State University Wexner Medical Center, and Riverside Methodist Hospital	Columbus, OH	Non-Author Contributor	
Rox Ann	Sullivan		RN BSN	Nationwide Children's Hospital, Abigail Wexner Research Institute at Nationwide Children's Hospital, Center for Perinatal Research, The Ohio State University Wexner Medical Center, and Riverside Methodist Hospital	Columbus, OH	Non-Author Contributor	
Lina	Yoseff-Salameh		MD	Nationwide Children's Hospital, Abigail Wexner Research Institute at Nationwide Children's Hospital, Center for Perinatal Research, The Ohio State University Wexner Medical Center, and Riverside Methodist Hospital	Columbus, OH	Non-Author Contributor	

Supplemental Online Content: Nonauthor Collaborators
*First name, last name, and suffix (if applicable) are required and will appear in PubMed.

***First Name and Middle Initial(s)**	***Last Name**	***Suffix (eg, Jr, III)**	Academic Degrees	Institution	Location (city, state/province, country)	Role or Contribution, eg, chair, principal investigator	Group (if more than 1 Group listed in the byline) and/or Subgroup (eg, Steering Committee)
Jacqueline	McCool			Nationwide Children’s Hospital, Abigail Wexner Research Institute at Nationwide Children’s Hospital, Center for Perinatal Research, The Ohio State University Wexner Medical Center, and Riverside Methodist Hospital	Columbus, OH	Non-Author Contributor	
Melanie	Stein		RRT BBA	Nationwide Children’s Hospital, Abigail Wexner Research Institute at Nationwide Children’s Hospital, Center for Perinatal Research, The Ohio State University Wexner Medical Center, and Riverside Methodist Hospital	Columbus, OH	Non-Author Contributor	
Erin	Fearns			Nationwide Children’s Hospital, Abigail Wexner Research Institute at Nationwide Children’s Hospital, Center for Perinatal Research, The Ohio State University Wexner Medical Center, and Riverside Methodist Hospital	Columbus, OH	Non-Author Contributor	
Aubrey	Fowler		BS	Nationwide Children’s Hospital, Abigail Wexner Research Institute at Nationwide Children’s Hospital, Center for Perinatal Research, The Ohio State University Wexner Medical Center, and Riverside Methodist Hospital	Columbus, OH	Non-Author Contributor	

Supplemental Online Content: Nonauthor Collaborators
*First name, last name, and suffix (if applicable) are required and will appear in PubMed.

***First Name and Middle Initial(s)**	***Last Name**	***Suffix (eg, Jr, III)**	Academic Degrees	Institution	Location (city, state/province, country)	Role or Contribution, eg, chair, principal investigator	Group (if more than 1 Group listed in the byline) and/or Subgroup (eg, Steering Committee)
Jennifer	Grothause		RN BSN	Nationwide Children's Hospital, Abigail Wexner Research Institute at Nationwide Children's Hospital, Center for Perinatal Research, The Ohio State University Wexner Medical Center, and Riverside Methodist Hospital	Columbus, OH	Non-Author Contributor	
Stephanie	Burkhardt		BS MPH	Nationwide Children's Hospital, Abigail Wexner Research Institute at Nationwide Children's Hospital, Center for Perinatal Research, The Ohio State University Wexner Medical Center, and Riverside Methodist Hospital	Columbus, OH	Non-Author Contributor	
Jessica	Purnell		BS CCRC	Nationwide Children's Hospital, Abigail Wexner Research Institute at Nationwide Children's Hospital, Center for Perinatal Research, The Ohio State University Wexner Medical Center, and Riverside Methodist Hospital	Columbus, OH	Non-Author Contributor	
Mary Ann	Nelin		MD	Nationwide Children's Hospital, Abigail Wexner Research Institute at Nationwide Children's Hospital, Center for Perinatal Research, The Ohio State University Wexner Medical Center, and Riverside Methodist Hospital	Columbus, OH	Non-Author Contributor	

Supplemental Online Content: Nonauthor Collaborators
*First name, last name, and suffix (if applicable) are required and will appear in PubMed.

***First Name and Middle Initial(s)**	***Last Name**	***Suffix (eg, Jr, III)**	Academic Degrees	Institution	Location (city, state/province, country)	Role or Contribution, eg, chair, principal investigator	Group (if more than 1 Group listed in the byline) and/or Subgroup (eg, Steering Committee)
Helen	Carey		PT DHSc PCS	Nationwide Children’s Hospital, Abigail Wexner Research Institute at Nationwide Children’s Hospital, Center for Perinatal Research, The Ohio State University Wexner Medical Center, and Riverside Methodist Hospital	Columbus, OH	Non-Author Contributor	
Lindsay	Pietruszewski		PT DPT	Nationwide Children’s Hospital, Abigail Wexner Research Institute at Nationwide Children’s Hospital, Center for Perinatal Research, The Ohio State University Wexner Medical Center, and Riverside Methodist Hospital	Columbus, OH	Non-Author Contributor	
Margaret	Sullivan		BS	Nationwide Children’s Hospital, Abigail Wexner Research Institute at Nationwide Children’s Hospital, Center for Perinatal Research, The Ohio State University Wexner Medical Center, and Riverside Methodist Hospital	Columbus, OH	Non-Author Contributor	
Julie	Shadd		BS RD LD	Nationwide Children’s Hospital, Abigail Wexner Research Institute at Nationwide Children’s Hospital, Center for Perinatal Research, The Ohio State University Wexner Medical Center, and Riverside Methodist Hospital	Columbus, OH	Non-Author Contributor	

Supplemental Online Content: Nonauthor Collaborators
*First name, last name, and suffix (if applicable) are required and will appear in PubMed.

***First Name and Middle Initial(s)**	***Last Name**	***Suffix (eg, Jr, III)**	Academic Degrees	Institution	Location (city, state/province, country)	Role or Contribution, eg, chair, principal investigator	Group (if more than 1 Group listed in the byline) and/or Subgroup (eg, Steering Committee)
Jennifer	Notestine		RN	Nationwide Children's Hospital, Abigail Wexner Research Institute at Nationwide Children's Hospital, Center for Perinatal Research, The Ohio State University Wexner Medical Center, and Riverside Methodist Hospital	Columbus, OH	Non-Author Contributor	
Cole	Hague		BA MS	Nationwide Children's Hospital, Abigail Wexner Research Institute at Nationwide Children's Hospital, Center for Perinatal Research, The Ohio State University Wexner Medical Center, and Riverside Methodist Hospital	Columbus, OH	Non-Author Contributor	
Erna	Clark		BA	Nationwide Children's Hospital, Abigail Wexner Research Institute at Nationwide Children's Hospital, Center for Perinatal Research, The Ohio State University Wexner Medical Center, and Riverside Methodist Hospital	Columbus, OH	Non-Author Contributor	
Michelle	Chan		BS	Nationwide Children's Hospital, Abigail Wexner Research Institute at Nationwide Children's Hospital, Center for Perinatal Research, The Ohio State University Wexner Medical Center, and Riverside Methodist Hospital	Columbus, OH	Non-Author Contributor	

*First name, last name, and suffix (if applicable) are required and will appear in PubMed.

***First Name and Middle Initial(s)**	***Last Name**	***Suffix (eg, Jr, III)**	Academic Degrees	Institution	Location (city, state/province, country)	Role or Contribution, eg, chair, principal investigator	Group (if more than 1 Group listed in the byline) and/or Subgroup (eg, Steering Committee)
Courtney	Park		RN	Nationwide Children's Hospital, Abigail Wexner Research Institute at Nationwide Children's Hospital, Center for Perinatal Research, The Ohio State University Wexner Medical Center, and Riverside Methodist Hospital	Columbus, OH	Non-Author Contributor	
Hallie	Baugher		BS MSN	Nationwide Children's Hospital, Abigail Wexner Research Institute at Nationwide Children's Hospital, Center for Perinatal Research, The Ohio State University Wexner Medical Center, and Riverside Methodist Hospital	Columbus, OH	Non-Author Contributor	
Demi R.	Beckford		MHS	Nationwide Children's Hospital, Abigail Wexner Research Institute at Nationwide Children's Hospital, Center for Perinatal Research, The Ohio State University Wexner Medical Center, and Riverside Methodist Hospital	Columbus, OH	Non-Author Contributor	
Bethany	Miller		RN BSN	Nationwide Children's Hospital, Abigail Wexner Research Institute at Nationwide Children's Hospital, Center for Perinatal Research, The Ohio State University Wexner Medical Center, and Riverside Methodist Hospital	Columbus, OH	Non-Author Contributor	

Supplemental Online Content: Nonauthor Collaborators
*First name, last name, and suffix (if applicable) are required and will appear in PubMed.

***First Name and Middle Initial(s)**	***Last Name**	***Suffix (eg, Jr, III)**	Academic Degrees	Institution	Location (city, state/province, country)	Role or Contribution, eg, chair, principal investigator	Group (if more than 1 Group listed in the byline) and/or Subgroup (eg, Steering Committee)
Laura	Marzec		MD	Nationwide Children's Hospital, Abigail Wexner Research Institute at Nationwide Children's Hospital, Center for Perinatal Research, The Ohio State University Wexner Medical Center, and Riverside Methodist Hospital	Columbus, OH	Non-Author Contributor	
Kyrstin	Warnimont		BS	Nationwide Children's Hospital, Abigail Wexner Research Institute at Nationwide Children's Hospital, Center for Perinatal Research, The Ohio State University Wexner Medical Center, and Riverside Methodist Hospital	Columbus, OH	Non-Author Contributor	
Carla M.	Bann		PhD	RTI International	Research Triangle Park, NC	Non-Author Contributor	
Marie G.	Gantz		PhD	RTI International	Research Triangle Park, NC	Non-Author Contributor	
Jeanette	O'Donnell Auman		BS	RTI International	Research Triangle Park, NC	Non-Author Contributor	
Annie	Bayard		BS	RTI International	Research Triangle Park, NC	Non-Author Contributor	
Donald J.	Brambilla		PhD	RTI International	Research Triangle Park, NC	Non-Author Contributor	
Margaret M.	Crawford		BS CCRP	RTI International	Research Triangle Park, NC	Non-Author Contributor	
Jenna	Gabrio		MPH	RTI International	Research Triangle Park, NC	Non-Author Contributor	
Jamie E.	Newman		PhD MPH	RTI International	Research Triangle Park, NC	Non-Author Contributor	

Supplemental Online Content: Nonauthor Collaborators
*First name, last name, and suffix (if applicable) are required and will appear in PubMed.

***First Name and Middle Initial(s)**	***Last Name**	***Suffix (eg, Jr, III)**	Academic Degrees	Institution	Location (city, state/province, country)	Role or Contribution, eg, chair, principal investigator	Group (if more than 1 Group listed in the byline) and/or Subgroup (eg, Steering Committee)
Carolyn M.	Petrie Huitema		MS	RTI International	Research Triangle Park, NC	Non-Author Contributor	
David	Leblond		BS	RTI International	Research Triangle Park, NC	Non-Author Contributor	
Dennis	Wallace		PhD	RTI International	Research Triangle Park, NC	Non-Author Contributor	
Kristin M.	Zaterka-Baxter		RN BSN	RTI International	Research Triangle Park, NC	Non-Author Contributor	
David K.	Stevenson		MD	Stanford University, El Camino Hospital, and Lucile Packard Children's Hospital	Stanford, Mountain View, and Palo Alto, CA	Non-Author Contributor	
Barbara	Bentley		PsychD MSEd	Stanford University, El Camino Hospital, and Lucile Packard Children's Hospital	Stanford, Mountain View, and Palo Alto, CA	Non-Author Contributor	
Maria Elena	DeAnda		PhD	Stanford University, El Camino Hospital, and Lucile Packard Children's Hospital	Stanford, Mountain View, and Palo Alto, CA	Non-Author Contributor	
Anne M.	DeBattista		RN PNNP PhD	Stanford University, El Camino Hospital, and Lucile Packard Children's Hospital	Stanford, Mountain View, and Palo Alto, CA	Non-Author Contributor	
Beth	Earhart		PhD	Stanford University, El Camino Hospital, and Lucile Packard Children's Hospital	Stanford, Mountain View, and Palo Alto, CA	Non-Author Contributor	
Lynne C.	Huffman		MD	Stanford University, El Camino Hospital, and Lucile Packard Children's Hospital	Stanford, Mountain View, and Palo Alto, CA	Non-Author Contributor	
Casey E.	Kruger		PhD	Stanford University, El Camino Hospital, and Lucile Packard Children's Hospital	Stanford, Mountain View, and Palo Alto, CA	Non-Author Contributor	

Supplemental Online Content: Nonauthor Collaborators
*First name, last name, and suffix (if applicable) are required and will appear in PubMed.

***First Name and Middle Initial(s)**	***Last Name**	***Suffix (eg, Jr, III)**	Academic Degrees	Institution	Location (city, state/province, country)	Role or Contribution, eg, chair, principal investigator	Group (if more than 1 Group listed in the byline) and/or Subgroup (eg, Steering Committee)
Ryan E.	Lucash		PhD	Stanford University, El Camino Hospital, and Lucile Packard Children's Hospital	Stanford, Mountain View, and Palo Alto, CA	Non-Author Contributor	
Melinda S.	Proud		RCP	Stanford University, El Camino Hospital, and Lucile Packard Children's Hospital	Stanford, Mountain View, and Palo Alto, CA	Non-Author Contributor	
Elizabeth N.	Reichert		MA CCRC	Stanford University, El Camino Hospital, and Lucile Packard Children's Hospital	Stanford, Mountain View, and Palo Alto, CA	Non-Author Contributor	
Dharshi	Sivakumar		MD	Stanford University, El Camino Hospital, and Lucile Packard Children's Hospital	Stanford, Mountain View, and Palo Alto, CA	Non-Author Contributor	
Heather	Taylor		PhD	Stanford University, El Camino Hospital, and Lucile Packard Children's Hospital	Stanford, Mountain View, and Palo Alto, CA	Non-Author Contributor	
Hali E.	Weiss		MD	Stanford University, El Camino Hospital, and Lucile Packard Children's Hospital	Stanford, Mountain View, and Palo Alto, CA	Non-Author Contributor	
Namasivayam	Ambalavanan		MD	University of Alabama at Birmingham Health System and Children's Hospital of Alabama	Birmingham, AL	Non-Author Contributor	
Monica V.	Collins		RN BSN MaEd	University of Alabama at Birmingham Health System and Children's Hospital of Alabama	Birmingham, AL	Non-Author Contributor	
Shirley S.	Cosby		RN BSN	University of Alabama at Birmingham Health System and Children's Hospital of Alabama	Birmingham, AL	Non-Author Contributor	
Myriam	Peralta-Carcelen		MD MPH	University of Alabama at Birmingham Health System and Children's Hospital of Alabama	Birmingham, AL	Non-Author Contributor	

Supplemental Online Content: Nonauthor Collaborators
*First name, last name, and suffix (if applicable) are required and will appear in PubMed.

***First Name and Middle Initial(s)**	***Last Name**	***Suffix (eg, Jr, III)**	Academic Degrees	Institution	Location (city, state/province, country)	Role or Contribution, eg, chair, principal investigator	Group (if more than 1 Group listed in the byline) and/or Subgroup (eg, Steering Committee)
Fred J.	Biasini		PhD	University of Alabama at Birmingham Health System and Children's Hospital of Alabama	Birmingham, AL	Non-Author Contributor	
Kristen C.	Johnston		MSN CRNP	University of Alabama at Birmingham Health System and Children's Hospital of Alabama	Birmingham, AL	Non-Author Contributor	
Mary Beth	Moses		PT MS PCS	University of Alabama at Birmingham Health System and Children's Hospital of Alabama	Birmingham, AL	Non-Author Contributor	
Tara E.	McNair		RN BSN	University of Alabama at Birmingham Health System and Children's Hospital of Alabama	Birmingham, AL	Non-Author Contributor	
Vivien A.	Philips		RN BSN	University of Alabama at Birmingham Health System and Children's Hospital of Alabama	Birmingham, AL	Non-Author Contributor	
Richard V.	Rector		PhD	University of Alabama at Birmingham Health System and Children's Hospital of Alabama	Birmingham, AL	Non-Author Contributor	
Sally	Whitley		MA OTR-L FAOTA	University of Alabama at Birmingham Health System and Children's Hospital of Alabama	Birmingham, AL	Non-Author Contributor	
Kristy A.	Domnanovich		PhD	University of Alabama at Birmingham Health System and Children's Hospital of Alabama	Birmingham, AL	Non-Author Contributor	
Sheree	York Chapman		PT DPT PCS	University of Alabama at Birmingham Health System and Children's Hospital of Alabama	Birmingham, AL	Non-Author Contributor	
Robin K.	Whyte		MB	Dalhousie University	Halifax, Nova Scotia, Canada	Non-Author Contributor	
Tarah T.	Colaizy		MD MPH	University of Iowa	Iowa City, IA	Non-Author Contributor	

Supplemental Online Content: Nonauthor Collaborators
*First name, last name, and suffix (if applicable) are required and will appear in PubMed.

***First Name and Middle Initial(s)**	***Last Name**	***Suffix (eg, Jr, III)**	Academic Degrees	Institution	Location (city, state/province, country)	Role or Contribution, eg, chair, principal investigator	Group (if more than 1 Group listed in the byline) and/or Subgroup (eg, Steering Committee)
John A.	Widness		MD	University of Iowa	Iowa City, IA	Non-Author Contributor	
Jane E.	Brumbaugh		MD	University of Iowa	Iowa City, IA	Non-Author Contributor	
Heidi M.	Harmon		MD	University of Iowa	Iowa City, IA	Non-Author Contributor	
Jacky R.	Walker		RN	University of Iowa	Iowa City, IA	Non-Author Contributor	
Claire A.	Goeke		RN	University of Iowa	Iowa City, IA	Non-Author Contributor	
Mendi L.	Schmelzel		MSN RN	University of Iowa	Iowa City, IA	Non-Author Contributor	
Diane L.	Eastman		RN CPNP MA	University of Iowa	Iowa City, IA	Non-Author Contributor	
Conra	Backstrom Lacy		RN	University of New Mexico Health Sciences Center	Albuquerque, NM	Non-Author Contributor	
Janell	Fuller		MD	University of New Mexico Health Sciences Center	Albuquerque, NM	Non-Author Contributor	
Mary	Hanson		RN BSN	University of New Mexico Health Sciences Center	Albuquerque, NM	Non-Author Contributor	
Carol	Hartenberger		BSN MPH	University of New Mexico Health Sciences Center	Albuquerque, NM	Non-Author Contributor	
Elizabeth	Kuan		RN BSN	University of New Mexico Health Sciences Center	Albuquerque, NM	Non-Author Contributor	
Jean R.	Lowe		PhD	University of New Mexico Health Sciences Center	Albuquerque, NM	Non-Author Contributor	
Sandra	Sundquist Beauman		MSN RNC	University of New Mexico Health Sciences Center	Albuquerque, NM	Non-Author Contributor	

Supplemental Online Content: Nonauthor Collaborators
*First name, last name, and suffix (if applicable) are required and will appear in PubMed.

***First Name and Middle Initial(s)**	***Last Name**	***Suffix (eg, Jr, III)**	Academic Degrees	Institution	Location (city, state/province, country)	Role or Contribution, eg, chair, principal investigator	Group (if more than 1 Group listed in the byline) and/or Subgroup (eg, Steering Committee)
Eric C.	Eichenwald		MD	University of Pennsylvania, Hospital of the University of Pennsylvania, Pennsylvania Hospital, and Children's Hospital of Philadelphia	Philadelphia, PA	Non-Author Contributor	
Barbara	Schmidt		MD MSc	University of Pennsylvania, Hospital of the University of Pennsylvania, Pennsylvania Hospital, and Children's Hospital of Philadelphia	Philadelphia, PA	Non-Author Contributor	
Soraya	Abbasi		MD	University of Pennsylvania, Hospital of the University of Pennsylvania, Pennsylvania Hospital, and Children's Hospital of Philadelphia	Philadelphia, PA	Non-Author Contributor	
Aasma S.	Chaudhary		BS RRT	University of Pennsylvania, Hospital of the University of Pennsylvania, Pennsylvania Hospital, and Children's Hospital of Philadelphia	Philadelphia, PA	Non-Author Contributor	
Dara M.	Cucinotta		RN	University of Pennsylvania, Hospital of the University of Pennsylvania, Pennsylvania Hospital, and Children's Hospital of Philadelphia	Philadelphia, PA	Non-Author Contributor	
Judy C.	Bernbaum		MD	University of Pennsylvania, Hospital of the University of Pennsylvania, Pennsylvania Hospital, and Children's Hospital of Philadelphia	Philadelphia, PA	Non-Author Contributor	

Supplemental Online Content: Nonauthor Collaborators
*First name, last name, and suffix (if applicable) are required and will appear in PubMed.

***First Name and Middle Initial(s)**	***Last Name**	***Suffix (eg, Jr, III)**	Academic Degrees	Institution	Location (city, state/province, country)	Role or Contribution, eg, chair, principal investigator	Group (if more than 1 Group listed in the byline) and/or Subgroup (eg, Steering Committee)
Marsha	Gerdes		PhD	University of Pennsylvania, Hospital of the University of Pennsylvania, Pennsylvania Hospital, and Children's Hospital of Philadelphia	Philadelphia, PA	Non-Author Contributor	
Hallam	Hurt		MD	University of Pennsylvania, Hospital of the University of Pennsylvania, Pennsylvania Hospital, and Children's Hospital of Philadelphia	Philadelphia, PA	Non-Author Contributor	
Jonathan	Snyder		RN BSN	University of Pennsylvania, Hospital of the University of Pennsylvania, Pennsylvania Hospital, and Children's Hospital of Philadelphia	Philadelphia, PA	Non-Author Contributor	
Kris	Ziolkowski		CMA (AAMA) CCRP	University of Pennsylvania, Hospital of the University of Pennsylvania, Pennsylvania Hospital, and Children's Hospital of Philadelphia	Philadelphia, PA	Non-Author Contributor	
Carl T.	D'Angio		MD	University of Rochester Medical Center, Golisano Children's Hospital, and the University of Buffalo Women's and Children's Hospital of Buffalo	Rochester and Buffalo, NY	Non-Author Contributor	
Ronnie	Guillet		MD PhD	University of Rochester Medical Center, Golisano Children's Hospital, and the University of Buffalo Women's and Children's Hospital of Buffalo	Rochester and Buffalo, NY	Non-Author Contributor	

Supplemental Online Content: Nonauthor Collaborators
*First name, last name, and suffix (if applicable) are required and will appear in PubMed.

***First Name and Middle Initial(s)**	***Last Name**	***Suffix (eg, Jr, III)**	Academic Degrees	Institution	Location (city, state/province, country)	Role or Contribution, eg, chair, principal investigator	Group (if more than 1 Group listed in the byline) and/or Subgroup (eg, Steering Committee)
Melissa F.	Carmen		MD	University of Rochester Medical Center, Golisano Children's Hospital, and the University of Buffalo Women's and Children's Hospital of Buffalo	Rochester and Buffalo, NY	Non-Author Contributor	
Gary J.	Myers		MD	University of Rochester Medical Center, Golisano Children's Hospital, and the University of Buffalo Women's and Children's Hospital of Buffalo	Rochester and Buffalo, NY	Non-Author Contributor	
Kyle	Binion		BS	University of Rochester Medical Center, Golisano Children's Hospital, and the University of Buffalo Women's and Children's Hospital of Buffalo	Rochester and Buffalo, NY	Non-Author Contributor	
Melissa	Bowman		RN NP	University of Rochester Medical Center, Golisano Children's Hospital, and the University of Buffalo Women's and Children's Hospital of Buffalo	Rochester and Buffalo, NY	Non-Author Contributor	
Elizabeth	Boylin		BA	University of Rochester Medical Center, Golisano Children's Hospital, and the University of Buffalo Women's and Children's Hospital of Buffalo	Rochester and Buffalo, NY	Non-Author Contributor	
Caitlin	Fallone		MA	University of Rochester Medical Center, Golisano Children's Hospital, and the University of Buffalo Women's and Children's Hospital of Buffalo	Rochester and Buffalo, NY	Non-Author Contributor	

Supplemental Online Content: Nonauthor Collaborators
*First name, last name, and suffix (if applicable) are required and will appear in PubMed.

***First Name and Middle Initial(s)**	***Last Name**	***Suffix (eg, Jr, III)**	Academic Degrees	Institution	Location (city, state/province, country)	Role or Contribution, eg, chair, principal investigator	Group (if more than 1 Group listed in the byline) and/or Subgroup (eg, Steering Committee)
Osman	Farooq		MD	University of Rochester Medical Center, Golisano Children's Hospital, and the University of Buffalo Women's and Children's Hospital of Buffalo	Rochester and Buffalo, NY	Non-Author Contributor	
Julianne	Hunn		MSHCM	University of Rochester Medical Center, Golisano Children's Hospital, and the University of Buffalo Women's and Children's Hospital of Buffalo	Rochester and Buffalo, NY	Non-Author Contributor	
Rosemary L.	Jensen			University of Rochester Medical Center, Golisano Children's Hospital, and the University of Buffalo Women's and Children's Hospital of Buffalo	Rochester and Buffalo, NY	Non-Author Contributor	
Rachel	Jones			University of Rochester Medical Center, Golisano Children's Hospital, and the University of Buffalo Women's and Children's Hospital of Buffalo	Rochester and Buffalo, NY	Non-Author Contributor	
Joan	Merzbach		LMSW	University of Rochester Medical Center, Golisano Children's Hospital, and the University of Buffalo Women's and Children's Hospital of Buffalo	Rochester and Buffalo, NY	Non-Author Contributor	
Constance	Orne			University of Rochester Medical Center, Golisano Children's Hospital, and the University of Buffalo Women's and Children's Hospital of Buffalo	Rochester and Buffalo, NY	Non-Author Contributor	

Supplemental Online Content: Nonauthor Collaborators
*First name, last name, and suffix (if applicable) are required and will appear in PubMed.

***First Name and Middle Initial(s)**	***Last Name**	***Suffix (eg, Jr, III)**	Academic Degrees	Institution	Location (city, state/province, country)	Role or Contribution, eg, chair, principal investigator	Group (if more than 1 Group listed in the byline) and/or Subgroup (eg, Steering Committee)
Premini	Sabaratnam		MPH	University of Rochester Medical Center, Golisano Children's Hospital, and the University of Buffalo Women's and Children's Hospital of Buffalo	Rochester and Buffalo, NY	Non-Author Contributor	
Ann Marie	Scorsone		MS CCRC	University of Rochester Medical Center, Golisano Children's Hospital, and the University of Buffalo Women's and Children's Hospital of Buffalo	Rochester and Buffalo, NY	Non-Author Contributor	
Holly I.M.	Wadkins			University of Rochester Medical Center, Golisano Children's Hospital, and the University of Buffalo Women's and Children's Hospital of Buffalo	Rochester and Buffalo, NY	Non-Author Contributor	
Kelly	Yost		PhD	University of Rochester Medical Center, Golisano Children's Hospital, and the University of Buffalo Women's and Children's Hospital of Buffalo	Rochester and Buffalo, NY	Non-Author Contributor	
Satyan	Lakshminrusimha		MD	University of Rochester Medical Center, Golisano Children's Hospital, and the University of Buffalo Women's and Children's Hospital of Buffalo	Rochester and Buffalo, NY	Non-Author Contributor	
Stephanie	Guilford		BS	University of Rochester Medical Center, Golisano Children's Hospital, and the University of Buffalo Women's and Children's Hospital of Buffalo	Rochester and Buffalo, NY	Non-Author Contributor	

Supplemental Online Content: Nonauthor Collaborators
*First name, last name, and suffix (if applicable) are required and will appear in PubMed.

***First Name and Middle Initial(s)**	***Last Name**	***Suffix (eg, Jr, III)**	Academic Degrees	Institution	Location (city, state/province, country)	Role or Contribution, eg, chair, principal investigator	Group (if more than 1 Group listed in the byline) and/or Subgroup (eg, Steering Committee)
Michelle E.	Hartley-McAndrews		MD	University of Rochester Medical Center, Golisano Children's Hospital, and the University of Buffalo Women's and Children's Hospital of Buffalo	Rochester and Buffalo, NY	Non-Author Contributor	
Ashley	Williams		MSEd	University of Rochester Medical Center, Golisano Children's Hospital, and the University of Buffalo Women's and Children's Hospital of Buffalo	Rochester and Buffalo, NY	Non-Author Contributor	
William	Zorn		PhD	University of Rochester Medical Center, Golisano Children's Hospital, and the University of Buffalo Women's and Children's Hospital of Buffalo	Rochester and Buffalo, NY	Non-Author Contributor	
Emily	Li		BA	University of Rochester Medical Center, Golisano Children's Hospital, and the University of Buffalo Women's and Children's Hospital of Buffalo	Rochester and Buffalo, NY	Non-Author Contributor	
Jennifer	Donato		BS	University of Rochester Medical Center, Golisano Children's Hospital, and the University of Buffalo Women's and Children's Hospital of Buffalo	Rochester and Buffalo, NY	Non-Author Contributor	
Kimberly G.	McKee		BS	University of Rochester Medical Center, Golisano Children's Hospital, and the University of Buffalo Women's and Children's Hospital of Buffalo	Rochester and Buffalo, NY	Non-Author Contributor	

Supplemental Online Content: Nonauthor Collaborators
*First name, last name, and suffix (if applicable) are required and will appear in PubMed.

***First Name and Middle Initial(s)**	***Last Name**	***Suffix (eg, Jr, III)**	Academic Degrees	Institution	Location (city, state/province, country)	Role or Contribution, eg, chair, principal investigator	Group (if more than 1 Group listed in the byline) and/or Subgroup (eg, Steering Committee)
Kelly R.	Coleman		PsyD	University of Rochester Medical Center, Golisano Children's Hospital, and the University of Buffalo Women's and Children's Hospital of Buffalo	Rochester and Buffalo, NY	Non-Author Contributor	
Alison	Kent		BMBS FRACP MD	University of Rochester Medical Center, Golisano Children's Hospital, and the University of Buffalo Women's and Children's Hospital of Buffalo	Rochester and Buffalo, NY	Non-Author Contributor	
Luc P.	Brion		MD	University of Texas Southwestern Medical Center, Parkland Health & Hospital System, and Children's Medical Center Dallas	Dallas, TX	Non-Author Contributor	
Roy J.	Heyne		MD	University of Texas Southwestern Medical Center, Parkland Health & Hospital System, and Children's Medical Center Dallas	Dallas, TX	Non-Author Contributor	
Diana M.	Vasil		MSN BSN RNC-NIC	University of Texas Southwestern Medical Center, Parkland Health & Hospital System, and Children's Medical Center Dallas	Dallas, TX	Non-Author Contributor	
Sally S.	Adams		MS RN CPNP	University of Texas Southwestern Medical Center, Parkland Health & Hospital System, and Children's Medical Center Dallas	Dallas, TX	Non-Author Contributor	
Maria M.	De Leon		RN	University of Texas Southwestern Medical Center, Parkland Health & Hospital System, and Children's Medical Center Dallas	Dallas, TX	Non-Author Contributor	

Supplemental Online Content: Nonauthor Collaborators
*First name, last name, and suffix (if applicable) are required and will appear in PubMed.

***First Name and Middle Initial(s)**	***Last Name**	***Suffix (eg, Jr, III)**	Academic Degrees	Institution	Location (city, state/province, country)	Role or Contribution, eg, chair, principal investigator	Group (if more than 1 Group listed in the byline) and/or Subgroup (eg, Steering Committee)
Francis	Eubanks		RN BSN	University of Texas Southwestern Medical Center, Parkland Health & Hospital System, and Children's Medical Center Dallas	Dallas, TX	Non-Author Contributor	
Alicia	Guzman			University of Texas Southwestern Medical Center, Parkland Health & Hospital System, and Children's Medical Center Dallas	Dallas, TX	Non-Author Contributor	
Elizabeth	Heyne		PsyD PA-C	University of Texas Southwestern Medical Center, Parkland Health & Hospital System, and Children's Medical Center Dallas	Dallas, TX	Non-Author Contributor	
Lizette E.	Lee		RN	University of Texas Southwestern Medical Center, Parkland Health & Hospital System, and Children's Medical Center Dallas	Dallas, TX	Non-Author Contributor	
Linda A.	Madden		BSN RN CPNP	University of Texas Southwestern Medical Center, Parkland Health & Hospital System, and Children's Medical Center Dallas	Dallas, TX	Non-Author Contributor	
E. Rebecca	McDougald		MSN APRN CPNP-PC/AC	University of Texas Southwestern Medical Center, Parkland Health & Hospital System, and Children's Medical Center Dallas	Dallas, TX	Non-Author Contributor	
Lara	Pavageau		MD	University of Texas Southwestern Medical Center, Parkland Health & Hospital System, and Children's Medical Center Dallas	Dallas, TX	Non-Author Contributor	

Supplemental Online Content: Nonauthor Collaborators
*First name, last name, and suffix (if applicable) are required and will appear in PubMed.

***First Name and Middle Initial(s)**	***Last Name**	***Suffix (eg, Jr, III)**	Academic Degrees	Institution	Location (city, state/province, country)	Role or Contribution, eg, chair, principal investigator	Group (if more than 1 Group listed in the byline) and/or Subgroup (eg, Steering Committee)
Pollieanna	Sepulveda		RN	University of Texas Southwestern Medical Center, Parkland Health & Hospital System, and Children's Medical Center Dallas	Dallas, TX	Non-Author Contributor	
Cathy	Twell Boatman		MS CIMI	University of Texas Southwestern Medical Center, Parkland Health & Hospital System, and Children's Medical Center Dallas	Dallas, TX	Non-Author Contributor	
Kristine	Tolentino-Plata		MS	University of Texas Southwestern Medical Center, Parkland Health & Hospital System, and Children's Medical Center Dallas	Dallas, TX	Non-Author Contributor	
Azucena	Vera		AS	University of Texas Southwestern Medical Center, Parkland Health & Hospital System, and Children's Medical Center Dallas	Dallas, TX	Non-Author Contributor	
Jillian	Waterbury		DNP RN CPNP-PC	University of Texas Southwestern Medical Center, Parkland Health & Hospital System, and Children's Medical Center Dallas	Dallas, TX	Non-Author Contributor	
Bradley A.	Yoder		MD	University of Utah Medical Center, Intermountain Medical Center, McKay-Dee Hospital, Utah Valley Hospital, and Primary Children's Hospital	Murray, Ogden, Provo, and Salt Lake City, UT	Non-Author Contributor	
Stephen. D.	Minton		MD	University of Utah Medical Center, Intermountain Medical Center, McKay-Dee Hospital, Utah Valley Hospital, and Primary Children's Hospital	Murray, Ogden, Provo, and Salt Lake City, UT	Non-Author Contributor	

Supplemental Online Content: Nonauthor Collaborators
*First name, last name, and suffix (if applicable) are required and will appear in PubMed.

***First Name and Middle Initial(s)**	***Last Name**	***Suffix (eg, Jr, III)**	Academic Degrees	Institution	Location (city, state/province, country)	Role or Contribution, eg, chair, principal investigator	Group (if more than 1 Group listed in the byline) and/or Subgroup (eg, Steering Committee)
Mark J.	Sheffield		MD	University of Utah Medical Center, Intermountain Medical Center, McKay-Dee Hospital, Utah Valley Hospital, and Primary Children's Hospital	Murray, Ogden, Provo, and Salt Lake City, UT	Non-Author Contributor	
Carrie A.	Rau		RN BSN CCRC	University of Utah Medical Center, Intermountain Medical Center, McKay-Dee Hospital, Utah Valley Hospital, and Primary Children's Hospital	Murray, Ogden, Provo, and Salt Lake City, UT	Non-Author Contributor	
Sarah	Winter		MD	University of Utah Medical Center, Intermountain Medical Center, McKay-Dee Hospital, Utah Valley Hospital, and Primary Children's Hospital	Murray, Ogden, Provo, and Salt Lake City, UT	Non-Author Contributor	
Shawna	Baker		RN	University of Utah Medical Center, Intermountain Medical Center, McKay-Dee Hospital, Utah Valley Hospital, and Primary Children's Hospital	Murray, Ogden, Provo, and Salt Lake City, UT	Non-Author Contributor	
Jill	Burnett		RN BSN	University of Utah Medical Center, Intermountain Medical Center, McKay-Dee Hospital, Utah Valley Hospital, and Primary Children's Hospital	Murray, Ogden, Provo, and Salt Lake City, UT	Non-Author Contributor	
Susan	Christensen		RN	University of Utah Medical Center, Intermountain Medical Center, McKay-Dee Hospital, Utah Valley Hospital, and Primary Children's Hospital	Murray, Ogden, Provo, and Salt Lake City, UT	Non-Author Contributor	

Supplemental Online Content: Nonauthor Collaborators
*First name, last name, and suffix (if applicable) are required and will appear in PubMed.

***First Name and Middle Initial(s)**	***Last Name**	***Suffix (eg, Jr, III)**	Academic Degrees	Institution	Location (city, state/province, country)	Role or Contribution, eg, chair, principal investigator	Group (if more than 1 Group listed in the byline) and/or Subgroup (eg, Steering Committee)
Laura	Cole Bledsoe		RN	University of Utah Medical Center, Intermountain Medical Center, McKay-Dee Hospital, Utah Valley Hospital, and Primary Children's Hospital	Murray, Ogden, Provo, and Salt Lake City, UT	Non-Author Contributor	
Sean	Cunningham		PhD	University of Utah Medical Center, Intermountain Medical Center, McKay-Dee Hospital, Utah Valley Hospital, and Primary Children's Hospital	Murray, Ogden, Provo, and Salt Lake City, UT	Non-Author Contributor	
Jennifer O.	Elmont		RN BSN	University of Utah Medical Center, Intermountain Medical Center, McKay-Dee Hospital, Utah Valley Hospital, and Primary Children's Hospital	Murray, Ogden, Provo, and Salt Lake City, UT	Non-Author Contributor	
Becky	Hall		APRN	University of Utah Medical Center, Intermountain Medical Center, McKay-Dee Hospital, Utah Valley Hospital, and Primary Children's Hospital	Murray, Ogden, Provo, and Salt Lake City, UT	Non-Author Contributor	
Trisha	Marshant		RN	University of Utah Medical Center, Intermountain Medical Center, McKay-Dee Hospital, Utah Valley Hospital, and Primary Children's Hospital	Murray, Ogden, Provo, and Salt Lake City, UT	Non-Author Contributor	
Earl	Maxson		RN CCRN	University of Utah Medical Center, Intermountain Medical Center, McKay-Dee Hospital, Utah Valley Hospital, and Primary Children's Hospital	Murray, Ogden, Provo, and Salt Lake City, UT	Non-Author Contributor	

Supplemental Online Content: Nonauthor Collaborators
*First name, last name, and suffix (if applicable) are required and will appear in PubMed.

***First Name and Middle Initial(s)**	***Last Name**	***Suffix (eg, Jr, III)**	Academic Degrees	Institution	Location (city, state/province, country)	Role or Contribution, eg, chair, principal investigator	Group (if more than 1 Group listed in the byline) and/or Subgroup (eg, Steering Committee)
Kandace M.	McGrath			University of Utah Medical Center, Intermountain Medical Center, McKay-Dee Hospital, Utah Valley Hospital, and Primary Children's Hospital	Murray, Ogden, Provo, and Salt Lake City, UT	Non-Author Contributor	
Hena G.	Mickelsen		BA	University of Utah Medical Center, Intermountain Medical Center, McKay-Dee Hospital, Utah Valley Hospital, and Primary Children's Hospital	Murray, Ogden, Provo, and Salt Lake City, UT	Non-Author Contributor	
Galina	Morshedzadeh		BSN APRN	University of Utah Medical Center, Intermountain Medical Center, McKay-Dee Hospital, Utah Valley Hospital, and Primary Children's Hospital	Murray, Ogden, Provo, and Salt Lake City, UT	Non-Author Contributor	
D. Melody	Parry		RN BSN	University of Utah Medical Center, Intermountain Medical Center, McKay-Dee Hospital, Utah Valley Hospital, and Primary Children's Hospital	Murray, Ogden, Provo, and Salt Lake City, UT	Non-Author Contributor	
Brixen A.	Reich		MSN RNC CCRC	University of Utah Medical Center, Intermountain Medical Center, McKay-Dee Hospital, Utah Valley Hospital, and Primary Children's Hospital	Murray, Ogden, Provo, and Salt Lake City, UT	Non-Author Contributor	
Susan T.	Schaefer		RN BSN RRT	University of Utah Medical Center, Intermountain Medical Center, McKay-Dee Hospital, Utah Valley Hospital, and Primary Children's Hospital	Murray, Ogden, Provo, and Salt Lake City, UT	Non-Author Contributor	

Supplemental Online Content: Nonauthor Collaborators
*First name, last name, and suffix (if applicable) are required and will appear in PubMed.

***First Name and Middle Initial(s)**	***Last Name**	***Suffix (eg, Jr, III)**	Academic Degrees	Institution	Location (city, state/province, country)	Role or Contribution, eg, chair, principal investigator	Group (if more than 1 Group listed in the byline) and/or Subgroup (eg, Steering Committee)
Ashley L.	Stuart		PhD	University of Utah Medical Center, Intermountain Medical Center, McKay-Dee Hospital, Utah Valley Hospital, and Primary Children's Hospital	Murray, Ogden, Provo, and Salt Lake City, UT	Non-Author Contributor	
Kelly	Stout		PhD	University of Utah Medical Center, Intermountain Medical Center, McKay-Dee Hospital, Utah Valley Hospital, and Primary Children's Hospital	Murray, Ogden, Provo, and Salt Lake City, UT	Non-Author Contributor	
Kimberlee	Weaver-Lewis		RN MS	University of Utah Medical Center, Intermountain Medical Center, McKay-Dee Hospital, Utah Valley Hospital, and Primary Children's Hospital	Murray, Ogden, Provo, and Salt Lake City, UT	Non-Author Contributor	
Kathryn D.	Woodbury		RN BSN	University of Utah Medical Center, Intermountain Medical Center, McKay-Dee Hospital, Utah Valley Hospital, and Primary Children's Hospital	Murray, Ogden, Provo, and Salt Lake City, UT	Non-Author Contributor	
Seetha	Shankaran		MD	Wayne State University, Hutzel Women's Hospital, and Children's Hospital of Michigan	Detroit, MI	Non-Author Contributor	
Rebecca	Bara		RN BSN	Wayne State University, Hutzel Women's Hospital, and Children's Hospital of Michigan	Detroit, MI	Non-Author Contributor	
Prashant	Agarwal		MD	Wayne State University, Hutzel Women's Hospital, and Children's Hospital of Michigan	Detroit, MI	Non-Author Contributor	

Supplemental Online Content: Nonauthor Collaborators
*First name, last name, and suffix (if applicable) are required and will appear in PubMed.

***First Name and Middle Initial(s)**	***Last Name**	***Suffix (eg, Jr, III)**	Academic Degrees	Institution	Location (city, state/province, country)	Role or Contribution, eg, chair, principal investigator	Group (if more than 1 Group listed in the byline) and/or Subgroup (eg, Steering Committee)
Monika	Bajaj		MD	Wayne State University, Hutzel Women's Hospital, and Children's Hospital of Michigan	Detroit, MI	Non-Author Contributor	
Kirsten	Childs		RN BSN	Wayne State University, Hutzel Women's Hospital, and Children's Hospital of Michigan	Detroit, MI	Non-Author Contributor	
Melissa	February		MD	Wayne State University, Hutzel Women's Hospital, and Children's Hospital of Michigan	Detroit, MI	Non-Author Contributor	
Laura	Goldston		MA	Wayne State University, Hutzel Women's Hospital, and Children's Hospital of Michigan	Detroit, MI	Non-Author Contributor	
Mary E.	Johnson		RN BSN	Wayne State University, Hutzel Women's Hospital, and Children's Hospital of Michigan	Detroit, MI	Non-Author Contributor	
Girija	Natarajan		MD	Wayne State University, Hutzel Women's Hospital, and Children's Hospital of Michigan	Detroit, MI	Non-Author Contributor	
Bogdan	Panaitescu		MD PhD	Wayne State University, Hutzel Women's Hospital, and Children's Hospital of Michigan	Detroit, MI	Non-Author Contributor	
Eunice	Woldt		RN MSN	Wayne State University, Hutzel Women's Hospital, and Children's Hospital of Michigan	Detroit, MI	Non-Author Contributor	
