# Supplementary material for: Tissue Oxygenation Changes After Transfusion and Outcomes in Preterm Infants: A Secondary Near-Infrared Spectroscopy Study of the Transfusion of Prematures Randomized Clinical Trial (TOP NIRS)
Source: JAMA Netw Open. 2023 Sep 21;6(9):e2334889. doi: 10.1001/jamanetworkopen.2023.34889 (PMC10514737; doi:10.1001/jamanetworkopen.2023.34889)
Supplement: Supplement 4. — Data Sharing Statement [file jamanetwopen-e2334889-s004.pdf]

Data Sharing Statement

Chock. Tissue Oxygenation Changes After Transfusion and Outcomes in Preterm Infants. *JAMA Netw Open*. Published September 21, 2023. doi:10.1001/jamanetworkopen.2023.34889

Data
Data available: Yes
Data types: Deidentified participant data
How to access data: Data reported in this paper is planned for release to the NHLBI Biospecimen and Data Repository at https://biolincc.nhlbi.nih.gov/home/. If required sooner, this data may be requested through a data use agreement. Further details are available at https://neonatal.rti.org/index.cfm?fuseaction=DataRequest.Home
When available: With publication

Supporting Documents
Document types: None

Additional Information
Who can access the data: Researchers whose proposed use of the data has been approved
Types of analyses: For a specified purpose
Mechanisms of data availability: Data will be made available after approval of proposal and with data use agreement
